# Exploring a Gemcitabine-Glucose Hybrid as a Glycoconjugate Prodrug

**DOI:** 10.1021/acsomega.4c02417

**Published:** 2024-07-09

**Authors:** Jack Porter, Amanda R. Noble, Nathalie Signoret, Martin A. Fascione, Gavin J. Miller

**Affiliations:** † Centre for Glycoscience and School of Chemical and Physical Sciences, Keele University, Keele, Staffordshire ST5 5BG, United Kingdom; ‡ Hull York Medical School, University of York, Heslington, York YO10 5DD, U.K.; § Department of Chemistry, University of York, Heslington, York YO10 5DD, U.K.

## Abstract

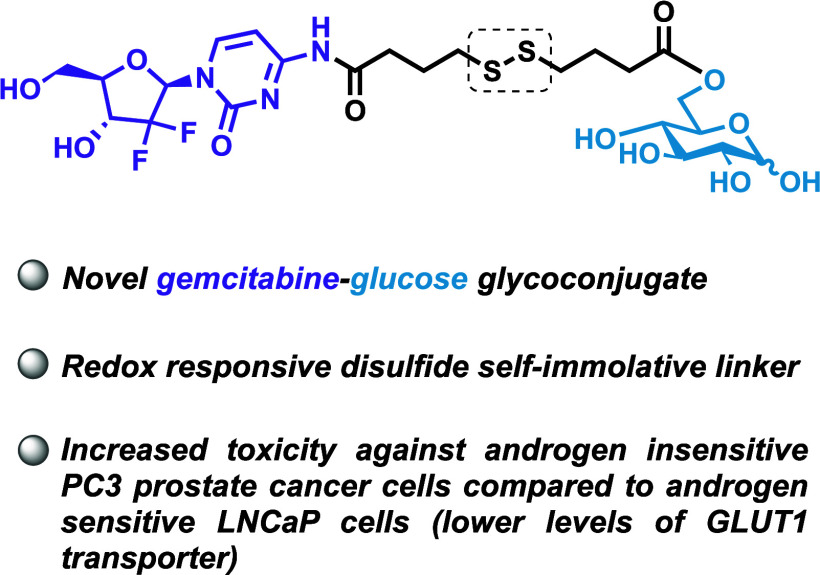

Nucleoside analogues are established treatments for cancer and viral infection. Gemcitabine is a commonly employed nucleoside analogue displaying anticancer properties against a range of tumor types but is rapidly inactivated *in vivo*. Efforts to bolster its pharmaceutical profile include investigating prodrug forms. Herein, we explore the synthesis of a novel glucose-gemcitabine glycoconjugate, targeting uptake via glucose transport. We select a redox-reactive disulfide linker for conjugation of gemcitabine (through *N*4-cytosine) with glucose. Evaluation of this glycoconjugate reveals increased toxicity against androgen insensitive PC3 prostate cancer cells compared to LNCaP (which have lower levels of glucose transporter GLUT1). These preliminary results suggest that glycoconjugation of nucleosides may be an effective approach to targeting cells which display increased uptake and metabolism of glucose.

## Introduction

Prodrugs are molecules with little or no pharmacological activity that are converted to the active parent drug *in vivo* by enzymatic or chemical reactions (or through a combination of the two). Since their inception, prodrugs have evolved from a niche corner of discovery research to being intentionally designed. In the past 10 years, the US Food and Drug Administration has approved at least 30 prodrugs, which accounts for more than 12% of all approved new small molecule chemical entities.^1^ Continued efforts in this research space can support the avoidance of drug development and administration challenges. For example, those that limit drug formulation and delivery options exert unacceptable biopharmaceutical or pharmacokinetic profiles or show poor targeting. Herein, we explore creating a glycoconjugate of a cytotoxic nucleoside analogue toward a new prodrug concept.

**Figure 1:**
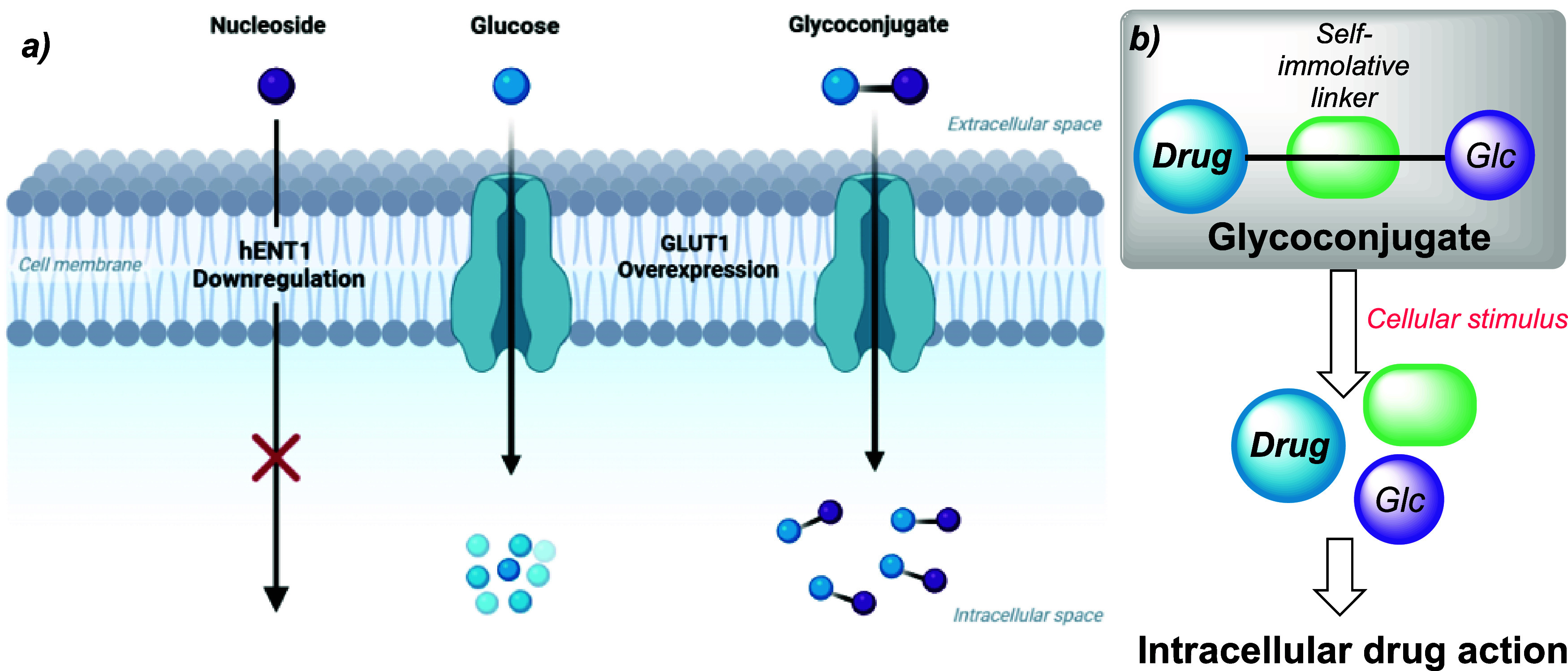
(a) Targeting nucleoside analogue transport into cells using a glucose-nucleoside analogue glycoconjugate. (b) Glycoconjugate design based upon a responsive cellular trigger to release both glucose and drug via a self-immolative linker.

Nucleoside analogues possess a privileged and accomplished history within therapeutic intervention strategies, most notably in the fight against viruses and cancer.^2,3^ Furthermore, several successful prodrug forms have emerged from this pharmaceutical drug class, typified by sofosbuvir, tenofovir alafenamide, and remdesivir, each harnessing the ProTide prodrug technology developed by McGuigan.^4,46^ Gemcitabine (2′,2′-difluorodeoxycytidine) is an intravenously dosed pyrimidine nucleoside analogue with proven anticancer activity against a variety of solid tumor types.^5^ Despite its clinical efficacy, gemcitabine is rapidly inactivated by deoxycytidine deaminase, which is present at high levels in both human plasma and the liver.^6^ Accordingly, approaches have been developed to access prodrug forms of gemcitabine. Selected examples include an *N*4-cytosine valproate amide,^7^ C5′-amino acid-derived esters,^8,9^ and a Hoechst conjugate.^10^ These methods sought peptide transporters and extracellular DNA binding as pathways for cellular uptake. We were thus intrigued to explore if uptake through glucose transport could be harnessed by creating a glycoconjugate of the nucleoside analogue therapeutic. To accommodate increased requirements for glucose, tumors typically overexpress essential transporters, e.g., GLUT1 (Figure 1a).^11^ Furthermore, glycoconjugation of therapeutics has been investigated for a number of cytotoxic agents, resulting in favorable therapeutic effects, including arylhyrazone prochelators, DNA alkylators, platinum-based drugs, and glucose-modified mustard Glufosfamide.^12−15^ Recent examples of gemcitabine-based prodrugs include novel cyclic phosphates and lysine-conjugated examples, together with several other prodrug forms.^16−22^ Our proposed glucose-gemcitabine glycoconjugate approach is highlighted in Figure 1b.

## Results and Discussion

### Glucose-Gemcitabine Conjugate Synthesis

**Scheme 1:**
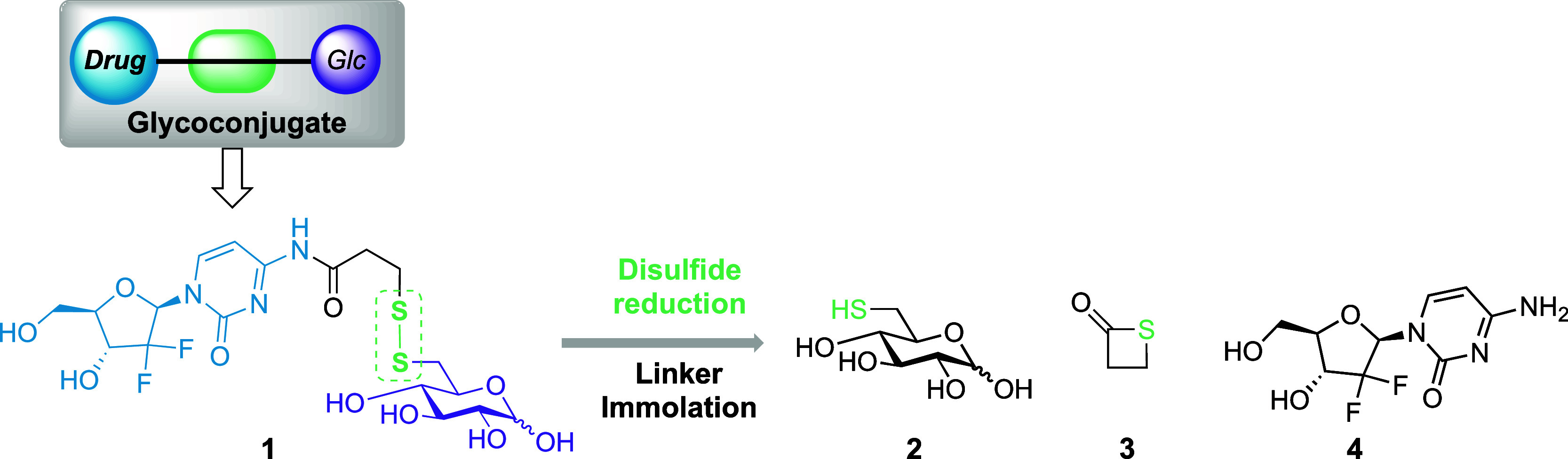
Initial Gemcitabine-Glucose Conjugate Design Upon intracellular disulfide reduction, gemcitabine **4** and benign by-products **2** and **3** are released.

Based on the glycoconjugate design presented in Figure 1b, we targeted the synthesis of gemcitabine-glucose hybrid **1**, incorporating a redox-responsive disulfide as part of the self-immolative linker. We theorized that intracellular reduction of a disulfide [e.g., by glutathione (GSH), cysteine (Cys), or thioredoxin-1 (Trx1)]^23^ could release 6-deoxy-6-thioglucose **2**, β-thiolactam **3**, and gemcitabine **4** (Scheme 1); following disulfide reduction and the release of **2**, intramolecular amide cleavage would form **3** and **4**.^24−26^

**Scheme 2:**
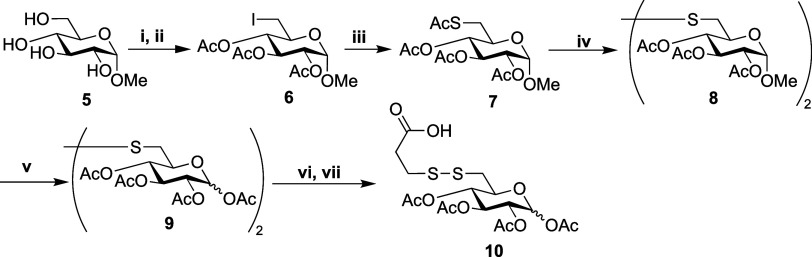
Synthesis of Protected Glucose Ligand **10** Reagents and conditions: (i) I_2_, PPh_3_, imidazole, THF, 70 °C; (ii) Ac_2_O, pyridine, 35 °C (76%, 2 steps); (iii) KSAc, acetone, reflux, 70%; (iv) NIS, I_2_, MeCN, 77%; (v) H_2_SO_4_, AcOH, Ac_2_O, 68%; (vi) DTT, MeCN; (vii) TCCA, THF, NaH, −20 °C to RT, 3-MPA (51%, 2 steps).

Accordingly, we sought to synthesize separate pyranose and nucleoside components and unify them through a self-immolative disulfide linker. Starting from commercially available glucose *O*-methyl glycoside **5**, selective iodination of the primary C6 alcohol was achieved using iodine, imidazole, and triphenylphosphine, followed by addition of pyridine and acetic anhydride to the same pot after 24 h, protecting the remaining hydroxyl groups (Scheme 2) and furnishing iodide **6** in 76% yield over two steps. Subsequent nucleophilic substitution at C6 using potassium thioacetate provided protected glucoside **7** in 70% yield.

Based on work presented by Dong et al.,^27^ we next completed oxidation of C6-SAc **7** using I_2_ and NIS to deliver disulfide **8** in 77% yield; disulfide formation was confirmed by HRMS. Acetolysis of *O*-methyl glycoside **8** was accomplished using a combination of Ac_2_O, AcOH, and H_2_SO_4_, generating an anomeric mixture of tetra-*O*-acetyl disulfide **9** in 68% yield (4:1, α/β). Finally, reduction of compound **9** using dithiothreitol (DTT) enabled completion of asymmetric disulfide synthesis using trichloroisocyanuric acid (TCCA) and 3-mercaptopropionic acid (3-MPA), delivering disulfide **10** in 51% yield over two steps.^28^ Diagnostic ^13^C NMR chemical shifts confirmed inclusion of an unsymmetric disulfide within tetraacetate **10** [δ_C_ 176.9 (C=O), 33.7 (CH_2_), 32.8 (CH_2_) ppm], alongside complementary HRMS.

**Scheme 3:**
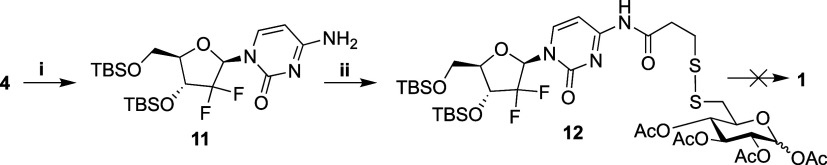
Attempted Synthesis of Glycoconjugate **1** Reagents and conditions: (i) TBSCl, imidazole, DMF, 86%; (ii) EDC, DMAP, DCM, **10**, 65%.

With glucose-based disulfide **10** in hand, our attention shifted to the required coupling partner, an appropriately protected gemcitabine derivative. Preparation of this compound proceeded through 3′,5′-hydroxyl group protection (Scheme 3), accomplished using TBSCl in pyridine with imidazole and delivering bis-silyl ether **11** in 86% yield.^29^

**Figure 2:**
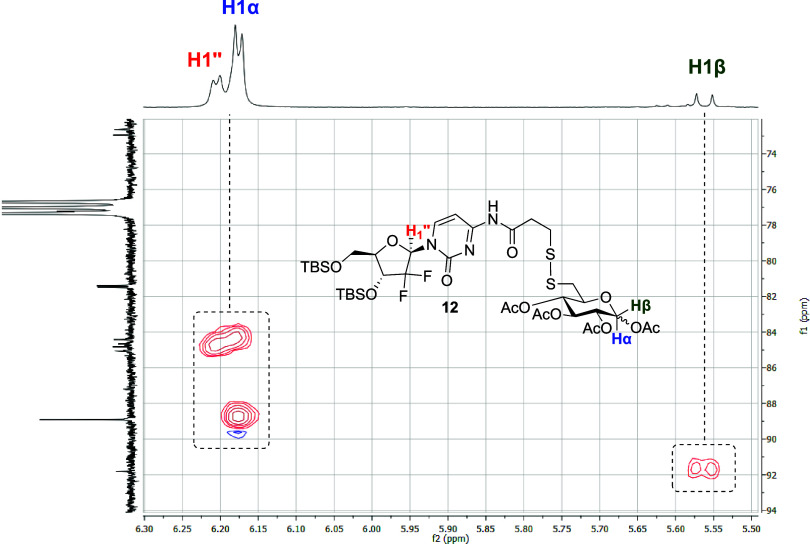
HSQC NMR (400 × 100 MHz, CDCl_3_) data for glycoconjugate **12**, highlighting anomeric shifts for each glucose anomer and the nucleoside.

Next, an amide coupling reaction between **10** and **11** was performed using EDC, successfully yielding glycoconjugate **12** in 65% yield. From the HSQC NMR obtained for **12**, three anomeric environments were observed (Figure 2): one from gemcitabine and two from α/β pyranose, supporting successful protected glycoconjugate synthesis.

Attempts to complete deprotection of conjugate **12** were unsuccessful. Subjecting **12** to Zemplén conditions [Na (0.1 equiv), MeOH, room temp.] revealed several components after 1 h by TLC analysis. Following column chromatography of the crude material, it was evident that C4-pyrimidine amide linker cleavage had occurred. Cleavage of a related C4-NAc cytidine derivative has been reported using 0.1 N NaOH in MeOH,^30^ and we thus screened alternative deprotection conditions (see Table S1), but in each case, similar linker cleavage was observed. As such, a new route was envisioned, obviating the need for protecting group removal as the last steps in the synthesis.

**Scheme 4:**
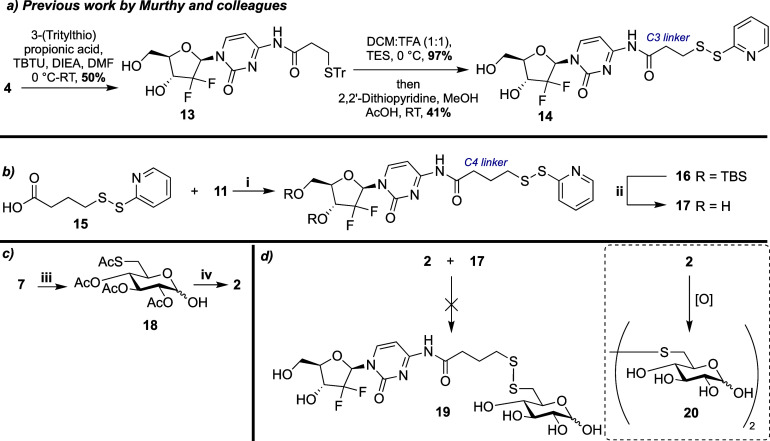
Toward Glycoconjugate **19***via* Thiol–Pyridyl Disulfide Exchange Reagents and conditions: (a) previously reported synthesis of disulfide conjugate **14**;(b) (i) EDC, DMAP, DCM, 80%; (ii) TBAF, THF, 0 °C, 66%; (c) (iii) AcOH, H_2_SO_4_, RT, 62%; (iv) Na, MeOH, RT, 85%; (d) unsuccessful pyridyl disulfide thiol exchange reaction.

Accordingly, we sought a pyridyl disulfide-thiol exchange reaction^31^ and pursued synthesis of gemcitabine disulfide derivative **17** alongside 6-deoxy-6-thio-glucose **2** as the appropriate thiol exchange partner. Murthy and co-workers have reported a route toward a similar gemcitabine disulfide **14**, starting from **4** and directly coupling a protected thiol unit to C4 of cytidine to furnish **13**, followed by deprotection and disulfide formation in three steps and 20% overall yield (Scheme 4a).^10^ Herein, 4-(2-pyridyldithio) butanoic acid **15** was synthesized and attached to TBS-protected gemcitabine **11** using EDC, delivering **16** in 80% yield. With the disulfide in place, deprotection of the silyl groups was achieved using TBAF in THF, providing **17** in 66% yield (Scheme 4b). Noting that our system is differentiated through a four-carbon linker attached through C4 of cytidine, these routes to related disulfide gemcitabine conjugates are comparable, with synthesis of **17** achieved in three steps from **4** in 45% overall yield. Synthesis of 6-deoxy-6-thio-glucose **2** utilized materials from the previous route. Starting from thioacetate **7**, anomeric OMe deprotection was accomplished using AcOH and H_2_SO_4_ to generate hemiacetal **18** as a mixture of anomers (6:1, α/β). Global deacetylation under Zemplén conditions successfully removed the acetates, giving free sugar **2** in 85% yield (Scheme 4c). A protecting group free thiol exchange reaction between **2** and **17** was then attempted (Scheme 4d). After stirring in MeCN for 24 h, TLC and HRMS analysis suggested that the desired reaction had not taken place, but oxidation of **2** to the corresponding disulfide **20** had occurred instead, preventing formation of **19**. As such, we altered our synthetic strategy to incorporate the disulfide prior to conjugation with glucose.

**Scheme 5:**
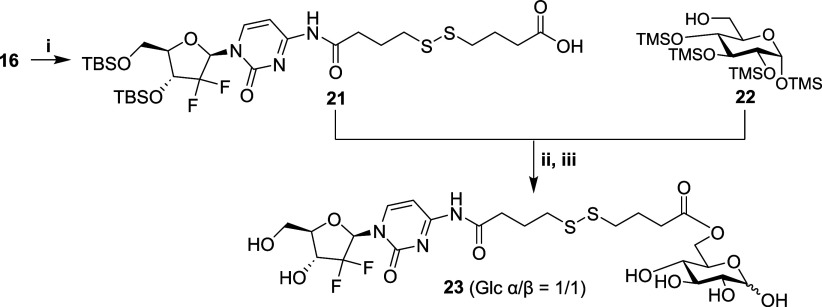
Synthesis of Glycoconjugate **23** Reagents and conditions: (i) 4-mercaptobutyric acid, MeCN, RT, 61%; (ii) EDC, DMAP, RT, 45%; (iii) TBAF, THF, 0 °C, 54%.

To achieve this, previously synthesized pyridyl disulfide **16** was reacted with 4-mercapto butyric acid, delivering **21** in 61% yield (Scheme 5) and supporting the capability of the thiol exchange reaction using components not susceptible to *in situ* oxidation. As ester protecting group removal had proven incompatible with a pyrimidine *N*4-amide linkage (Scheme 3), silicon protecting groups were chosen for the glycoside component, matching those of the nucleoside and allowing for one-step global desilylation, following coupling of the pyranose to gemcitabine. TMS glycoside **22** was thus synthesized and EDC-mediated coupling between **21** and **22** successfully yielded a protected glycoconjugate in 45% yield. Global deprotection using TBAF in THF was successful and enabled isolation and characterization of the free glycoconjugate **23** in 54% yield (18 mg). ^1^H NMR data associated with anomeric centers for **23** integrated as anticipated (H1″:H1α:H1β, 2:1:1). ^19^F NMR revealed two chemical shifts [δ_F_ −119.18 (dd, *J* = 240.0, 12.5 Hz), −120.10 (d, *J* = 244.7 Hz) ppm] corresponding to gem-difluorination at C2″ and HRMS-supported retention of the disulfide linkage.

### Glucose-Gemcitabine Conjugate Evaluation

**Figure 3:**
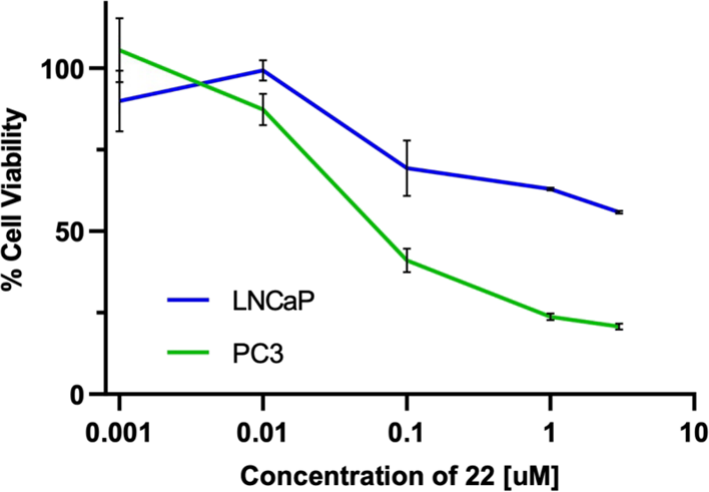
MTS cell viability assay measuring the formazan product formed at 490 nm in metabolically active cells following treatment of PC3 or LNCaP cells with varying concentrations of **23** for 72 h.

Prostate cancer is the most common cancer in men with around 12,000 deaths per year in the UK.^32^ Treatments are available to slow progression, such as surgery, radiotherapy, androgen deprivation therapy, and combinations of these. However, invariably, the cancer becomes resistant, leading to castration-resistant prostate cancer (CRPC). There remains therefore an urgent need for more treatment options, particularly against CRPC. Glucose transporter 1 (GLUT1) regulates cell glycolysis and proliferation in prostate cancer with expression levels elevated in cancerous over healthy cells, and notably, CRPC has shown a greater requirement for glucose metabolism compared to hormone-sensitive prostate cancer.^33,34^ We therefore selected hormone-resistant PC3 and hormone-sensitive LNCaP prostate cancer cell lines for comparative evaluation of glucose-gemcitabine conjugate **23** using a colorimetric MTS cell viability assay (Figure 3).^35^

In agreement with studies of other GLUT1-targeting prodrugs, we observed greater efficacy of glycoconjugate **23** against hormone-resistant PC3 over hormone-sensitive LNCaP cells, which have established lower levels of GLUT1.^36−38^ Notably, when **23** was incubated with PC3 cells in the presence of GLUT1 inhibitor Phloretin, we did observe a small decrease in cytotoxicity (see Figure S2).^13^ These preliminary results strengthen the hypothesis that glucoconjugation of therapeutics may be an effective approach to targeting cells that display increased uptake and metabolism of glucose and, in this example, may be worthy of more detailed biological study.

## Experimental Methods

### General Methods

All chemicals were purchased from Acros Organics, Alfa Aesar, Biosynth Carbosynth, Fisher Scientific, Fluorochem, Sigma-Aldrich, or TCI Chemicals and were used without further purification, unless otherwise stated. NMR spectra were recorded on a Bruker Avance 400 spectrometer. For reactions that required heating, DrySyn heating blocks were used as the heat source. The chemical shift data for each signal are given as δ in units of parts per million (ppm) relative to tetramethylsilane, where δ = 0.00 ppm. The number of protons (*n*) for a given resonance is indicated by *n*H. The multiplicity of each signal is indicated by s (singlet), bs (broad singlet), as (apparent singlet), ad (apparent doublet), d (doublet), t (triplet), q (quartet), p (pentet), sep (septet), dd (doublet of doublets), ddd (doublet of doublet of doublets), dddd (doublet of doublet of doublet of doublets), dt (doublet of triplets), tt (triplet of triplets), dqd (doublet of quartets of doublets), or m (multiplet). Coupling constants (*J*) are quoted in Hz and calculated to the nearest 0.1 Hz. Anhydrous DCM and pyridine were obtained from Sure/Seal bottles *via* chemical suppliers. Anhydrous THF, DCM, and toluene were obtained by passing the solvent through activated alumina columns, dispensed from a PureSolv MD ASNA solvent purification system, and stored over 4 Å molecular sieves. Unless otherwise stated, all reactions were conducted using anhydrous solvents under an atmosphere of N_2_, which was passed through a Drierite drying column. HRMS spectra were recorded on a ThermoScientific LTQ Orbitrap XL at the ESPRC National Mass Spectrometry Facility at Swansea University. Analytical thin layer chromatography (TLC) was carried out on precoated 0.25 mm Merck KgaA 60 F254 silica gel plates. Visualization was done by adsorption of UV light or thermal development after dipping in a methanolic solution of sulfuric acid (5% v/v). Automatic flash chromatography was carried out on silica gel (Reveleris X2 system) under a positive pressure of compressed N_2_. Optical rotations were recorded on a Bellingham + Stanley ADP430 (specific rotation, tube length: 50 mm, concentrations in g per 100 mL; RT, room temperature). The purity of final compounds was confirmed using an Agilent 1260 Infinity II preparative HPLC system equipped with a variable wavelength detector on a reverse phase column (Polaris 180 Å C18-A. 10 × 250 mm, 5 Μm), demonstrating a purity level >95%. Visualization was achieved using UV detection at 230 and 270 nm. Structural assignments were made with additional information from gCOSY and gHSQC experiments. Assignment of ^1^H and ^13^C atoms in NMR analysis follows the ring numbering systems shown in the Supporting Information.

### Synthesis and Characterization

#### Methyl 2,3,4-Tri-*O*-acetyl-6-deoxy-6-iodo-α-d-glucopyranoside **6**

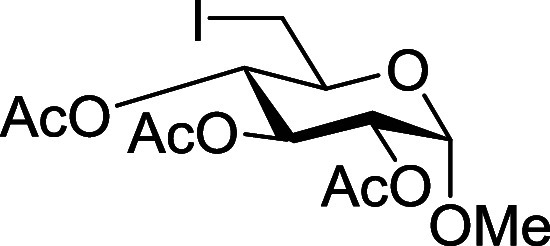



PPh_3_ (4.16 g, 15.9 mmol, 1.1 equiv) and imidazole (1.38 g, 20.2 mmol, 1.4 equiv) were added to a mixture of methyl-α-d-glucopyranoside **5** (3.00 g, 15.5 mmol, 1.0 equiv) in THF (150 mL) at RT. The reaction mixture was warmed to 70 °C, and then a solution of I_2_ (4.33 g, 17.1 mmol, 1.1 equiv) in THF (50 mL) was added dropwise over 3 h. After 2 h, completion of the reaction was confirmed by TLC (*R*_f_ = 0.25, EtOAc), the mixture was cooled to RT, and then pyridine (7.52 mL, 93.0 mmol, 6.0 equiv) and Ac_2_O (7.33 mL, 77.5 mmol, 5.0 equiv) were added to the mixture. The mixture was warmed to 35 °C and stirred for 17 h. After completion of the reaction as seen by TLC (*R*_f_ = 0.46, hexane/EtOAc, 7:3), EtOAc (100 mL) was added, and the organic layer was separated. The organic layer was washed with saturated aqueous Na_2_S_2_O_3_ (100 mL) and brine (100 mL). *i*-PrOH (100 mL) was added, and the mixture was concentrated. Precipitation was observed during the concentration, and further, *i*-PrOH (100 mL) was added to the slurry, cooled to 0 °C, and stirred for 1 h. The solid was filtered and washed with cold *i*-PrOH (100 mL) to afford the title compound as a white solid (4.71 g, 11.0 mmol, 71%). *R*_f_ = 0.46 (hexane/EtOAc, 7:3); ^1^H NMR (400 MHz, CDCl_3_) δ 5.48 (t, *J* = 10.0 Hz, 1H, H-3), 4.96 (d, *J* = 3.7 Hz, 1H, H-1), 4.88 (dd, *J* = 10.3, 3.7 Hz, 1H, H-2), 4.87 (t, *J* = 9.6 Hz, 1H, H-4), 3.83–3.76 (m, 1H, H-5), 3.48 (s, 3H, OMe), 3.30 (dd, *J* = 10.9, 2.5 Hz, 1H, H-6a), 3.14 (dd, *J* = 10.9, 8.3 Hz, 1H, H-6b), 2.08 (s, 3H, OAc), 2.06 (s, 3H, OAc), 2.01 (s, 3H, OAc); ^13^C{^1^H} NMR (101 MHz, CDCl_3_) δ 170.1 (C=O, Ac), 170.0 (C=O, Ac), 169.7 (C=O, Ac), 96.7 (C1), 72.5 (C4), 70.9 (C2), 69.7 (C3), 68.6 (C5), 55.8 (OCH_3_), 20.7 (Ac-CH_3_), 20.7 (Ac-CH_3_), 20.7 (Ac-CH_3_), 3.6 (C6); HRMS *m*/*z* (ES^+^) found: (M + NH_4_)^+^ 448.0469; C_13_H_23_NO_8_I requires M^+^ 448.0463. Data matched those reported previously.^39^

#### Methyl 6-*S*-Acetyl-6-deoxy-6-thio-2,3,4-tri-*O*-acetyl-α-d-glucopyranoside **7**

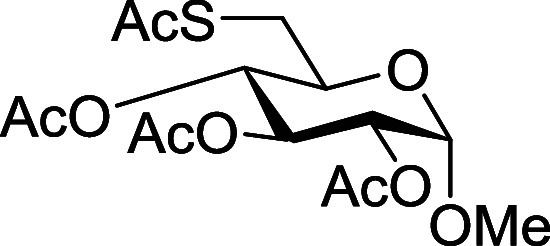



Methyl 2,3,4-tri-*O*-acetyl-6-deoxy-6-iodo-α-d-glucopyranoside **6** (2.00 g, 4.65 mmol, 1.0 equiv) and KSAc (1.27 g, 11.2 mmol, 2.4 equiv) in acetone (100 mL) were heated to reflux for 1 h. Upon reaction completion as seen by TLC (*R*_f_ = 0.36, hexane/EtOAc, 7:3), the mixture was filtered, and the filtrate was concentrated under reduced pressure. The crude residue was dissolved in EtOAc (100 mL), washed with water (100 mL) and brine (100 mL), dried (MgSO_4_), and filtered. The combined organic phases were evaporated under reduced pressure and purified by column chromatography (hexane/EtOAc, 0–50%), yielding the title compound (1.23 g, 3.25 mmol, 70%) as a white solid. *R*_f_ = 0.33 (hexane/EtOAc, 7:3); ^1^H NMR (400 MHz, CDCl_3_) δ 5.43 (t, *J* = 9.7 Hz, 1H, H-3), 4.94 (t, *J* = 9.6 Hz, 1H, H-4), 4.89 (d, *J* = 3.7 Hz, 1H, H-1), 4.86 (dd, *J* = 10.1, 3.7 Hz, 1H, H-2), 3.92 (ddd, *J* = 10.0, 7.0, 3.0 Hz, 1H, H-5), 3.40 (s, 3H, OMe), 3.21 (dd, *J* = 14.2, 3.0 Hz, 1H, H-6a), 3.07 (dd, *J* = 14.2, 7.0 Hz, 1H, H-6b), 2.35 (s, 3H, SAc), 2.08 (s, 3H, OAc), 2.07 (s, 3H, OAc), 2.00 (s, 3H, OAc); ^13^C{^1^H} NMR (101 MHz, CDCl_3_) δ 194.6 (C=O, SAc), 170.1 (C=O, Ac), 170.0 (C=O, Ac), 169.9 (C=O, Ac), 96.6 (C1), 70.94 (C4), 70.88 (C2), 70.0 (C3), 68.2 (C5), 55.4 (OCH_3_), 30.4 (Ac-CH_3_), 30.0 (C6), 20.72 (2 × Ac-CH_3_), 20.68 (Ac-CH_3_); HRMS *m*/*z* (ES^+^) found: (M + NH_4_)^+^ 396.1320; C_15_H_26_NO_9_S requires M^+^ 396.1323. Data matched those reported previously.^40^

#### Bis(methoxy 2,3,4-tri-*O*-acetyl-6-thio-α-d-glucopyranoside)-6,6′-disulfide **8**

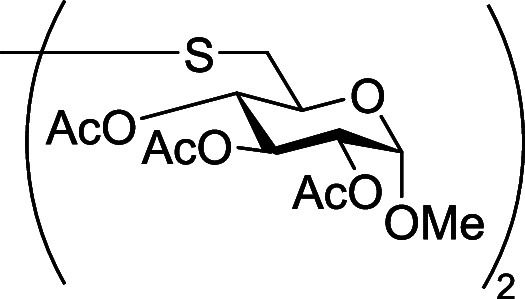



To a solution of **7** (487 mg, 1.29 mmol, 1.0 equiv) in MeCN (10 mL) were added I_2_ (819 mg, 3.22 mmol, 2.5 equiv) and NIS (145 mg, 0.65 mmol, 0.5 equiv). The reaction mixture was stirred at RT for 2 h. When TLC indicated full conversion of the starting material (*R*_f_ = 0.29, hexane/EtOAc, 7:3), the resulting mixture was diluted with water (50 mL) and then extracted with DCM (50 mL). The combined organic phases were washed with saturated aqueous Na_2_S_2_O_3_ (50 mL) and brine (50 mL), dried (MgSO_4_), and filtered. After the removal of the solvent under reduced pressure, the crude residue was purified by column chromatography, affording the title compound as a colorless oil (330 mg, 0.98 mmol, 77%). *R*_f_ = 0.29 (hexane/EtOAc, 7:3); ^1^H NMR (400 MHz, CDCl_3_) δ 5.46 (dd, *J* = 10.1, 9.4 Hz, 1H, H-3), 4.95–4.78 (m, 3H, H-1, H-2, H-4), 4.02 (td, *J* = 9.9, 2.8 Hz, 1H, H-5), 3.44 (s, 3H, OMe), 2.92 (dd, *J* = 13.8, 2.9 Hz, 1H, H-6a), 2.84 (dd, *J* = 13.8, 8.7 Hz, 1H, H-6b), 2.07 (s, 3H, OAc), 2.06 (s, 3H, OAc), 2.01 (s, 3H, OAc); ^13^C{^1^H} NMR (101 MHz, CDCl_3_) δ 168.3 (C=O, Ac), 168.1 (C=O, Ac), 168.0 (C=O, Ac), 94.7 (C1), 69.9 (C2), 69.0 (C4), 68.1 (C3), 65.6 (C5), 53.7 (OCH_3_), 39.6 (C6), 18.83 (Ac-CH_3_), 18.80 (Ac-CH_3_); HRMS *m*/*z* (ES^+^) found: (M + NH_4_)^+^ 688.1940; C_26_H_42_NO_16_S_2_ requires M^+^ 688.1940. Data matched those previously reported.^41^

#### Bis(1,2,3,4-tetra-*O*-acetyl-6-thio-α/β-d-glucopyranoside)-6,6′-disulfide **9**

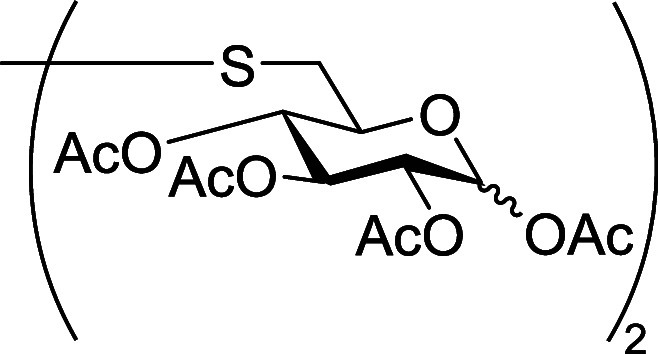



A solution of disulfide **8** (369 mg, 1.10 mmol, 1.0 equiv) was dissolved in Ac_2_O (5 mL) and H_2_SO_4_ (100 μL). The resulting mixture was stirred overnight at RT, and upon reaction completion as seen by TLC (*R*_f_ = 0.19 (hexane/EtOAc, 6:4), the mixture was diluted with CHCl_3_ (100 mL) and washed with water (50 mL), saturated aqueous NaHCO_3_ (3 × 50 mL), and brine (50 mL). The combined organic phases were dried (MgSO_4_), filtered, and concentrated under reduced pressure. The crude residue was purified by column chromatography (hexane/EtOAc, 0–40%), yielding the title compound (272 mg, 0.75 mmol, 68%, α/β, 1:0.25) as a yellow oil. *R*_f_ = 0.19 (hexane/EtOAc, 6:4); ^1^H NMR (400 MHz, CDCl_3_) δ 6.31 (d, *J* = 3.7 Hz, 1H, H-1α), 5.71 (d, *J =* 8.28 Hz, 1H, H-1β), 5.46 (t, *J* = 9.8 Hz, 1H, H-3α), 5.13–4.97 (m, 2H, H-2α, H-4α), 4.19–4.12 (m, 1H, H-5α), 2.94 (dd, *J* = 14.1, 3.4 Hz, 1H, H-6bα), 2.82 (dd, *J* = 14.1, 7.5 Hz, 1H, H-6aα), 2.18 (s, 3H, OAc), 2.07 (s, 3H, OAc), 2.02 (s, 3H, OAc), 2.01 (s, 3H, OAc); ^13^C{^1^H} NMR (101 MHz, CDCl_3_) δ 170.2 (C=O, Ac), 169.67 (C=O, Ac), 169.66, 168.7 (C=O, Ac), 91.6 (C1β), 88.9 (C1α), 71.1 (C4α), 70.2 (C5α), 69.7 (C3α), 69.3 (C2α), 41.3 (C6α), 20.9 (Ac-CH_3_), 20.70 (Ac-CH_3_), 20.66 (Ac-CH_3_), 20.5 (Ac-CH_3_); HRMS *m*/*z* (ES^+^) found: (M + H)^+^ 726.1489; C_28_H_39_O_18_S_2_ requires M^+^ 726.1499.

#### 1,2,3,4-Tetra-*O*-acetyl-6-deoxy-*S*-mercaptopropionic-acid-6-thio-α/β-d-glucopyranoside **10**

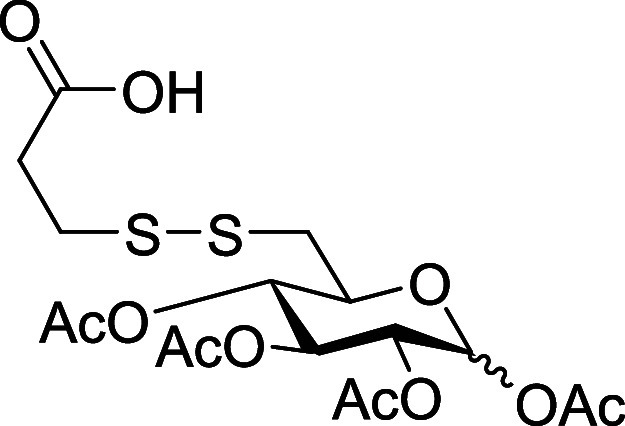



To a solution of disulfide **9** (493 mg, 1.36 mmol, 1.0 equiv) in MeCN (5 mL) was added DTT (230 mg, 1.49 mmol, 1.1 equiv), and the mixture was stirred overnight at RT. After evaporation of the solvent under reduced pressure, THF (5 mL) and NaH (33.0 mg, 1.36 mmol, 1.0 equiv) were added and the mixture was cooled to −20 °C. A solution of TCCA (316 mg, 1.36 mmol, 1.0 equiv) in MeCN (5 mL) was added, followed by quick addition of 3-mercaptopropionic acid (0.18 mL, 2.04 mmol, 1.5 equiv). The reaction mixture was kept stirring for 20 min at −20 °C; at this point, TLC revealed reaction completion (*R*_f_ = 1.0, EtOAc) and the solvent was removed under reduced pressure. The crude residue was purified directly by column chromatography (hexane/EtOAc, 0–100%) to deliver the title compound as a colorless oil (326 mg, 0.70 mmol, 51%, 4:1, α/β). *R*_f_ = 1.0 (EtOAc); ^1^H NMR (400 MHz, CDCl_3_) δ 6.32 (d, *J* = 3.7 Hz, 1H, αH-1), 5.73 (d, *J* = 8.2 Hz, 1H, βH-1), 5.50–5.43 (t, *J* = 9.8 Hz, 1H, H-3), 5.07 (dd, *J* = 10.3, 3.7 Hz, 1H, H-2), 5.01 (at, *J* = 10.0 Hz 1H, H-4), 4.17 (ddd, *J* = 10.4, 7.8, 2.8 Hz, 1H, H-5), 2.99–2.88 (m, 3H, H-6a, CH_2_), 2.86–2.72 (m, 3H, H-6b, CH_2_), 2.18 (s, 3H, OAc), 2.07 (s, 3H, OAc), 2.03 (s, 3H, OAc), 2.02 (s, 3H, OAc); ^13^C{^1^H} NMR (101 MHz, CDCl_3_) δ 176.9 (C=O), 170.3 (C=O, Ac), 169.74 (C=O, Ac), 169.73 (C=O, Ac), 168.9 (C=O, Ac), 91.7 (C1β), 88.9 (C1α), 71.1 (C4), 70.2 (C5), 69.8 (C3), 69.3 (C2), 41.3 (C6), 33.7 (CH_2_), 32.8 (CH_2_), 20.9 (Ac-CH_3_), 20.7 (Ac-CH_3_), 20.7 (Ac-CH_3_), 20.5 (Ac-CH_3_); HRMS *m*/*z* (ES^–^) found: (M – H)^−^ 467.0694; C_17_H_23_NO_11_S_2_ requires M^–^ 467.0687.

#### 3′,5′-Di-*O*-terbutyldimethylsilyl-2′-deoxy-2′-gem-difluoro-1′-(β-d-ribofuranosyl) cytosine **11**

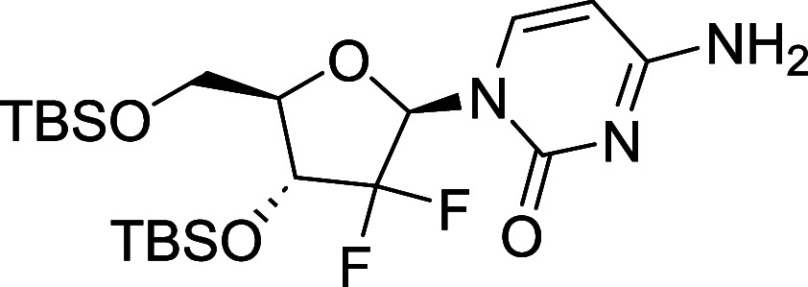



A solution of gemcitabine (200 mg, 0.76 mmol, 1.0 equiv) in DMF (20.0 mL) was treated with imidazole (155 mg, 2.28 mmol, 3.0 equiv) and TBDMSCl (0.59 mL, 627 mg, 2.28 mmol, 3.0 equiv). After stirring at RT for 24 h, TLC analysis revealed reaction completion (*R*_f_ = 0.57, DCM/MeOH, 9:1). The resulting mixture was diluted with water (50 mL) and then extracted with DCM (50 mL). The combined organic phases were dried (MgSO_4_) and filtered, and the solvent was removed under reduced pressure. The crude residue was purified by column chromatography (DCM/MeOH, 0–10%) to yield the title compound (322 mg, 0.66 mmol, 86%) as a crystalline white solid. *R*_f_ = 0.57 (DCM/MeOH, 9:1); ^1^H NMR (400 MHz, CDCl_3_) δ 7.50 (d, *J* = 7.5 Hz, 1H, CH), 6.20 (dd, ^3^*J*_H-1′-Fa/Fb_ = 10.8, 4.5 Hz, H-1′), 5.70 (d, *J* = 7.5 Hz, 1H, CH), 4.19 (td, *J* = 11.5, 8.1 Hz, 1H, H-3′), 3.87 (d, *J* = 11.7 Hz, 1H, H-5a′), 3.76 (ad, *J* = 8.0 Hz, 1H, H-4′), 3.68 (dd, *J* = 11.8, 2.1 Hz, 1H, H-5b′), 0.82 (s, 9H, Si-^*t*^Bu), 0.79 (s, 9H, Si-^*t*^Bu), 0.02–0.01 (m, 12H, Si-CH_3_); ^13^C{^1^H} NMR (101 MHz, CDCl_3_) δ 166.0 (C-NH_2_, C4), 155.7 (C=O, C2), 140.4 (C6), 122.1 (d, ^1^*J*_C–F_ = 259.2 Hz), 120.79 (d, ^1^*J*_C–F_ = 260.7 Hz, C2′), 95.4 (C5), 84.2 (dd, ^2^*J*_C–F_ = 40.6, 23.2 Hz, C1′), 80.9 (d, ^3^*J*_C–F_ = 8.8 Hz, C4′), 69.8 (dd, ^2^*J*_C–F_ = 18.2 Hz, C3′), 60.1 (C5′, 25.8 (Si-^*t*^Bu), 25.5 (Si-^*t*^Bu), 18.3 (Si-^*t*^Bu), 18.0 (Si-^*t*^Bu), −4.8 (Si-CH_3_), −5.3 (Si-CH_3_), −5.46 (Si-CH_3_), −5.51 (Si-CH_3_); ^19^F NMR (377 MHz, CDCl_3_) δ −115.92 (dd, *J* = 238.0, 11.7 Hz), −117.52 (dt, *J* = 238.7, 10.6 Hz); HRMS *m*/*z* (ES^+^) found: (M + Na)^+^ 514.2336; C_21_H_39_N_3_O_4_F_2_Si_2_Na requires M^+^ 514.2339. Data matched those previously reported.^42^

#### Protected Glucose-Gemcitabine Conjugate **12**

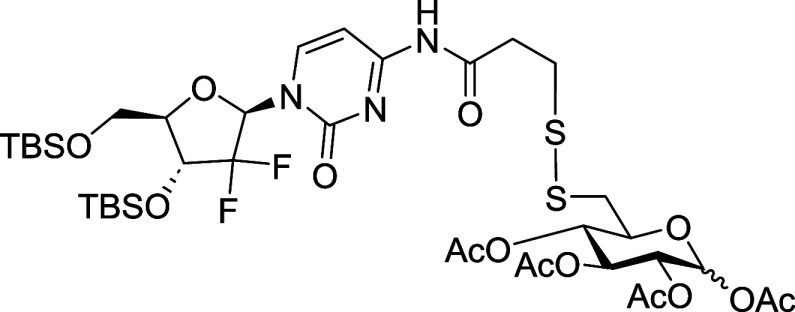



To a solution of disulfide **10** (105 mg, 0.22 mmol, 1.0 equiv) in DCM (10 mL) was added EDC (104 mg, 0.67 mmol, 3.0 equiv) followed by DMAP (2.70 mg, 22.4 μmmol, 0.1 equiv). The reaction mixture was stirred at RT for 15 min. Protected gemcitabine **11** (220 mg, 0.45 mmol, 2.0 equiv) was next added, and the reaction mixture was stirred for a further 45 min. TLC analysis revealed reaction completion (*R*_f_ = 0.86, EtOAc/hexane, 7:3). The reaction mixture was diluted with DCM (50 mL) and washed with saturated aqueous NaHCO_3_ (2 × 50 mL). The combined organic phases were dried (MgSO_4_), filtered, and concentrated under reduced pressure. Purification by column chromatography (hexane/EtOAc, 0–30%) delivered the title compound (139 mg, 0.15 mmol, 65%, 1:0.15, α/β) as a colorless oil. *R*_f_ = 0.86 (EtOAc/hexane, 7:3); ^1^H NMR (400 MHz, CDCl_3_) δ 9.14 (s, 1H, NH′), 8.07 (d, *J* = 7.5 Hz, 1H, CH′), 7.40 (d, *J* = 7.1 Hz, 1H, CH′), 6.37–6.28 (m, 2H, H-1″, H-1α), 5.68 (d, *J* = 8.3 Hz, 1H, H-1β), 5.47 (t, *J* = 9.8 Hz, 1H, H-3α), 5.08 (dd, *J* = 10.3, 3.7 Hz, 1H, H-2α), 5.02 (t, *J* = 9.7 Hz, 1H, H-4α), 4.34 (td, *J* = 11.6, 8.2 Hz, 1H, H-3″), 4.16–4.10 (m, 1H, H-5α), 4.02 (d, *J* = 11.8 Hz, 1H, H-5a″), 3.96 (d, *J* = 8.0 Hz, 1H, H-4″), 3.81 (dd, *J* = 11.9, 1.8 Hz, 1H, H-5b″), 3.06–2.87 (m, 5H, H-6aα, 2 × CH_*2*_), 2.78 (dd, *J* = 14.2, 7.7 Hz, 1H, H-6bα), 2.20 (s, 3H, OAc), 2.08 (s, 3H, OAc), 2.03 (s, 3H, OAc), 2.02 (s, 3H, OAc), 0.95 (s, 9H, Si-^*t*^Bu), 0.91 (s, 9H, Si-^*t*^Bu), 0.13 (s, 3H, Si-CH_3_), 0.13 (s, 3H, Si-CH_3_), 0.13 (s, 3H, Si-CH_3_), 0.10 (s, 3, Si-CH_3_); ^13^C{^1^H} NMR (101 MHz, CDCl_3_): 170.2, 169.8 (C=O), 169.6 (C=O), 169.1 (C=O), 144.4 (CH′), 96.8 (CH′), 91.8 (C1β), 88.9 (C1α), 84.7 (C1″), 71.0 (C4α), 70.2 (C5α), 69.7 (C3α), 69.3 (C2α), 60.0 (C5″), 41.5 (C6α), 36.9 (CH_2_), 32.5 (CH_2_), 25.9 (Si-^*t*^Bu″), 25.5 (Si-^*t*^Bu″), 20.9 (Ac-CH_3_), 20.72 (Ac-CH_3_), 20.68 (Ac-CH_3_), 20.5 (Ac-CH_3_), 18.3 (Si-^*t*^Bu″), 18.0 (Si-^*t*^Bu″), −4.8 (Si-CH_3_), −5.3 (Si-CH_3_), −5.4 (Si-CH_3_), −5.5 (Si-CH_3_).

#### 4-(2-Pyridyldithio)butanoic Acid **15**

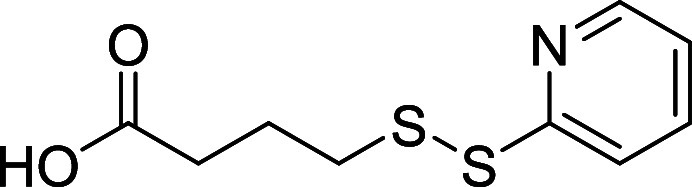



To a solution of bis(2-pyridinyl) disulfide **S1** (1.83 g, 8.31 mmol, 2.0 equiv) in MeOH was added 4-mercaptobutyric acid (0.43 mL, 4.16 mmol, 1.0 equiv), and the resulting mixture was stirred at RT for 2 h. TLC analysis revealed complete consumption of the starting material (*R*_f_ = 0.63, DCM/MeOH, 9:1), and the solvent was removed under reduced pressure. The crude residue was directly purified by column chromatography (DCM/MeOH, 0–10%) to deliver the title compound (649 mg, 2.83 mmol, 68%) as a colorless oil. *R*_f_ = 0.63 (DCM/MeOH, 9:1); ^1^H NMR (400 MHz, CDCl_3_) δ 10.22 (bs, 1H, COOH), 8.48 (ddd, *J* = 4.9, 1.8, 0.9 Hz, 1H, ArH), 7.74 (dt, *J* = 8.1, 1.0 Hz, 1H, ArH), 7.70–7.64 (m, 1H, ArH), 7.11 (ddd, *J* = 7.3, 4.9, 1.1 Hz, 1H, ArH), 2.86 (t, *J* = 7.1 Hz, 2H, CH_2_), 2.50 (t, *J* = 7.2 Hz, 2H, CH_2_), 2.04 (p, *J* = 7.2 Hz, 2H, CH_2_); ^13^C{^1^H} NMR (101 MHz, CDCl_3_) δ 177.7 (C=O), 160.0 (Ar–C), 149.3 (Ar–C), 137.5 (Ar–C), 120.9 (Ar–C), 120.0 (Ar–C), 37.8 (CH_2_), 32.4 (CH_2_), 23.8 (CH_2_); HRMS *m*/*z* (ES^+^) found: (M + H)^+^ 230.0303; C_9_H_12_O_2_NS_2_ requires M^+^ 230.0304. Data matched those previously reported.^43^

#### 4-*N-*(2-Pyridyl-disulfanyl-butylcarbonylamino)-3′,5′-di-*O*-terbutyldimethylsilyl-2′-deoxy-2′-gem-difluoro-1′-(β-d-ribofuranosyl)cytosine **16**

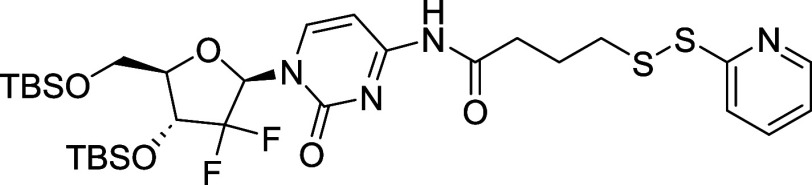



To a solution of 4-(2-pyridyldithio) butanoic acid **15** (113 mg, 0.49 mmol, 2.0 equiv) in DCM (10 mL) was added EDC (115 mg, 0.73 mmol, 3.0 equiv) followed by DMAP (3.00 mg, 24.6 μmmol, 0.1 equiv). The reaction mixture was stirred at RT for 15 min, protected gemcitabine **11** (121 mg, 0.25 mmol, 1.0 equiv) was added, and the reaction mixture was stirred for a further 1 h. TLC analysis revealed reaction completion (*R*_f_ = 0.66, DCM/MeOH, 9:1), and the reaction mixture was diluted with DCM (50 mL) and washed with saturated aqueous NaHCO_3_ (2 × 50 mL). The combined organic phases were dried (MgSO_4_), filtered, and concentrated under reduced pressure. Purification by column chromatography (hexane/EtOAc, 0–30%) delivered the title compound (139 mg, 0.20 mmol, 80%) as a colorless oil. *R*_f_ = 0.66 (DCM/MeOH, 9:1); [α]_D_^23^ = +26.8 (*c* = 1.0, CHCl_3_); ^1^H NMR (400 MHz, CDCl_3_) δ 9.32 (bs, 1H, NH), 8.34–8.32 (m, 1H, ArH), 7.94 (d, *J* = 7.6 Hz, 1H, CH), 7.58–7.44 (m, 2H, ArH), 7.27 (d, *J* = 7.4 Hz, 1H, CH), 6.94 (ddd, *J* = 7.1, 4.8, 1.2 Hz, 1H, ArH), 6.18 (dd, ^3^*J*_H-1′-Fa/Fb_ = 10.2, 3.6 Hz, 1H, H1′), 4.20 (td, *J* = 11.6, 8.2 Hz, 1H, H-3′), 3.89 (d, *J* = 11.9 Hz, 1H, H-5a′), 3.82 (ad, *J* = 8.0 Hz, 1H, H-4′), 3.68 (dd, *J* = 11.9, 1.7 Hz, 1H, H-5b′), 2.74 (t, *J* = 7.0 Hz, 2H, CH_2_), 2.54 (t, *J* = 7.2 Hz, 2H, CH_2_), 1.97 (p, *J* = 7.1 Hz, 2H, CH_2_), 0.82 (s, 9H, Si-^*t*^Bu), 0.77 (s, 9H, Si-^*t*^Bu), 0.05 to −0.05 (m, 12H, Si-CH_3_); ^13^C{^1^H} NMR (101 MHz, CDCl_3_) δ 163.0 (C-NH, C4), 160.1 (C=O), 154.9 (C=O, C2), 149.7 (Ar–C), 144.0 (C6′), 137.0 (Ar–C), 120.7 (Ar–C), 119.9 (Ar–C), 97.0 (C5′), 84.7 (dd, ^2^*J*_C–F_ = 40.7, 23.7 Hz, C1′), 81.5 (d, ^3^*J*_C–F_ = 8.8 Hz, C4′), 69.4 (dd, ^2^*J*_C–F_ = 26.8, 18.7 Hz, C3′), 59.9 (C5′), 37.7 (CH_2_), 35.6 (CH_2_), 25.9 (Si-^*t*^Bu), 25.5 (Si-^*t*^Bu), 23.7 (CH_2_), 18.3 (Si-^*t*^Bu), 18.0 (Si-^*t*^Bu), −4.8 (Si-CH_3_), −5.3 (Si-CH_3_), −5.4 (Si-CH_3_), −5.5 (Si-CH_3_); ^19^F NMR (377 MHz, CDCl_3_) δ −115.98 (dd, *J* = 239.1, 12.2 Hz), −117.35 (dt, *J* = 239.2, 10.6 Hz); HRMS *m*/*z* (ES^+^) found: (M + H)^+^ 703.2644; C_30_H_49_O_5_N_4_F_2_S_2_Si_2_ requires M^+^ 703.2645.

#### 4-*N-*(2-Pyridyl-disulfanyl-butylcarbonylamino)-2′-deoxy-2′-gem-difluoro-1′-(β-d-ribofuranosyl)cytosine **17**

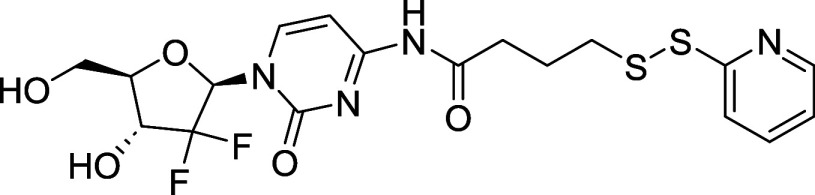



To a solution of disulfide **16** (70 mg, 100 μmol, 1.0 equiv) in THF (2 mL) was added 1 M TBAF in THF (0.60 mL, 0.60 mmol, 3.0 equiv). After 2 h of stirring, TLC analysis revealed reaction completion (*R*_f_ = 0.33, DCM/MeOH, 9:1) and the mixture was diluted with DCM and washed with saturated aqueous NaHCO_3_ (2 × 5 mL). The combined organic phases were dried (MgSO_4_), filtered, and concentrated under reduced pressure. Purification by column chromatography (DCM/MeOH, 0–20%) delivered the title compound (31.0 mg, 65.3 μmmol, 66%) as colorless oil. *R*_f_ = 0.33 (DCM/MeOH, 9:1); [α]_D_^19^ = +19.5 (*c* = 0.5, CHCl_3_); ^1^H NMR (400 MHz, MeOD) δ 8.41–8.34 (m, 1H, ArH), 7.90–7.76 (m, 3H, ArH, CH), 7.35–7.18 (m, 1H, ArH), 6.27 (at, *J* = 8.7 Hz, 1H, H1′), 5.93 (d, *J* = 7.6 Hz, 1H, CH), 5.44–5.35 (m, 1H, H3′), 4.15–4.11 (m, 1H, H4′), 3.89 (dd, *J* = 12.8, 2.6 Hz, 1H, H-5a′), 3.73 (dd, *J* = 12.8, 3.4 Hz, 1H, H-5b′), 2.88 (t, *J* = 7.1 Hz, 2H, CH_2_), 2.62 (t, *J* = 7.2 Hz, 2H, CH_2_), 2.07–2.00 (m, 2H, CH_2_); ^13^C{^1^H} NMR (101 MHz, MeOD) δ 171.3 (C-NH, C4), 166.3 (C=O), 159.9 (C=O), 149.0 (CH), 137.8 (Ar–C), 121.0 (Ar–C), 119.9 (Ar–C), 95.1 (CH), 84.4 (C1′), 79.4 (d, ^3^*J* = 7.1 Hz, C4′), 69.4 (C2′), 59.3 (C5′), 37.2 (CH_2_), 31.3 (CH_2_), 23.6 (CH_2_); ^19^F NMR (377 MHz, MeOD) δ −111.22 to −123.14 (m).

#### 2,3,4-Tri-*O*-acetyl-6-*S*-acetyl-6-deoxy-6-thio-α/β-d-glucopyranoside **18**

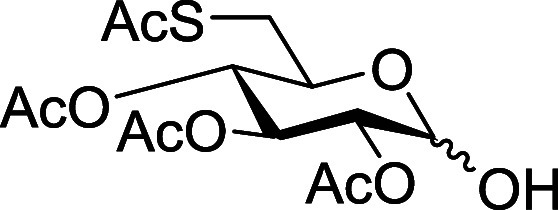



A solution of **7** (1.90 g, 5.03 mmol, 1.0 equiv) in AcOH (5 mL) and H_2_SO_4_ (10.0 μL) was stirred for 3 h at RT. Upon reaction completion by TLC (*R*_f_ = 0.15, hexane/EtOAc, 1:1), the mixture was diluted with DCM (100 mL) and washed with water (50 mL), saturated aqueous NaHCO_3_ (3 × 50 mL), and brine (50 mL). The combined organic phases were dried (MgSO_4_), filtered, and concentrated under reduced pressure. The crude residue was purified by column chromatography (hexane/EtOAc, 0–60%) to yield the title compound (1.13 g, 3.11 mmol, 62%, 1:0.15, α/β) as a colorless oil. *R*_f_ = 0.15 (hexane/EtOAc, 1:1); ^1^H NMR (400 MHz, CDCl_3_) δ 6.31 (d, *J* = 3.7 Hz, 1H, H-1α), 5.71 (d, *J* = 8.3 Hz, 1H, H-1β), 5.46 (t, *J* = 9.7 Hz, 1H, H-3α), 5.07 (dd, *J* = 10.3, 3.8 Hz, 1H, H-2α), 5.01 (t, *J* = 9.7 Hz, 1H, H-4α), 4.19–4.13 (m, 1H, H-5α), 2.94 (dd, *J* = 14.1, 3.4 Hz, 1H, H-6aα), 2.82 (dd, *J* = 14.1, 7.4 Hz, 1H, H-6bα), 2.18 (s, 3H, OAc), 2.07 (s, 3H, OAc), 2.02 (s, 3H, OAc), 2.01 (s, 3H, OAc); ^13^C{^1^H} NMR (101 MHz, CDCl_3_) δ 170.2 (C=O, Ac), 169.7 (C=O, Ac), 168.7 (C=O, Ac), 91.6 (C1β), 88.9 (C1α), 71.1 (C4α), 70.2 (C5α), 69.8 (C3α), 69.3 (C2α), 41.3 (C6α), 20.8 (Ac-CH_3_), 20.69 (Ac-CH_3_), 20.65 (Ac-CH_3_), 20.5 (Ac-CH_3_); HRMS *m*/*z* (ES^+^) found: (M + NH_4_)^+^ 382.1174; C_14_H_24_O_9_SN requires M^+^ 382.1171.

#### Bis(6-thio-α/β-d-glucopyranoside)-6,6′-disulfide **20**

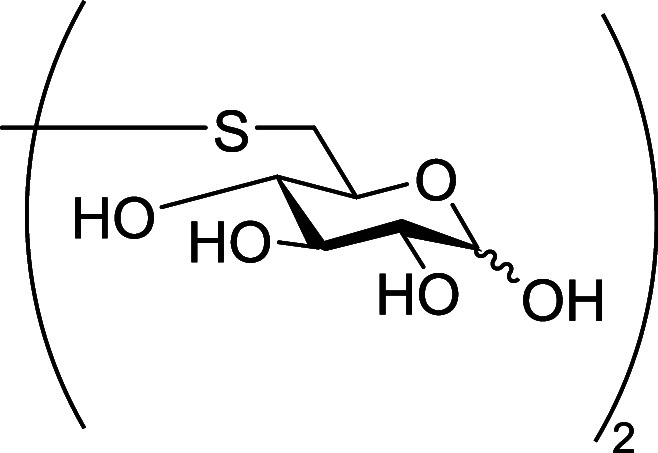



A solution of hemi-acetal **18** (186 mg, 0.51 mmol, 1.0 equiv) in MeOH (5 mL) was treated with Na (1.20 mg, 51.0 μmmol, 0.1 equiv). The reaction mixture was left stirring at RT for 30 min, and TLC analysis revealed reaction completion (*R*_f_ = 0.16, DCM/MeOH, 7:3). Amberlite IR120 (H^+^) ion-exchange resin was added until the pH of the reaction mixture was neutral. The mixture was filtered and washed with methanol (25 mL), the filtrate was concentrated under reduced pressure, and the crude residue was purified by column chromatography (DCM/MeOH, 0–30%), yielding the title compound (84.7 mg, 0.43 mmol, 85%, 1:1, α/β) as a colorless oil. *R*_f_ = 0.16 (DCM/MeOH, 7:3); ^1^H NMR (400 MHz, MeOD) δ 5.08 (d, *J* = 3.4 Hz, 1H, H-1α), 4.48 (add, *J* = 7.8, 2.1 Hz, 1H, H-1β), 4.01 (td, *J* = 10.5, 2.1 Hz, 1H), 3.65 (t, *J* = 9.3 Hz, 1H), 3.56–3.46 (m, 5H), 3.23–3.12 (m, 3H), 2.86–2.71 (m, 2H); ^13^C{^1^H} NMR (101 MHz, MeOD) δ 96.85 (C1β), 96.84 (C1β), 92.5 (C1α), 76.51, 76.49, 74.9, 74.60, 74.2, 73.6, 73.5, 73.4, 73.3, 73.2, 72.5, 70.0, 69.7, 48.5, 42.2, 42.0, 41.9, 41.5; HRMS *m*/*z* (ES^–^) found: (M – H)^−^ 389.0588; C_12_H_21_O_10_S_2_ requires M^–^ 389.0582.

#### Gemcitabine Disulfide **21**

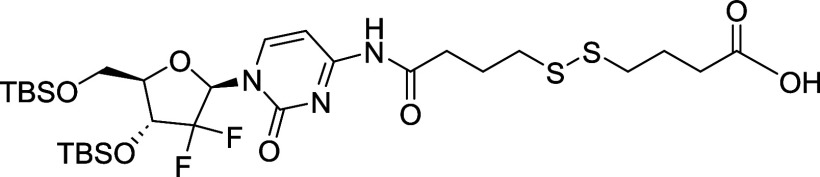



To a solution of gemcitabine conjugate **17** (254 mg, 0.36 mmol, 1.0 equiv) in MeCN was added 4-(2-pyridyldithio)butanoic acid **15** (75.0 μL, 0.72 mmol, 2.0 equiv), and the reaction mixture was stirred at RT for 2 h. TLC analysis revealed reaction completion (*R*_f_ = 0.47, EtOAc/DCM, 7:3), the solvent was removed under reduced pressure, and the crude residue was purified by column chromatography (DCM/EtOAc, 0–40%) to yield the title compound (156 mg, 0.22 mmol, 61%) as a colorless oil. *R*_f_ = 0.47 (EtOAc/DCM, 7:3); [α]_D_^23^ = +16.3 (*c* = 1.0, CHCl_3_); ^1^H NMR (400 MHz, CDCl_3_) δ 7.97 (d, *J* = 7.6 Hz, 1H, CH), 7.32 (d, *J* = 7.6 Hz, 1H, CH), 6.18 (dd, *J*_H-1′-Fa/Fb_ = 10.0, 3.8 Hz, 1H, H-1′), 4.20 (td, *J* = 11.6, 8.1 Hz, 1H, H-3′), 3.89 (d, *J* = 11.9 Hz, 1H, H-5a′), 3.83 (d, *J* = 8.0 Hz, 1H, H-4′), 3.68 (dd, *J* = 11.9, 1.8 Hz, 1H, H-5b′), 2.72 (t, *J* = 6.4 Hz, 2H, CH_2_), 2.61–2.46 (m, 4H, 2 × CH_2_), 2.41–2.34 (m, 2H, CH_2_), 2.03–1.90 (m, 4H, 2 × CH_2_), 0.82 (s, 9H, Si-^*t*^Bu), 0.77 (s, 9H, Si-^*t*^Bu), 0.00 (s, 3H, Si-CH_3_), −0.00 (s, 3H, Si-CH_3_), −0.01 (s, 3H, Si-CH_3_), −0.03 (s, 3H, Si-CH_3_); ^13^C{^1^H} NMR (101 MHz, CDCl_3_) δ 173.6 (C-NH, C4), 163.3 (C=O), 154.15 (C=O), 154.17 (C=O), 144.8 (C6), 123.2 (d, ^1^*J*_C–F_ = 261.2 Hz, d, ^1^*J*_C–F_ = 260.7 Hz, C2′), 96.8 (C5), 84.7 (dd, ^2^*J*_C–F_ = 41.1, 23.7 Hz, C1′), 81.6 (d, ^3^*J*_C–F_ = 8.7 Hz, C4′), 69.5 (d, ^2^*J*_C–F_ = 17.8 Hz, C3′), 60.0 (C5′), 40.6 (CH_2_), 38.0 (CH_2_), 36.7 (CH_2_), 32.5 (CH_2_), 25.9 (Si-^*t*^Bu), 25.8 (CH_2_), 25.5 (Si-^*t*^Bu), 24.7 (CH_2_), 18.3 (Si-^*t*^Bu), 18.0 (Si-^*t*^Bu), −4.8 (Si-CH_3_), −5.3 (Si-CH_3_), −5.4 (Si-CH_3_), −5.5 (Si-CH_3_); ^19^F NMR (377 MHz, CDCl_3_) δ −115.94 (dd, *J* = 239.5, 11.3 Hz), −117.56 (ad, *J* = 239.7 Hz); HRMS *m*/*z* (ES^–^) found: (M – H)^−^ 710.2610; C_29_H_50_O_7_ N_3_F_3_S_2_Si_2_ requires M^–^ 710.2602.

#### 1,2,3,4-Tetra-*O*-trimethylsilyl-α-d-glucopyranoside **22**

To a solution of d-glucose (360 mg, 2.00 mmol, 1.0 equiv) in pyridine (20 mL) were added HMDS (0.94 mL, 4.50 mmol, 2.25 equiv) and TMSCl (1.78 mL, 14.8 mmol, 7.4 equiv) at 0 °C. The reaction was allowed to reach RT and stirred for 1 h, and TLC analysis revealed reaction completion (*R*_f_ = 0.90, hexane). The reaction mixture was diluted with DCM (50 mL) and washed with H_2_O (2 × 50 mL). The combined organic phases were dried (MgSO_4_) and filtered, and the solvent was removed under reduced pressure. Crude 1,2,3,4,6-penta-*O*-trimethylsilyl-α-d-glucopyranoside was obtained as a colorless oil and progressed to the next step without further purification. To 1,2,3,4,6-penta-*O*-trimethylsilyl-α-d-glucopyranoside in DCM (15 mL) at 0 °C was added AcOH (65.0 μL) in MeOH (12 mL) dropwise. The reaction was maintained at 0 °C for 1 h; at this point, TLC analysis revealed reaction completion (*R*_f_ = 0.17, hexane). The reaction mixture was diluted with DCM (50 mL) and washed with H_2_O (2 × 50 mL), the combined organic phases were dried (MgSO_4_) and filtered, and the solvent was removed under reduced pressure. The crude residue was purified by column chromatography (hexane/EtOAc, 0–10%) to yield the title compound (681 mg, 1.45 mmol, 73%) as a colorless oil. *R*_f_ = 0.17 (hexane); ^1^H NMR (400 MHz, CDCl_3_) δ 4.86 (d, *J* = 3.0 Hz, 1H, H-1), 3.64 (t, *J* = 8.9 Hz, 1H, H-3), 3.61–3.49 (m, 3H, H-5, H-6a, H-6b), 3.30 (t, *J* = 9.1, 1H, H-4), 3.19 (dd, *J* = 9.1, 3.1 Hz, 1H, H-2), 1.60 (dd, *J* = 7.0, 5.4 Hz, 1H, 6-OH), 0.04 (s, 9H, Si-CH_3_), −0.00 (s, 9H, Si-CH_3_), −0.00 (s, 9H, Si-CH_3_), −0.01 (s, 9H, Si-CH_3_); ^13^C{^1^H} NMR (101 MHz, CDCl_3_) δ 93.1 (C1), 73.2 (C2), 72.7 (C3), 71.1 (C5), 71.0 (C4), 61.0 (C6), 0.3 (Si-CH_3_), −0.0 (Si-CH_3_), −0.5 (Si-CH_3_), −0.7 (Si-CH_3_); HRMS *m*/*z* (ES^+^) found: (M + Na)^+^ 491.2103; C_18_H_44_O_6_NaSi_4_ requires M^+^ 491.2107. Data matched those previously reported.^44^

#### Glucose-Gemcitabine Conjugate **23**

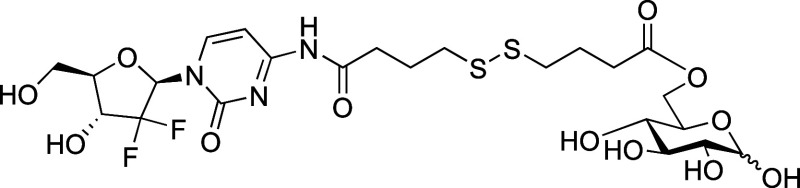



To a solution of **S2** (60.0 mg, 51.6 μmol, 1.0 equiv) in THF (5 mL) was added 1 M TBAF in THF (0.16 mL, 41.0 mg, 0.155 mmol, 3.0 equiv) at 0 °C. After 1 h of stirring at this temperature, TLC analysis revealed reaction completion (*R*_f_ = 0.30, DCM/MeOH, 85:15) and the mixture was diluted with DCM and washed with saturated aqueous NaHCO_3_ (2 × 5 mL). The combined organic phases were dried (MgSO_4_), filtered, and concentrated under reduced pressure. Purification by column chromatography (DCM/MeOH, 0–20%) delivered the title compound (18.0 mg, 27.9 μmol, 54%, Glc α/β, 1:1) as a colorless oil. *R*_f_ = 0.30 (DCM/MeOH, 85:15); ^1^H NMR (400 MHz, MeOD) δ 8.34 (d, *J* = 7.6 Hz, 1H, CH′), 7.49 (d, *J* = 7.6 Hz, 1H, CH′), 6.30–6.23 (m, 1H, H-1″), 5.08 (d, *J* = 3.7 Hz, 1H, 0.5H, H-1α), 4.48 (d, *J* = 7.8 Hz, 1H, H-1β), 4.39 (m, 1H), 4.30 (td, *J* = 12.2, 8.6 Hz, 1H, H-4″), 4.19 (m, 2H, 1H), 4.01–3.93 (m, 2.5H, H-5a″, H-3″), 3.81 (dd, *J* = 12.4, 2.8 Hz, 1H, H-5b″), 3.67 (t, *J* = 9.3 Hz, 0.5H), 3.47 (ddd, *J* = 9.1, 5.8, 2.1 Hz, 0.5H), 3.39–3.32 (m, 1.5H, H-3β, H-4α), 3.14 (dd, *J* = 8.8, 8.1 Hz, 0.5H), 2.76 (dt, *J* = 9.7, 7.2 Hz, 4H, 2 × CH_2_), 2.61 (t, *J* = 7.3 Hz, 2H, CH_2_), 2.48 (t, *J* = 7.3 Hz, 2H, CH_2_), 2.12–1.95 (m, 4H, 2 × CH_2_); ^13^C{^1^H} NMR (101 MHz, MeOD) δ 173.3 (C-NH, C4′), 166.3 (C=O), 156.5 (C=O), 144.6 (CH, C6′), 96.93 (CH, C5′), 96.86 (C1β), 92.6 (C1α), 84.8–84.1 (m, C1″), 80.9 (d, *J* = 8.8 Hz, C3″), 76.5, 74.8, 73.9, 73.3, 72.4, 70.5, 70.31, 70.29, 69.3, 69.2 (C4″), 63.5, 59.1 (C5″), 37.1, 37.0, 35.0, 31.9, 31.8, 31.7, 24.0, 23.9, 23.7; ^19^F NMR (377 MHz, MeOD) δ −119.18 (dd, *J* = 238.8, 11.3 Hz), −120.10 (d, *J* = 244.7 Hz); HRMS *m*/*z* (ES^–^) found: (M – H)^−^ 644.1398; C_23_H_32_O_12_ N_3_F_2_S_2_ requires M^–^ 644.1401.

## Conclusions

We establish the synthesis of a novel gemcitabine glycoconjugate. An initial approach sought coupling of 6-deoxy-6-thio glucose to gemcitabine using a redox reactive linker. Unfortunately, attempts to remove the pyranose acetate protecting groups resulted in linker hydrolysis at the *N*4 amide of the pyrimidine. Alternatively, a protecting group-free approach was implemented, relying on thiol–pyridyl disulfide exchange for coupling. However, rapid oxidation of one of the exchange partners, 6-deoxy-6-thio glucose, to the corresponding disulfide prevented the reaction from progressing. The redox-responsive disulfide was thus relocated into the linker and alternate pyranose protecting groups (*O*-TMS) were implemented, granting access to the required glycoconjugate. Cytotoxicity studies revealed increased toxicity of the glucose-gemcitabine conjugate against hormone-resistant PC3 cell lines when compared with a hormone-sensitive LNCaP line.

## Supporting Information Available

Experimental chemical and biological procedures and relevant 1D and 2D NMR spectra for all compounds (PDF)
