# Supplementary material for: Exploring a Gemcitabine-Glucose Hybrid as a Glycoconjugate Prodrug
Source: ACS Omega. 2024 Jul 9;9(29):31703–13. doi: 10.1021/acsomega.4c02417 (PMC11270703; doi:10.1021/acsomega.4c02417)

Exploring a gemcitabine-glucose hybrid as a glycoconjugate prodrug

Jack Porter,[a] Amanda R. Noble,[b] Nathalie Signoret,[b] Martin A. Fascione[c] and Gavin J. Miller*[a]

[a] Centre for Glycoscience and School of Chemical and Physical Sciences, Keele University, Keele, Staffordshire, ST5 5BG, United Kingdom.

[b] Hull York Medical School, University of York, Heslington, York, YO10 5DD, UK.

[c] Department of Chemistry, University of York, Heslington, York, YO10 5DD, UK.

Supporting Information

Table of Contents

1.1 General Experimental S3
1.2 Synthesis and Chemical Characterisation S3
2. Conditions for Attempted Deprotection of Glycoconjugate **12** S5
3. HPLC Analysis of **23** S6
4. Cytotoxicity Evaluation S7
5. References...S8
6. NMR Spectra...S9

1.1 General Experimental

Assignment of ^{1}H and ^{13}C atoms in NMR analysis follows the ring numbering systems below.

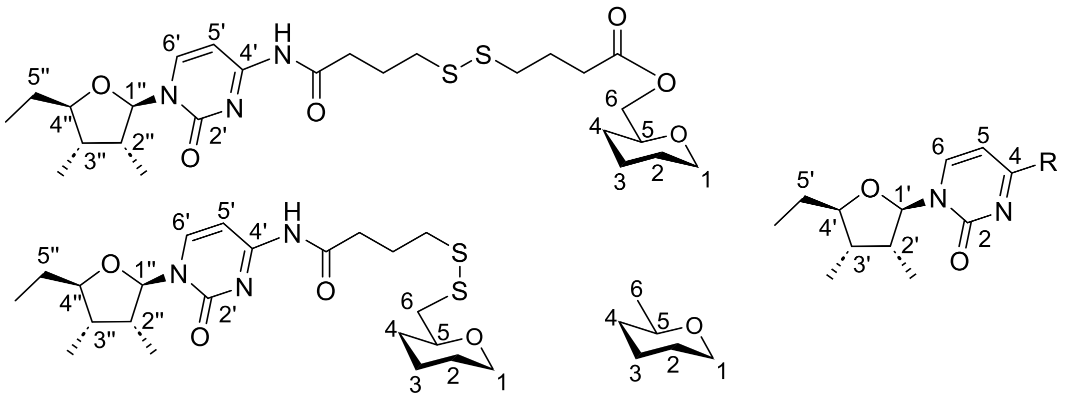

1.2 Synthesis and Chemical Characterisation

Bis(2-pyridinyl) disulfide S1

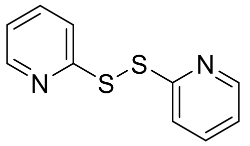

To a solution of 2-mercaptopyridine (2.50 g, 22.5 mmol, 1.0 equiv.) in DMSO (2 mL) was added I_2 (571 mg, 2.25 mmol, 0.1 equiv.) the resulting mixture was stirred at RT for 1 hr. TLC analysis revealed complete consumption of the starting material (R_f = 0.54, hexane/EtOAc, 1:1) the mixture was diluted with DCM (50 mL) and washed with sat. aq. $Na_2S_2O_3$· (100 mL) and brine (100 mL). The combined organic phases were dried ($MgSO_4$), filtered and concentrated under reduced pressure. The crude residue was purified by column chromatography (hexane/EtOAc, 0-50%) to yield the title compound (3.79 g, 17.2 mmol, 77%) as a yellow oil. R_f = 0.54 (hexane/EtOAc, 1:1); ^{1}H NMR (400 MHz, $CDCl_3$) δ 8.49 – 8.42 (m, 2H, ArH), 7.66 – 7.58 (m, 4H, ArH), 7.17 – 7.06 (m, 2H, ArH); ^{13}C {1H} NMR (101 MHz, $CDCl_3$) δ 158.9, 149.6, 137.4, 121.1, 119.7; HRMS *m/z* (ES^+) Found: $(M+H)^+$ 221.0197, $C_{10}H_9N_2S_2$ requires M^+ 221.0202. Data matched those previously reported.[1]

Protected glucose-gemcitabine conjugate S2

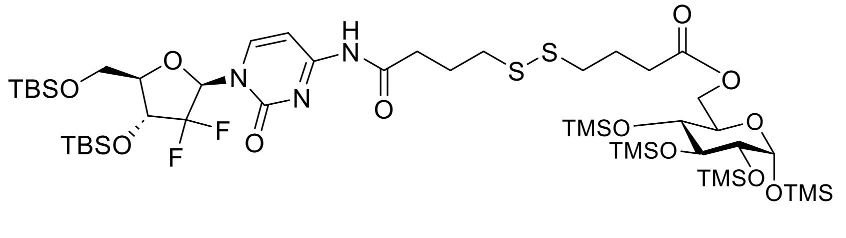

To a solution of **21** (95.0 mg, 0.13 mmol. 1.0 equiv.) in DCM (15 mL) was added EDC (62.0, 0.40 mmol, 3.0 equiv.) followed by DMAP (1.62 μg, 13.3 μmmol, 0.1 equiv.). The reaction mixture was stirred at RT for 15 min. Glycoside **22** (94.0 mg, 0.20 mmol, 1.5 equiv.) was next

added and the reaction mixture stirred for a further 1 hr. TLC analysis revealed reaction completion (R_f =0.25, hexane/EtOAc, 7:3) the reaction mixture was diluted with DCM (50 mL) and washed with sat. aq. $NaHCO_3$ (2 × 50 mL). The combined organic phases were dried ($MgSO_4$), filtered and concentrated under reduced pressure. Purification by column chromatography (hexane/EtOAc, 0-30%) delivered the title compound (70.0 mg, 60.2 µmol, 45%) as a colourless oil. R_f = 0.25 (hexane/EtOAc), $[\alpha]_D^{22}$ = +59.78 (*c* = 1.0, $CHCl_3$); ^1H NMR (400 MHz, $CDCl_3$) δ 8.59 (bs, 1H, NH), 7.94 (d, *J* = 7.6 Hz, 1H, CH'), 7.27 (d, *J* = 7.5 Hz, 1H, CH'), 6.20 (dd, $J_{H\text{-}1''\text{-}Fa/Fb}$ = 10.2, 3.8 Hz, 1H, H-1''), 4.87 (d, *J* = 3.0 Hz, 1H, H-1), 4.27 – 4.15 (m, 2H, H-6a, H-3''), 3.97 – 3.86 (m, 2H, H-6b, H-5a''), 3.82 (d, *J* = 8.1 Hz, 1H, H-4''), 3.78 (ddd, *J* = 9.7, 5.2, 2.2 Hz, 1H, H-5), 3.67 (m, 2H, H-3, H-5b''), 3.30 (dd, *J* = 9.7, 8.6 Hz, 1H, H-4), 3.23 (dd, *J* = 9.1, 3.1 Hz, 1H, H-2), 2.59 (aq, *J* = 6.8 Hz, 6H, 2 × CH_2), 2.46 (t, *J* = 7.2 Hz, 3H, CH_2), 2.36 (m, 2H, CH_2), 2.01 – 1.84 (m, 6H, 2 × CH_2), 0.82 (s, 9H, Si-*t*Bu), 0.77 (s, 9H, Si-*t*Bu), 0.02 (s, 9H, Si-CH_3), 0.02 (s, 9H, Si-CH_3), 0.01 (s, 9H, Si-CH_3), 0.00 – -0.01 (m, 15H, Si-CH_3), -0.03 (s, 3H, Si-CH_3), -0.13 (s, 3H, Si-CH_3); ^{13}C {1H} NMR (101 MHz, $CDCl_3$) δ 171.9 (C=O), 171.4 (C-NH, C4'), 161.4 (C=O), 153.9 (C=O), 143.3 (CH, C6'), 95.6 (CH, C5'), 93.0 (C1), 84.03 – 83.16 (m, C1''), 80.5 (d, ^2J = 8.6 Hz, C3''), 73.0 (C2), 72.8 (C3), 71.4 (C4), 68.9 (C5), 68.48 (C4''), 63.0 (C6), 59.0 (C5''), 36.6 (CH_2), 34.7 (CH_2), 31.4 (CH_2), 31.4 (CH_2), 24.9 (Si-*t*Bu), 24.6 (Si-*t*Bu), 23.1 (CH_2), 22.7 (CH_2), 17.4 (Si-*t*Bu), 17.1 (Si-*t*Bu), 0.28 (Si-CH_3), -0.5 (Si-CH_3), -0.8 (Si-CH_3) - 1.0, (Si-CH_3) -5.7 (Si-CH_3), -6.3 (Si-CH_3), -6.39 (Si-CH_3), -6.41 (Si-CH_3); ^{19}F NMR (377 MHz, $CDCl_3$) δ -115.96 (dd, *J* = 239.5, 11.9 Hz), -117.37 (dt, *J* = 239.3, 10.5 Hz).

2. Conditions for Attempted Deprotection of Glycoconjugate 12

Table S1: Screened conditions for deprotection of glycoconjugate **12**

Deprotection conditions	Reaction outcome
**12** (1.0 equiv.), TBAF (4.0 equiv.), THF, RT	Amide hydrolysis
**12** (1.0 equiv.), Na (0.1 equiv.), MeOH, RT	Amide hydrolysis
**12** (1.0 equiv.), MeOH/HCl 10% (4:1), 70 °C	Amide hydrolysis
**12** (1.0 equiv.), TsOH (4.0 equiv.), DCM/MeOH (4:1), RT	Amide hydrolysis
**12** (1.0 equiv.), 7M NH_3 in MeOH (9.0 equiv.), 40 °C	Amide hydrolysis

3. HPLC analysis of 23

A sample of **23** was dissolved in MeCN (v/v, 1 mg mL^{-1}), and injected onto a reverse phase column (see general experimental) and a chromatogram obtained at a flow rate of 5 ml min^{-1} using the following gradient system: (A) H_2O and (B) MeCN: 100% A → 100% B (20.0 min).

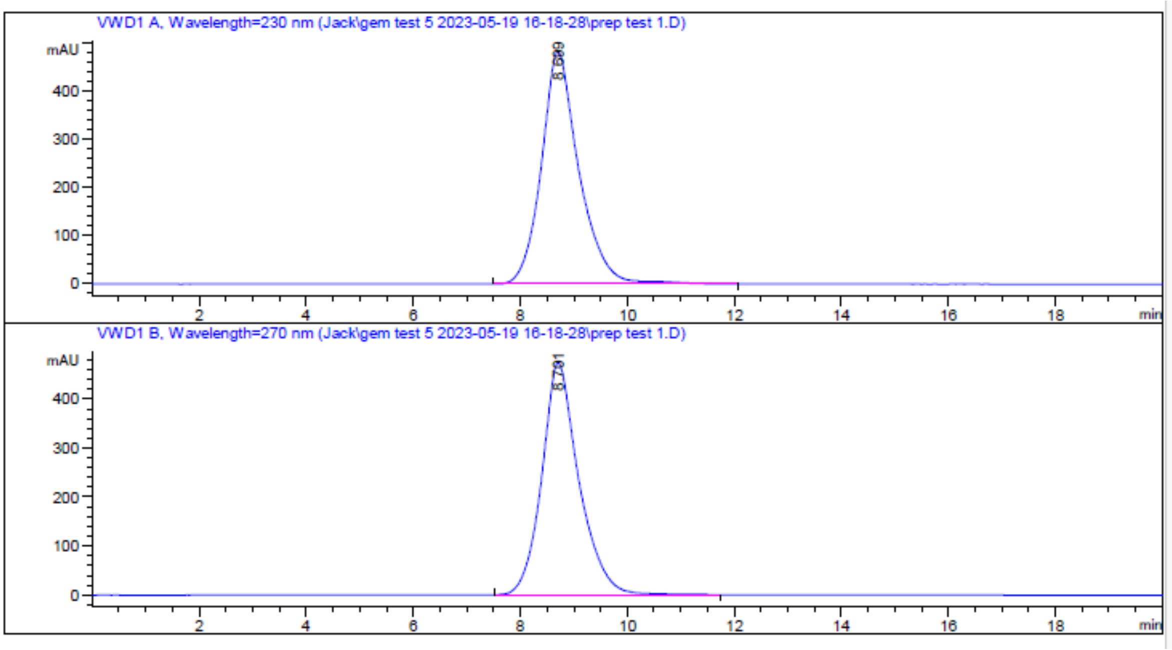

Peak #	RetTime [min]	Type	Width [min]	Area [mAU*s]	Height [mAU]	Area %
1	8.699	BB	0.7176	2.34878e4	481.11115	100.0000
Totals :				2.34878e4	481.11115	

Signal 2: VWD1 B, Wavelength=270 nm

Peak #	RetTime [min]	Type	Width [min]	Area [mAU*s]	Height [mAU]	Area %
1	8.701	BB	0.7083	2.27295e4	473.25436	100.0000
Totals :				2.27295e4	473.25436	

Figure S1: HPLC chromatogram and purity of isolated **23**.

4. Cytotoxicity Evaluation

Cell Culture: Cells were seeded at 5,000 cells/well of a 96 well plate using the appropriate media 24 hrs prior to addition of treatments or at the same time. To wells was added 100 µL of appropriate media containing DMSO vehicle or serial dilutions of glucose-gemcitabine **22** over the concentration range 0.001-3 µM, in the presence or absence of 12.5 µM GLUT1 inhibitor Phloretin. Cells were incubated for 72 hrs at 37 °C, 5% CO_2. Subsequently cell viability was measured by adding 12 µL MTS ([3-(4,5-dimethylthiazol-2- yl)-5-(3-carboxymethoxyphenyl)-2-(4-sulfophenyl)-2H-tetrazolium) (Promega, Southampton, UK) and incubating at 37 °C for 2 hrs prior to reading the absorbance at 490 nm.

Table S1: Details of cell lines and media requirements.

Cell Line	Origin	Culture medium
**LNCaP**	**Prostate cancer**	**RPMI 1640 + 10% FCS + 1% L-glutamine + 1% PenStrep**
**PC3**	**Prostate cancer bone metastasis**	**Hams F12 + 7% FCS + 1% L-glutamine + 1% PenStrep**

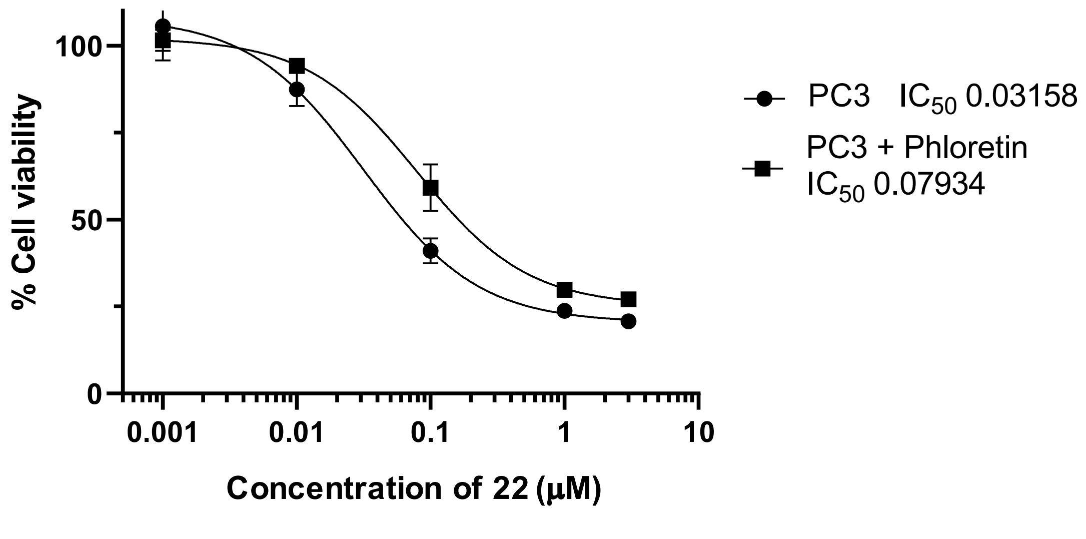

Figure S2: MTS cell viability assay measuring formazan product formed at 490 nm in metabolically active cells following treatment of PC3 cells with varying concentrations of **22** for 72 h in the presence and absence of 12.5 µM GLUT1 inhibitor Phloretin.

5. References

1 Chen, Z.; Wang, J.C.; Du, J. Q.; Kan, X.; Sun, T.; Kan, J.L.; Dong, Y. B. Construction of Multifunctional Covalent Organic Frameworks for Photocatalysis. *Chem. Eur. J.* **2024**, *30*, e202303497.

6. NMR Spectra

Figure S3 ^{1}H NMR (400 MHz, CDCl$_3$): Methyl 2,3,4-tri-*O*-acetyl-6-deoxy-6-iodo-α-D-glucopyranoside 6

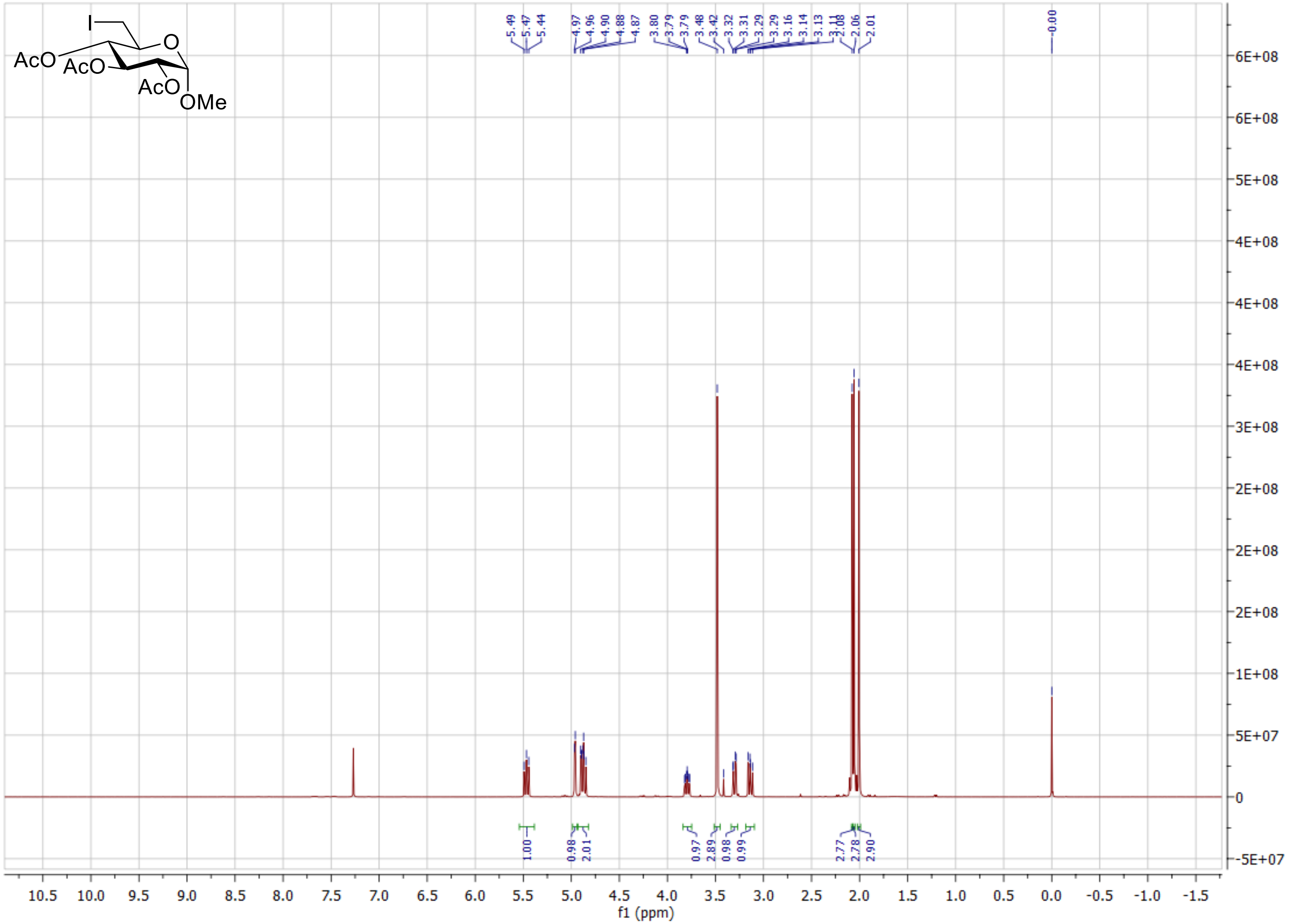

Figure S4 ^{13}C {1H} NMR (101 MHz, $CDCl_3$): Methyl 2,3,4-tri-*O*-acetyl-6-deoxy-6-iodo-α-D-glucopyranoside 6

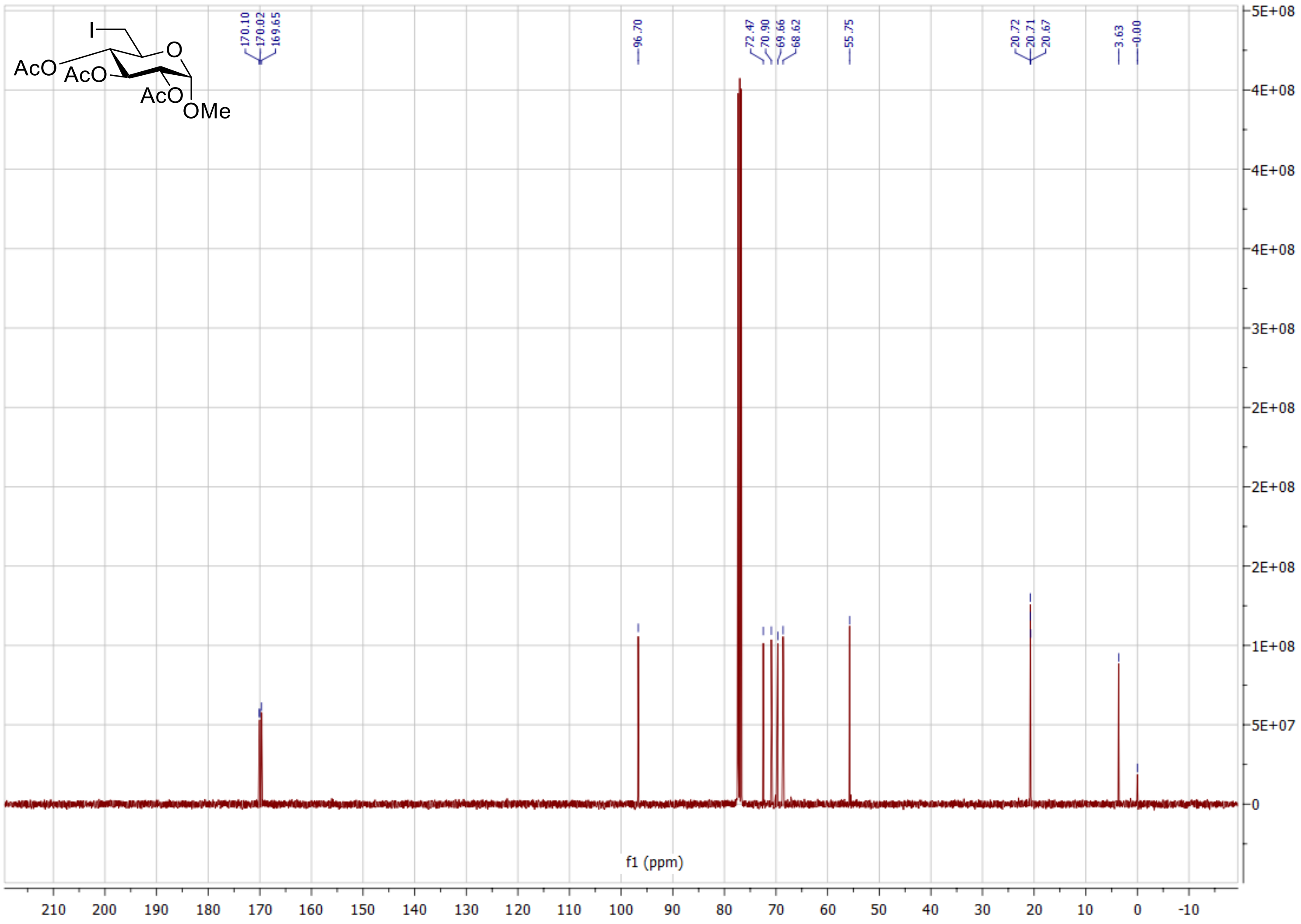

Figure S5 ^{1}H NMR (400 MHz, CDCl₃): Methyl 6-deoxy-6-*S*-acetyl-6-thio-2,3,4-tri-*O*-acetyl-α-D-glucopyranoside 7

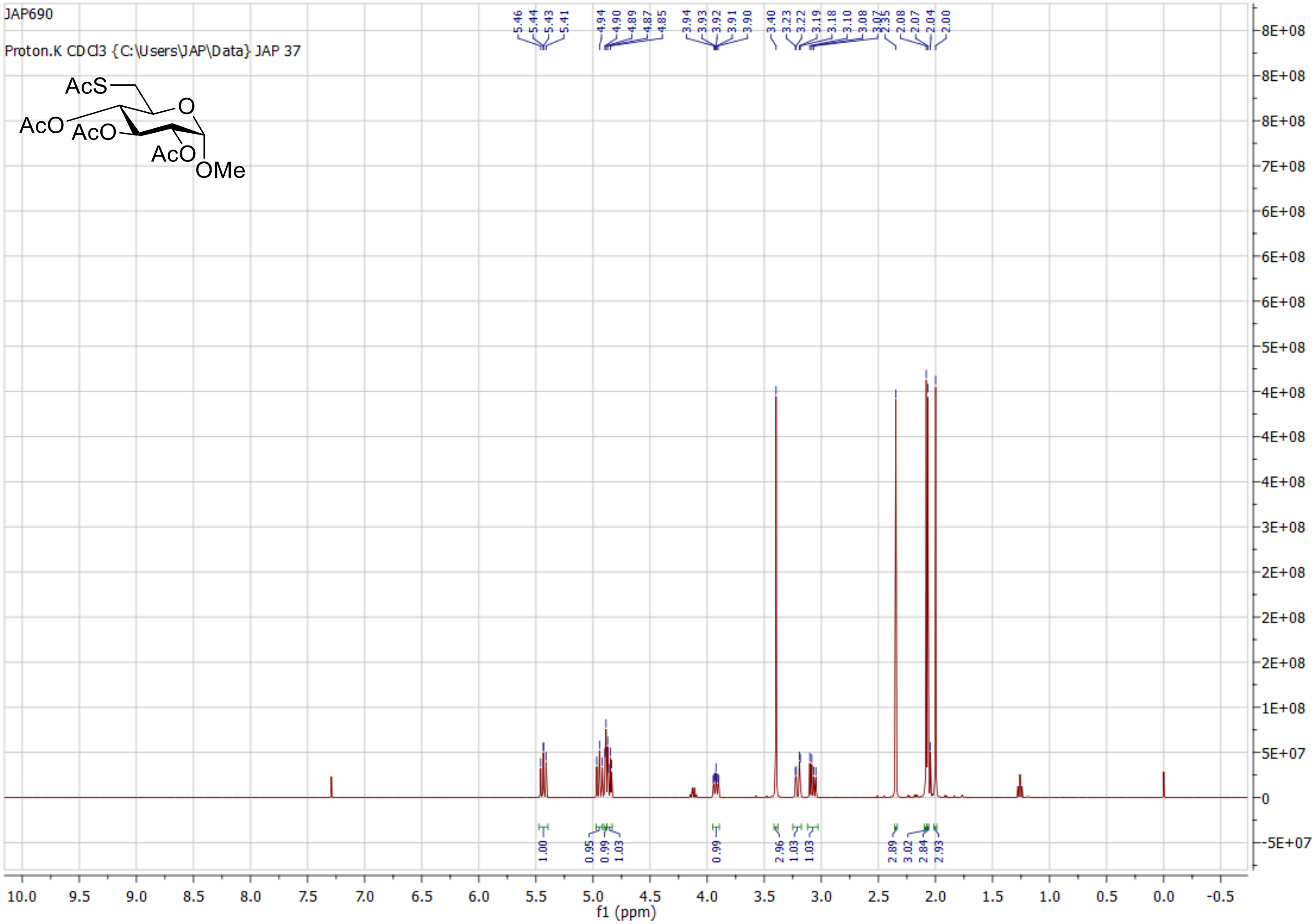

Figure S6 ^{13}C {^{1}H} NMR (101 MHz, $CDCl_3$): Methyl 6-deoxy-6-*S*-acetyl-6-thio-2,3,4-tri-*O*-acetyl-α-D-glucopyranoside 7

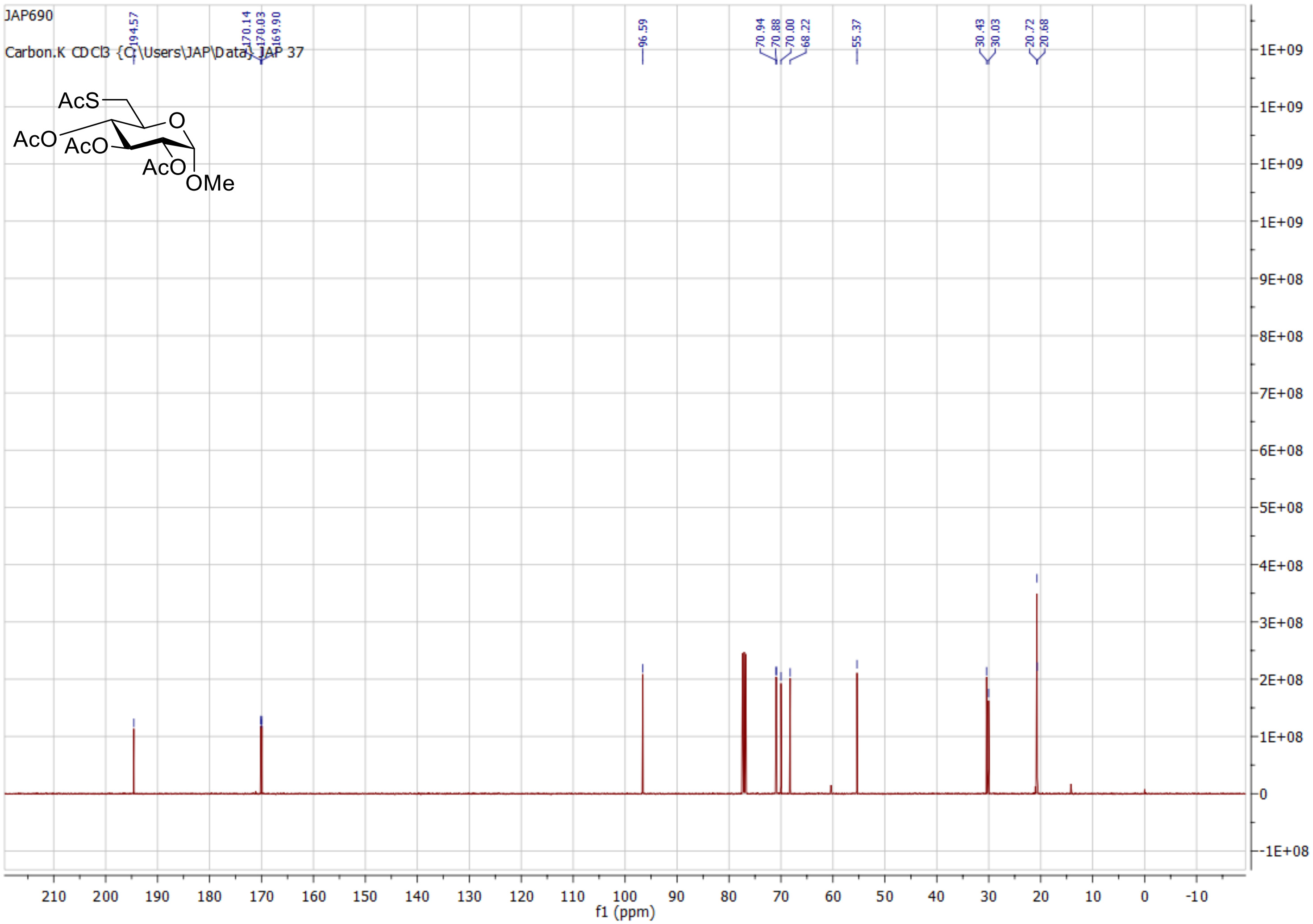

Figure S7 ^{1}H NMR (400 MHz, $CDCl_3$): Bis(methoxy 2,3,4-tri-*O*-acetyl-6-thio-α-D-glucopyranoside)-6,6'-disulfide 8

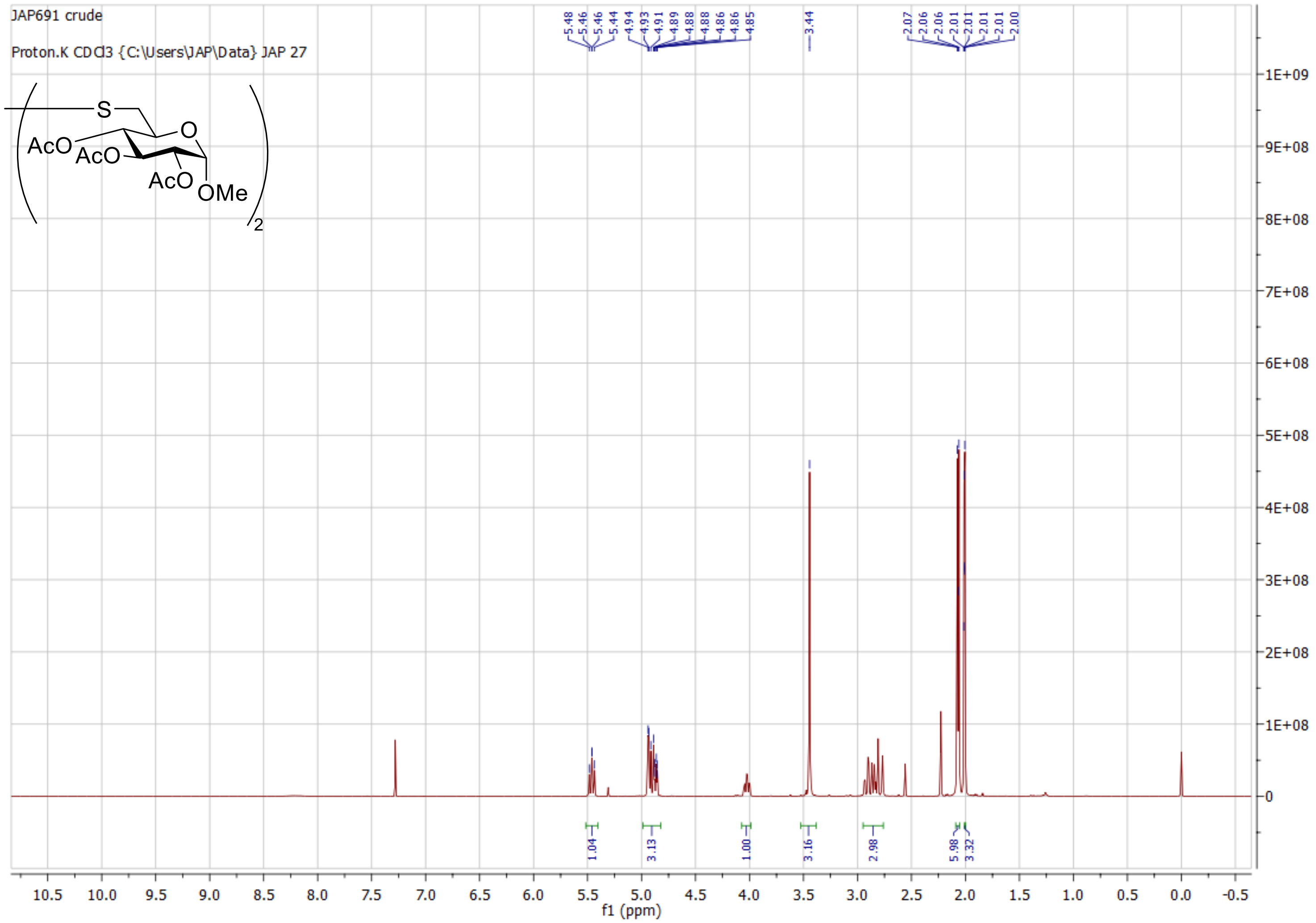

Figure S8 ^{13}C {^{1}H} NMR (101 MHz, $CDCl_3$): Bis(methoxy 2,3,4-tri-*O*-acetyl-6-thio-α-D-glucopyranoside)-6,6'-disulfide 8

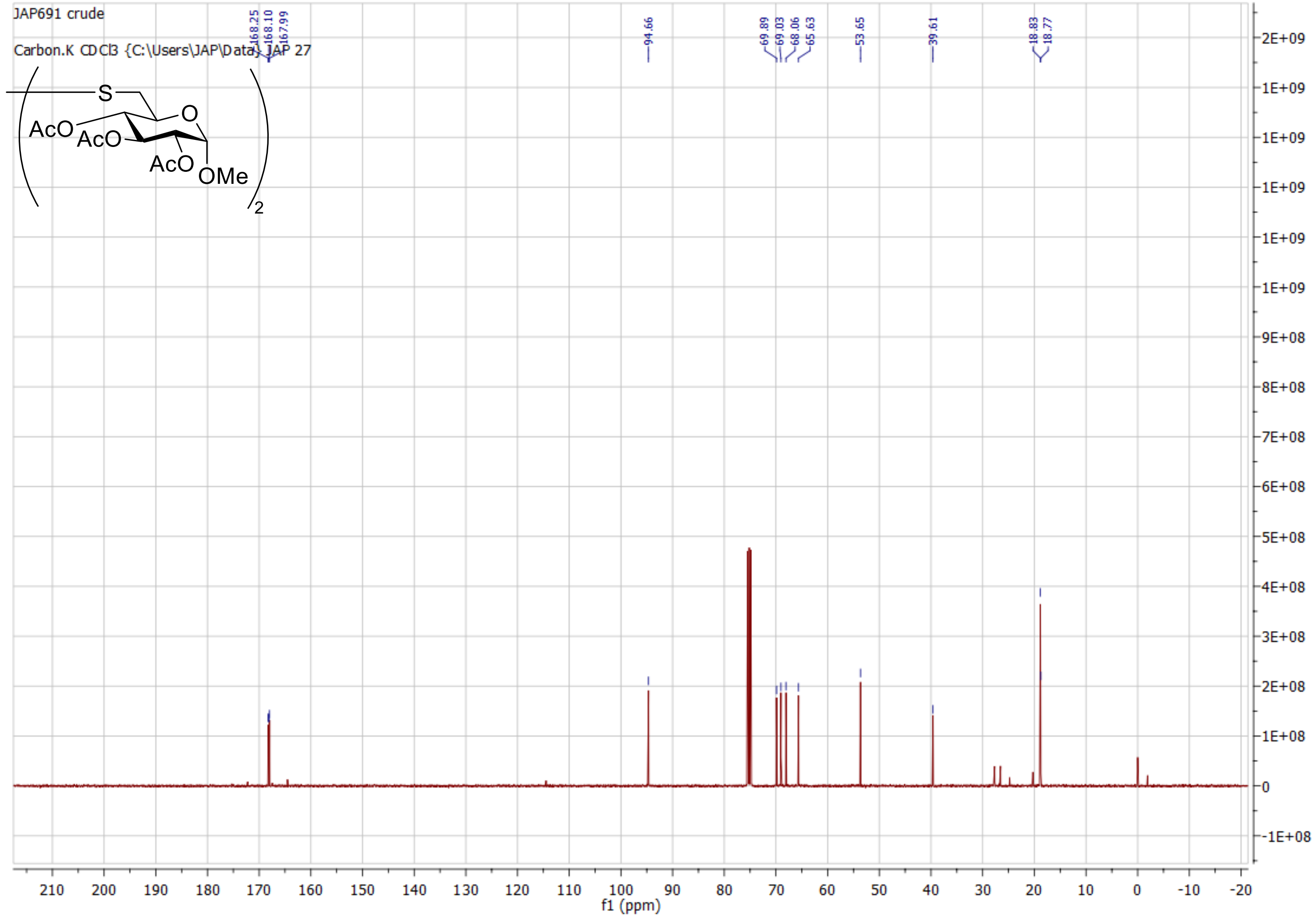

Figure S9 ^{1}H NMR (400 MHz, $CDCl_3$): Bis(1,2,3,4-tetra-*O*-acetyl-6-thio-α/β-D-glucopyranoside)-6,6’-disulfide 9

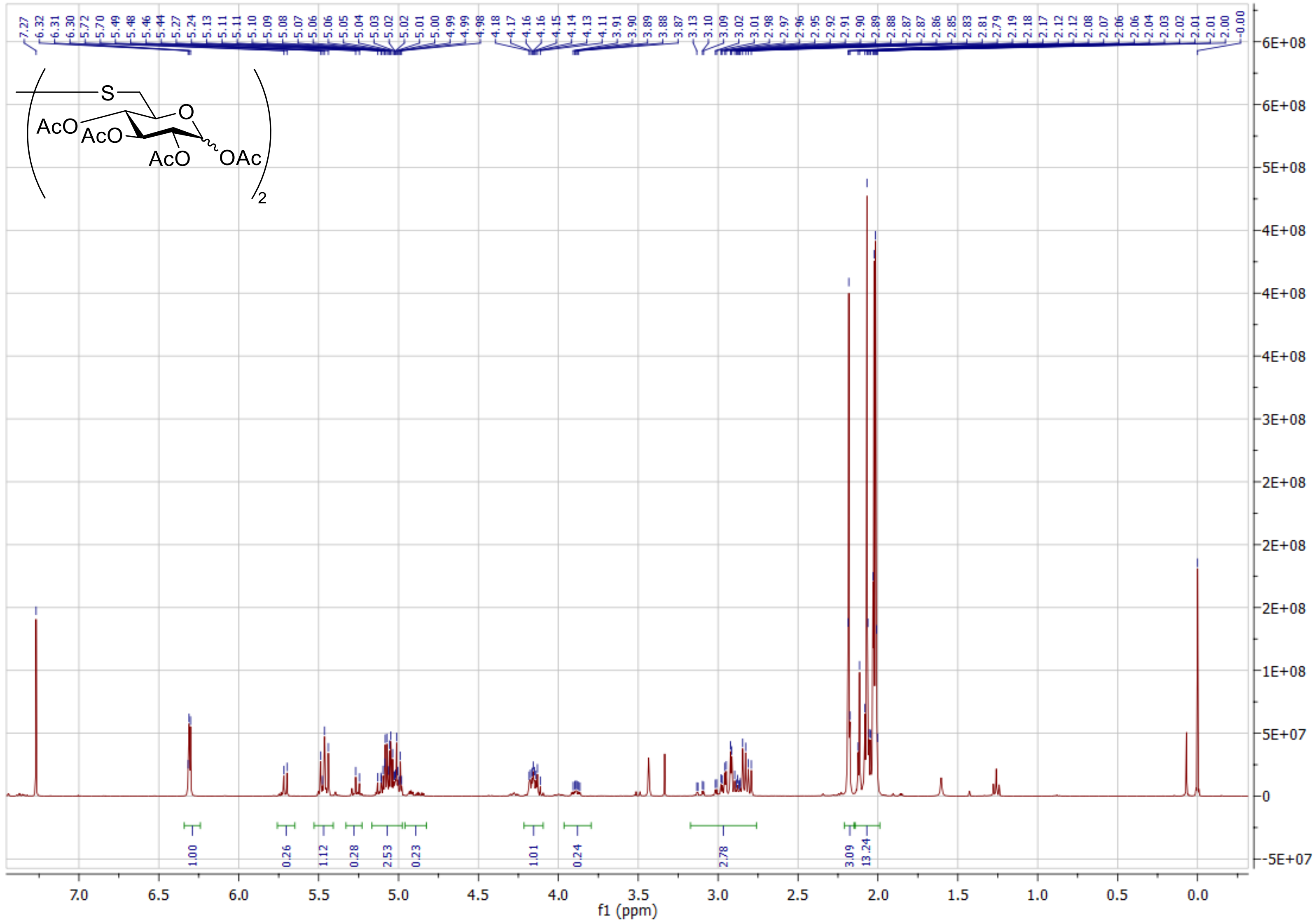

Figure S10 ^{13}C {^{1}H} NMR (101 MHz, $CDCl_3$): Bis(1,2,3,4-tetra-*O*-acetyl-6-thio-α/β-D-glucopyranoside)-6,6'-disulfide 9

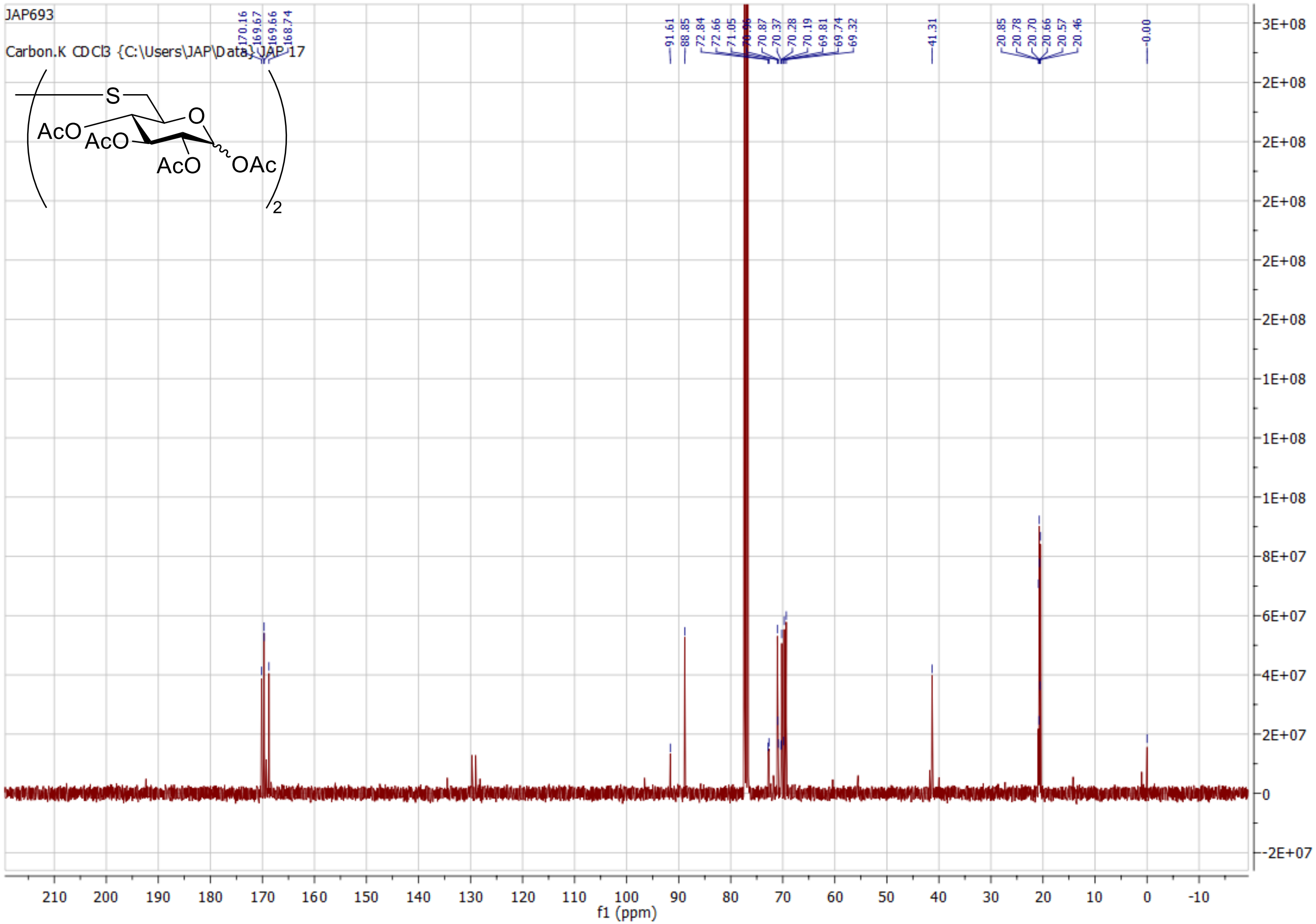

Figure S11 ^{1}H NMR (400 MHz, $CDCl_3$): 1,2,3,4-tetra-*O*-acetyl-6-deoxy-*S*-thiopropionic-acid-6-thio-α/β-D-glucopyranoside 10

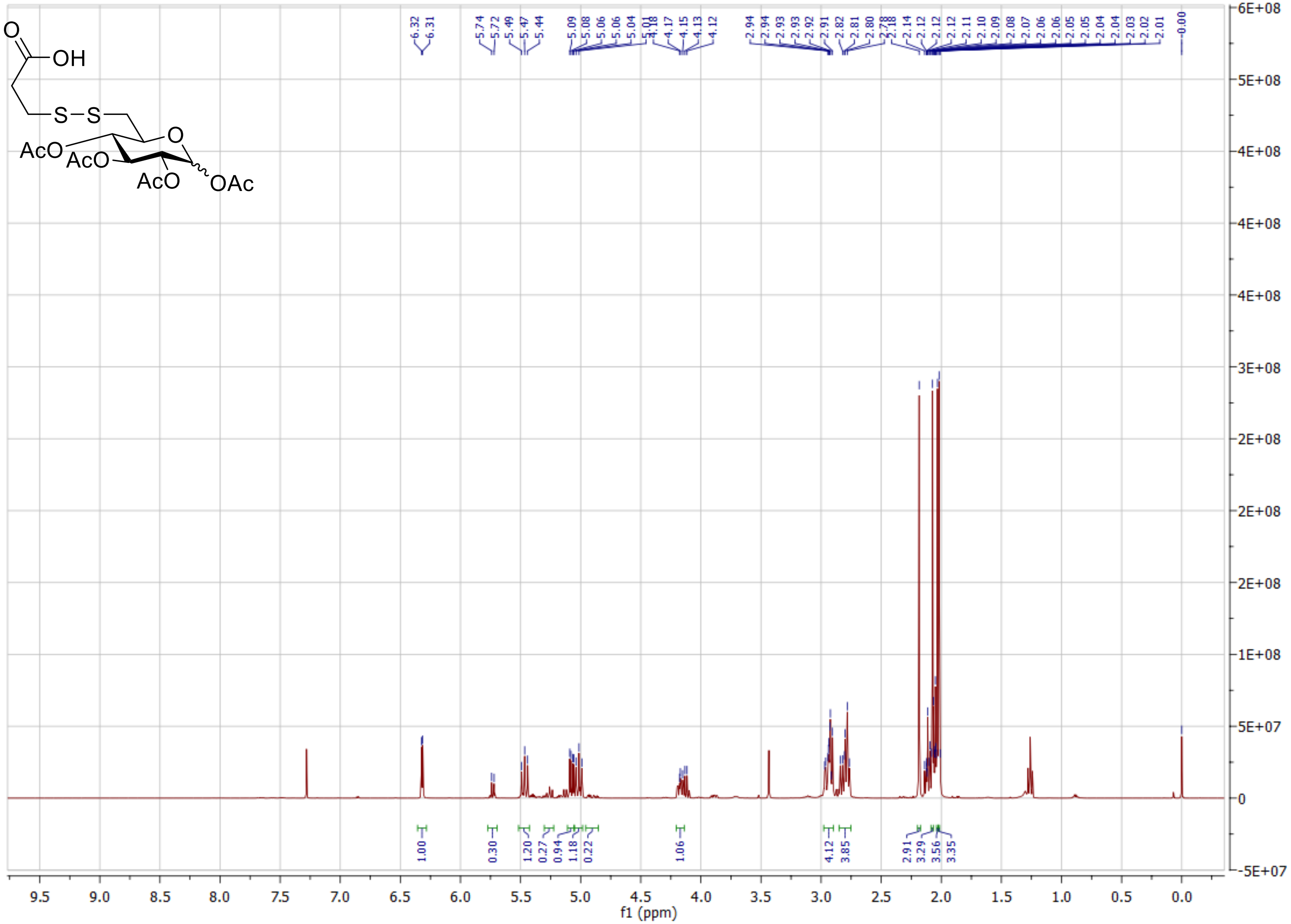

Figure S12 ^{13}C {1H} NMR (101 MHz, $CDCl_3$): 1,2,3,4-tetra-*O*-acetyl-6-deoxy-*S*-thiopropionic-acid-6-thio-α/β-D-glucopyranoside 10

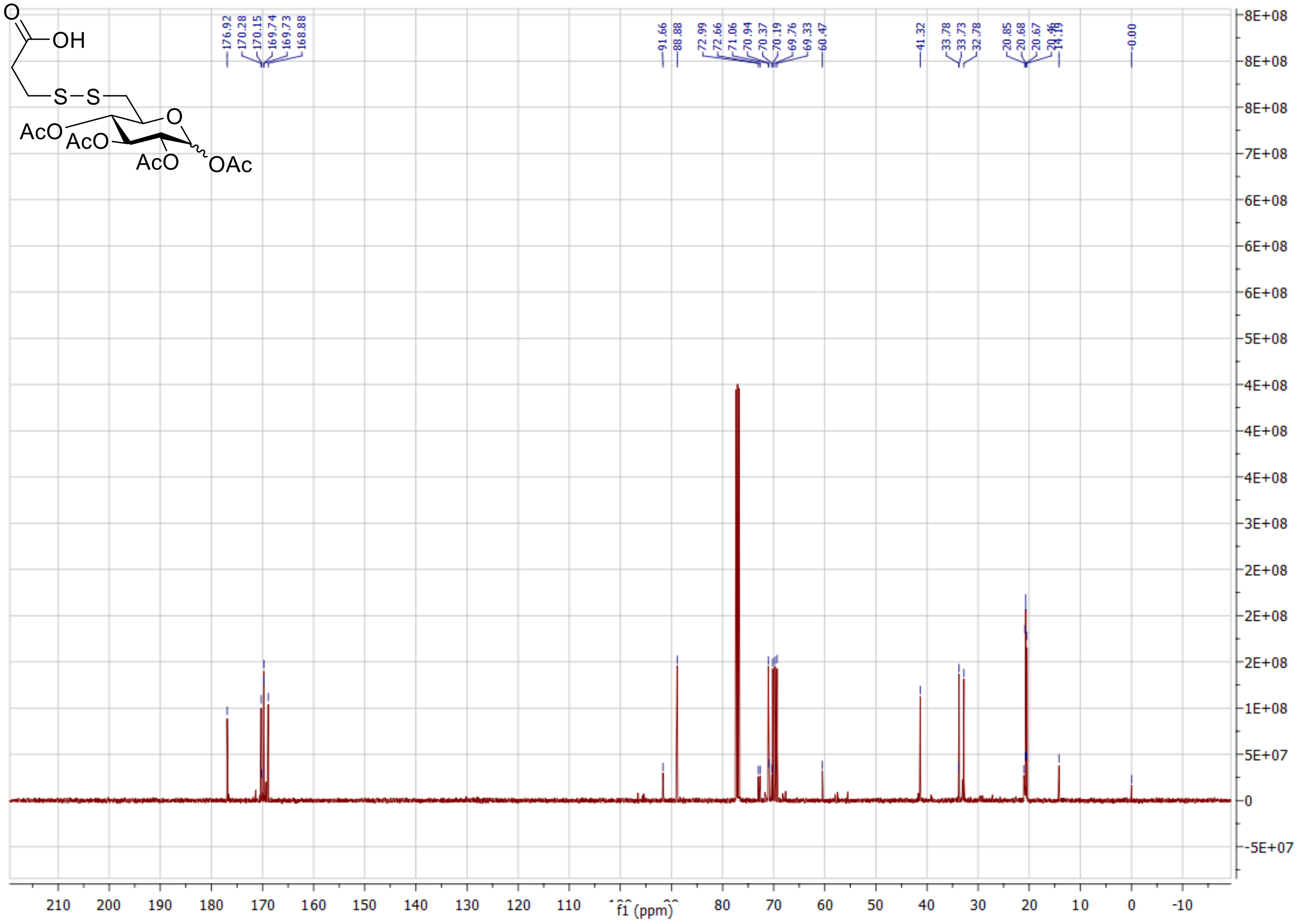

Figure S13 ¹H NMR (400 MHz, CDCl₃): 3’,5’-di-*O*-terbutyldimethylsilyl-2’-deoxy-2’-gem-difluoro-1’-(β-D-ribofuranosyl) cytosine 11

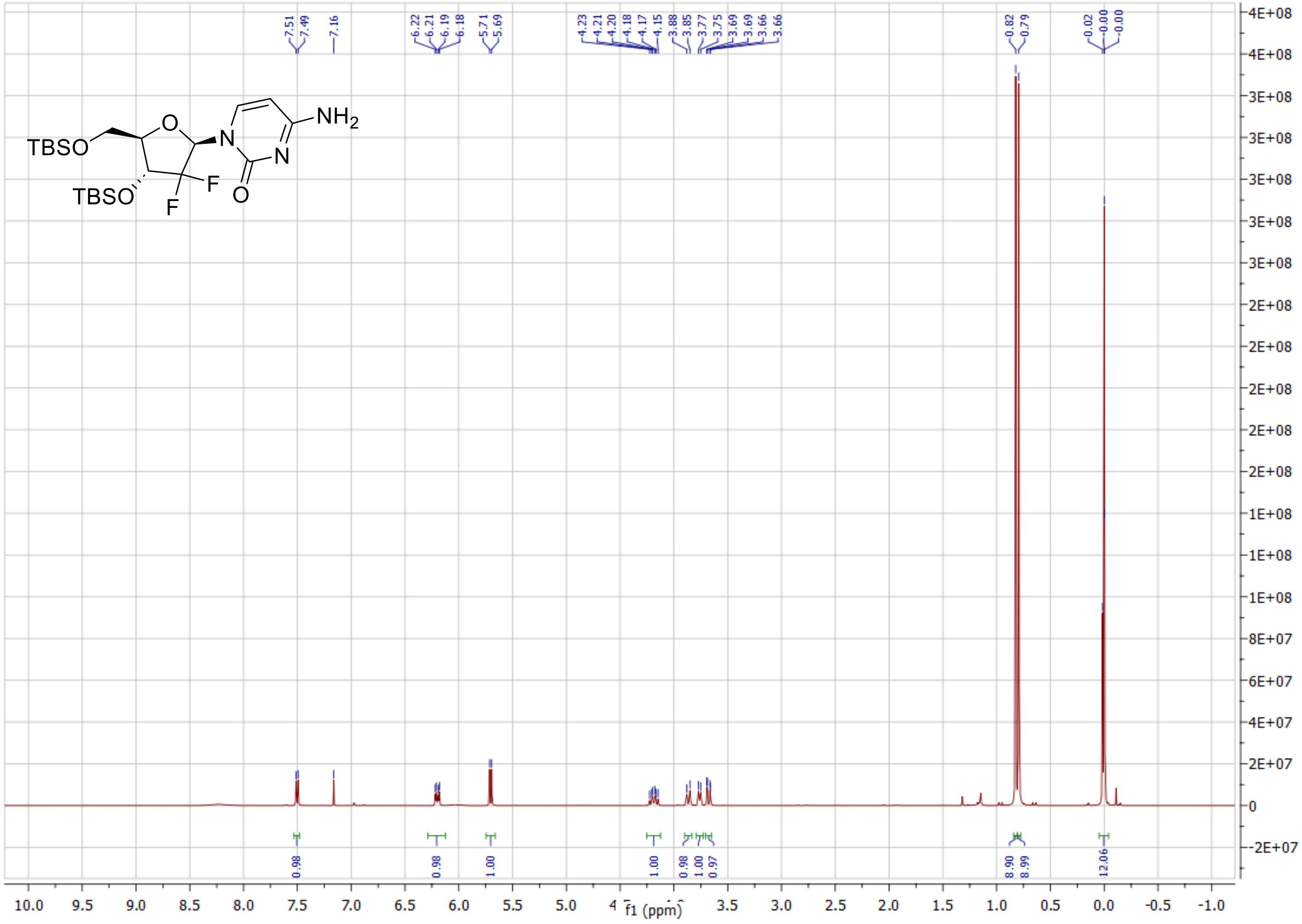

Figure S14 $^{13}C\ \{^1H\}$ NMR (101 MHz, $CDCl_3$): 3’,5’-di-*O*-terbutyldimethylsilyl-2’-deoxy-2’-gem-difluoro-1’-(β-D-ribofuranosyl) cytosine 11

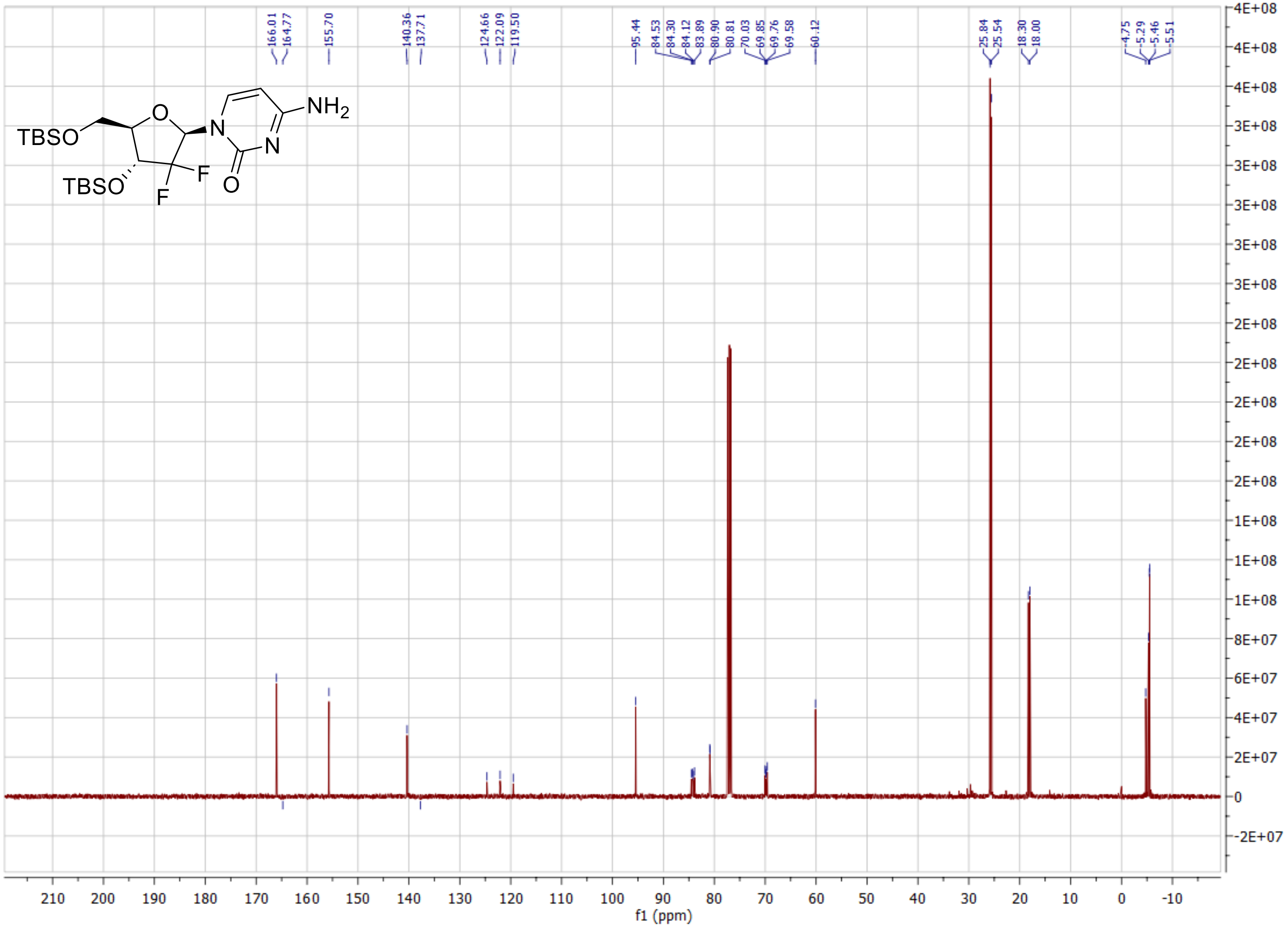

Figure S15 [19] F NMR (377 MHz, CDCl₃): 3',5'-di-*O*-TBS-2'-deoxy-2'-gem-difluoro-1'-(β-D-ribofuranosyl)cytosine 11

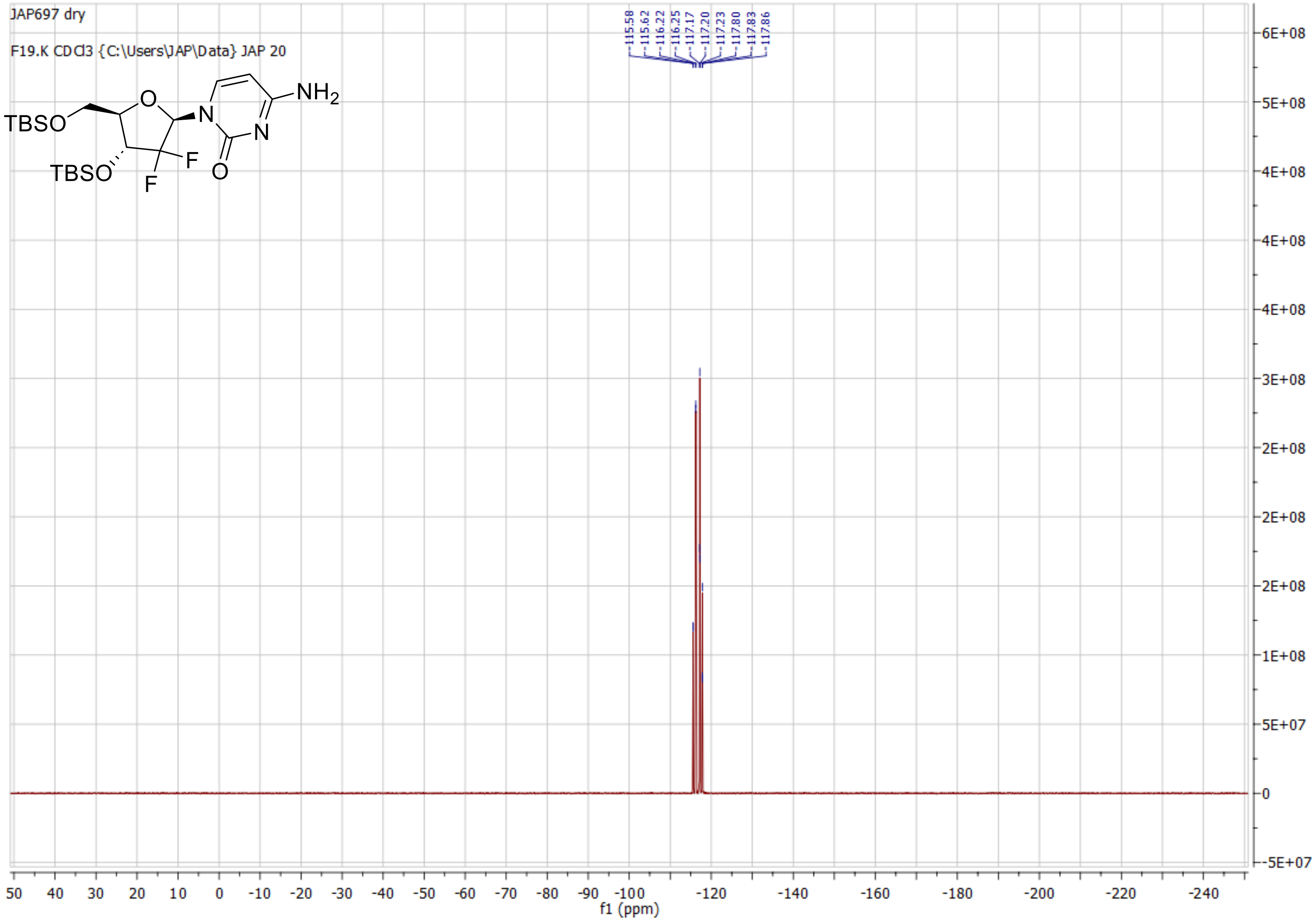

Figure S16 ^{1}H NMR (400 MHz, CDCl₃): Protected glucose-gemcitabine conjugate 12

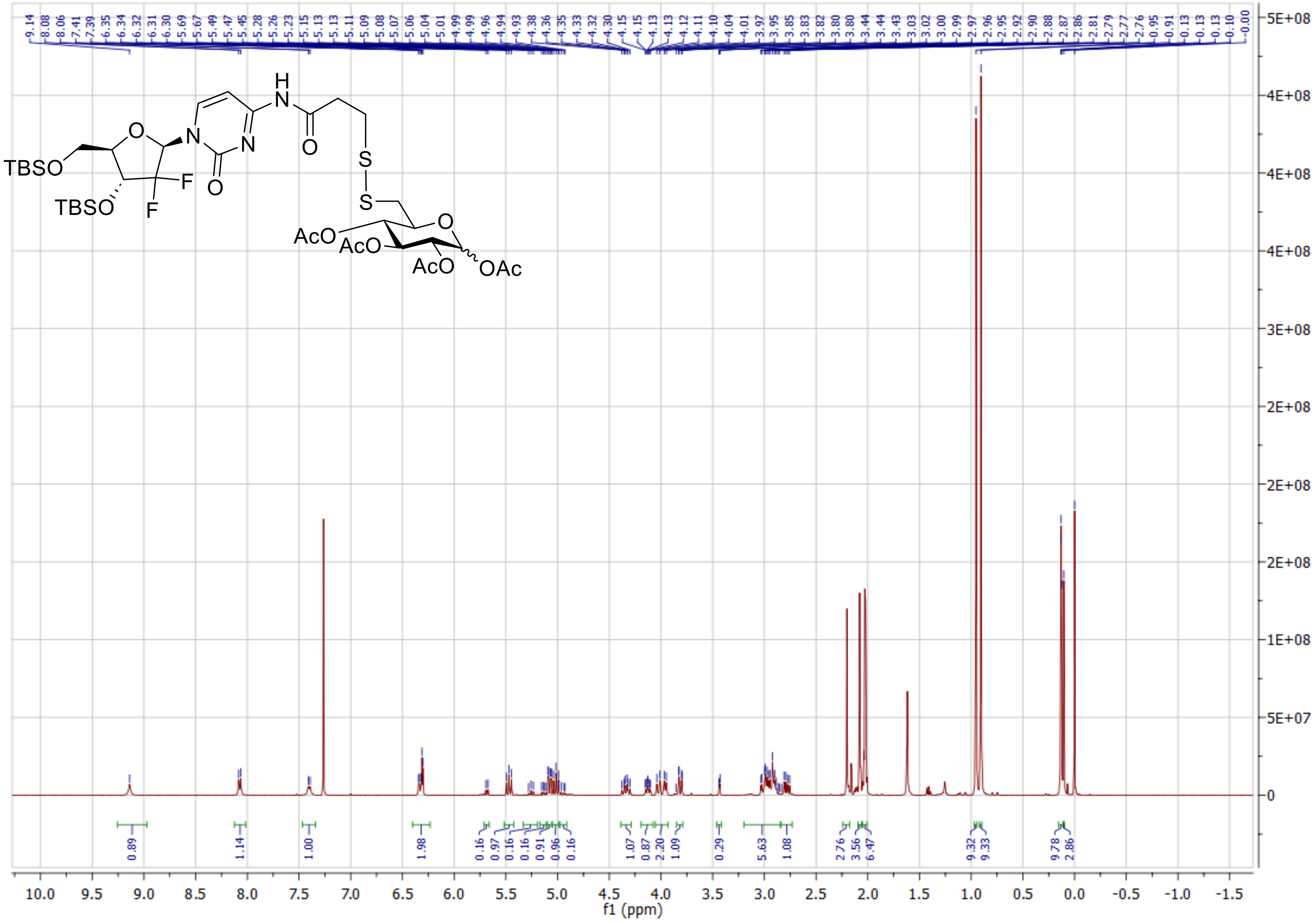

Figure S17 ^{13}C {^{1}H} NMR (100 MHz, $CDCl_3$): Protected glucose-gemcitabine conjugate 12

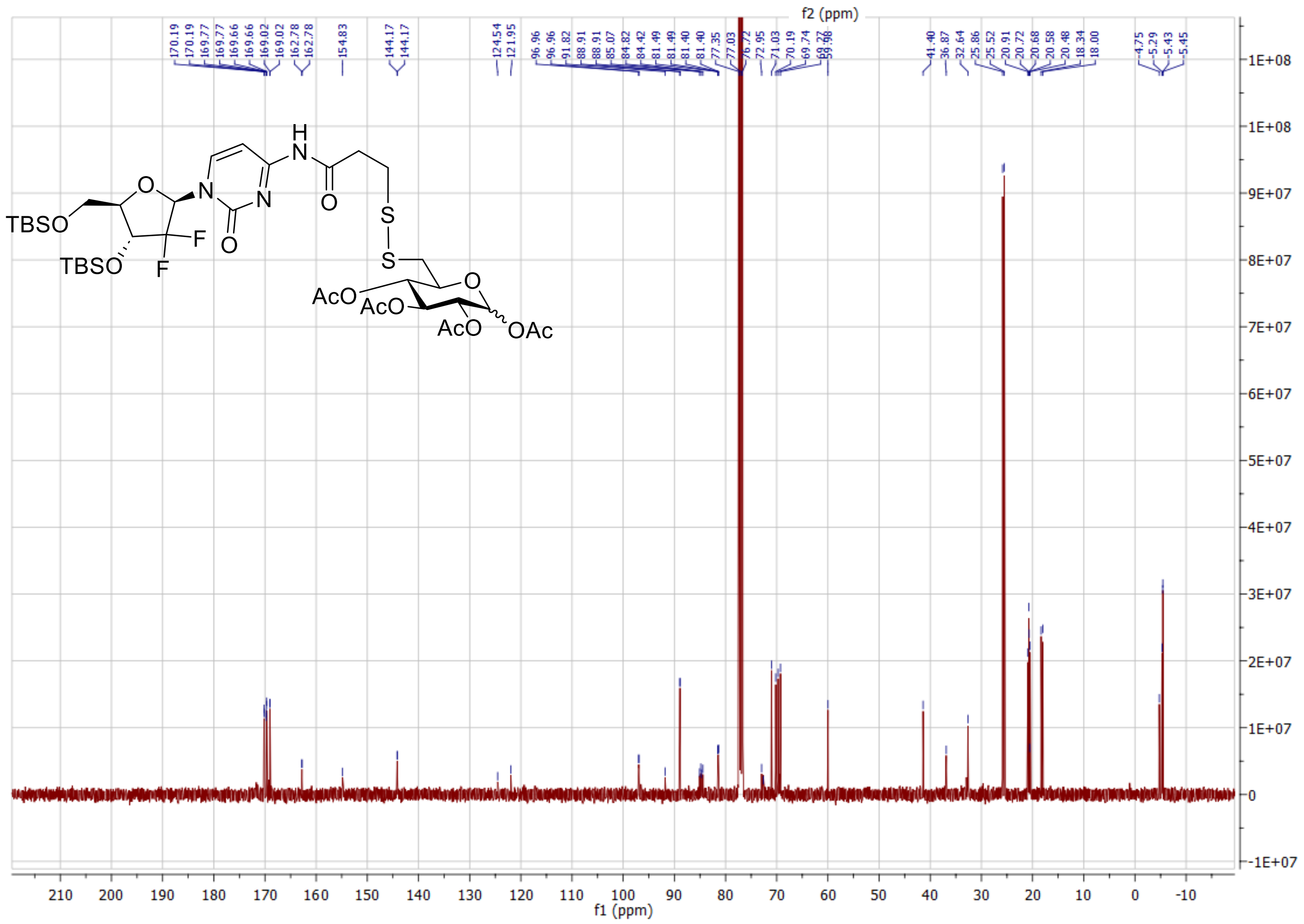

Figure S18 ^{19}F NMR (377 MHz, $CDCl_3$): Protected glucose-gemcitabine conjugate 12

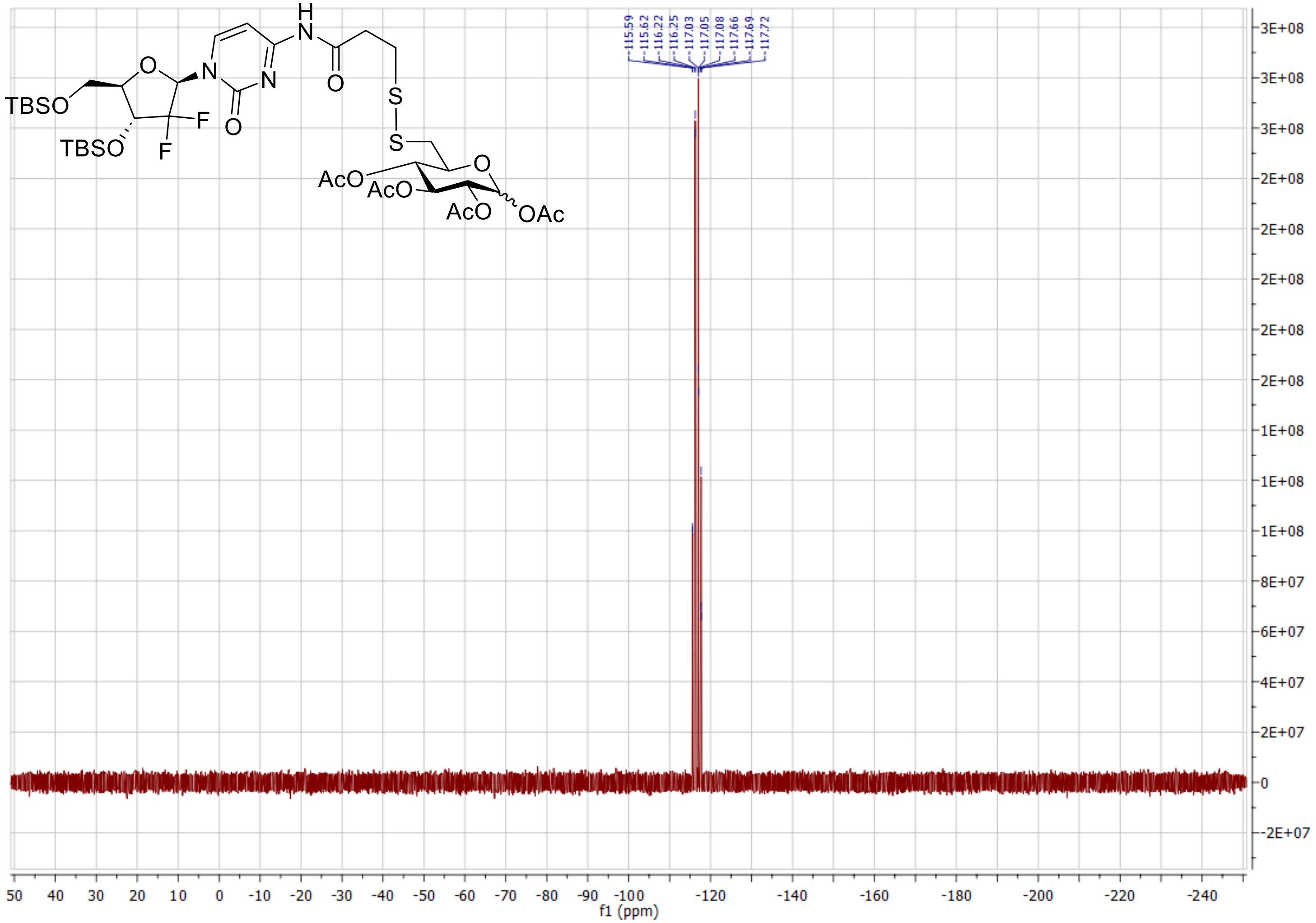

Figure S19 ^{1}H NMR (400 MHz, $CDCl_3$): Bis(2-pyridinyl) disulfide S1

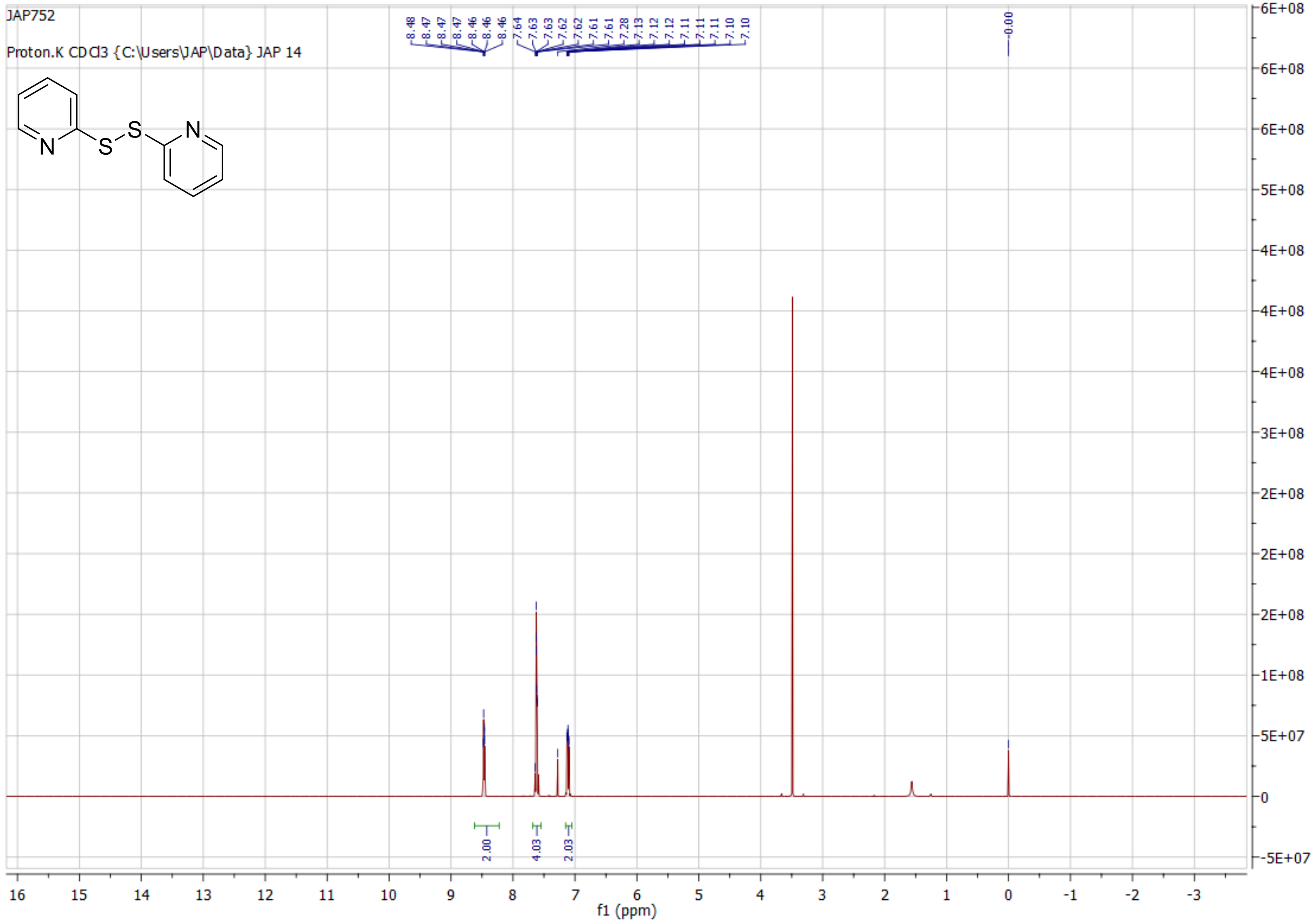

Figure S20 $^{13}C\ \{^1H\}$ NMR (101 MHz, $CDCl_3$): Bis(2-pyridinyl) disulfide S1

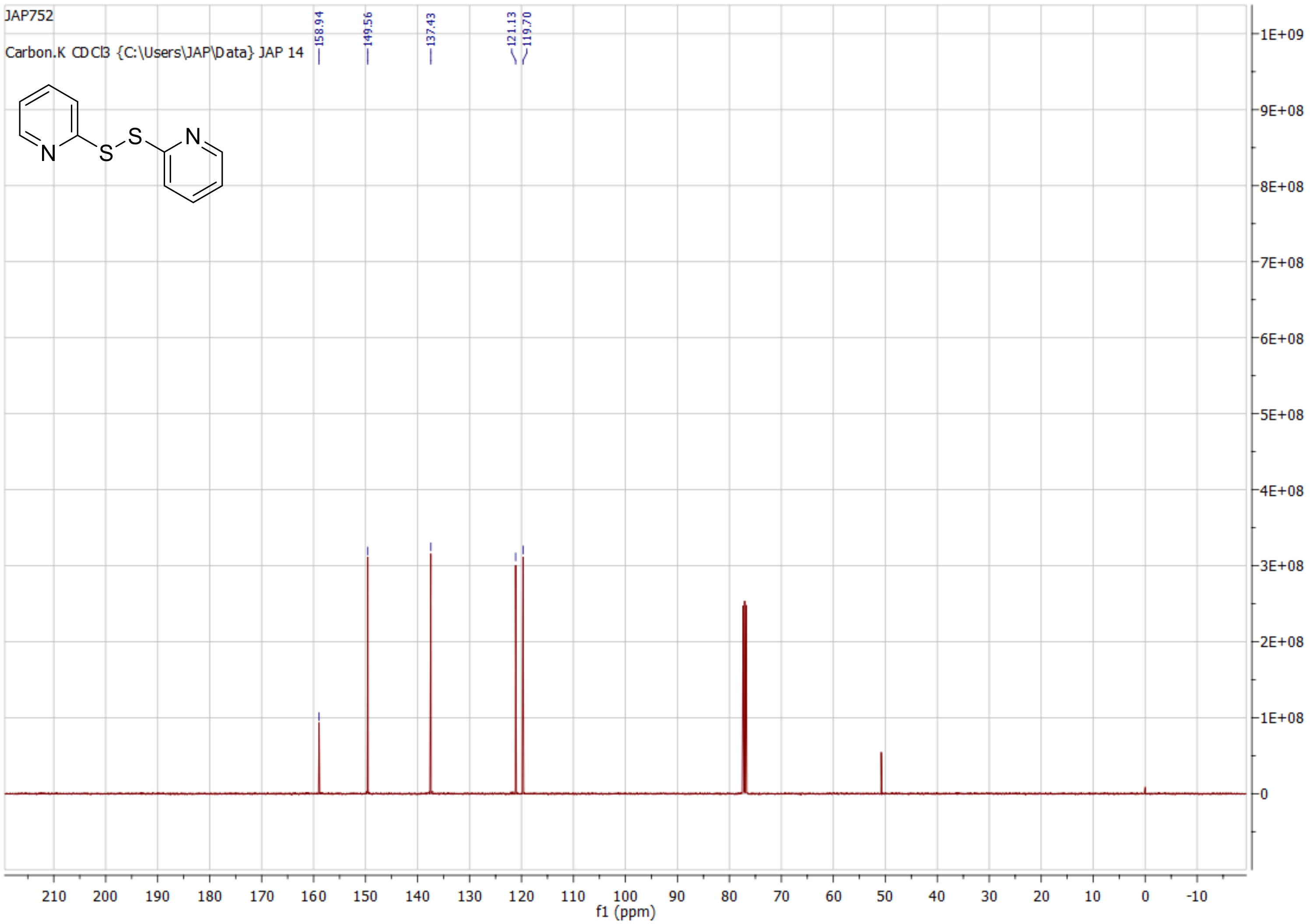

Figure S21 ^{1}H NMR (400 MHz, $CDCl_3$): 4-(2-pyridyldithio)butanoic acid 15

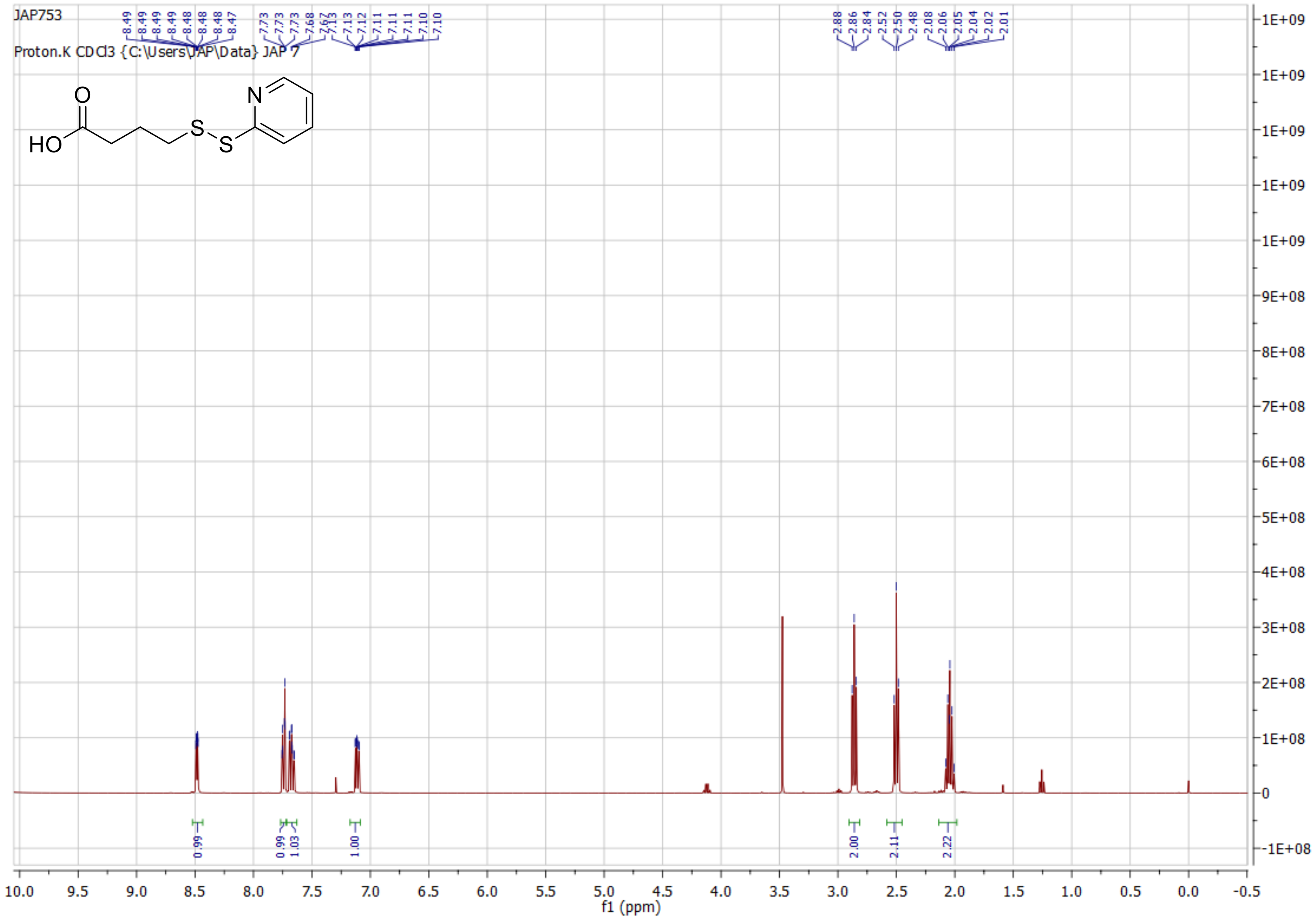

Figure S22 $^{13}C\ \{^1H\}$ NMR (101 MHz, $CDCl_3$): 4-(2-pyridyldithio)butanoic acid 15

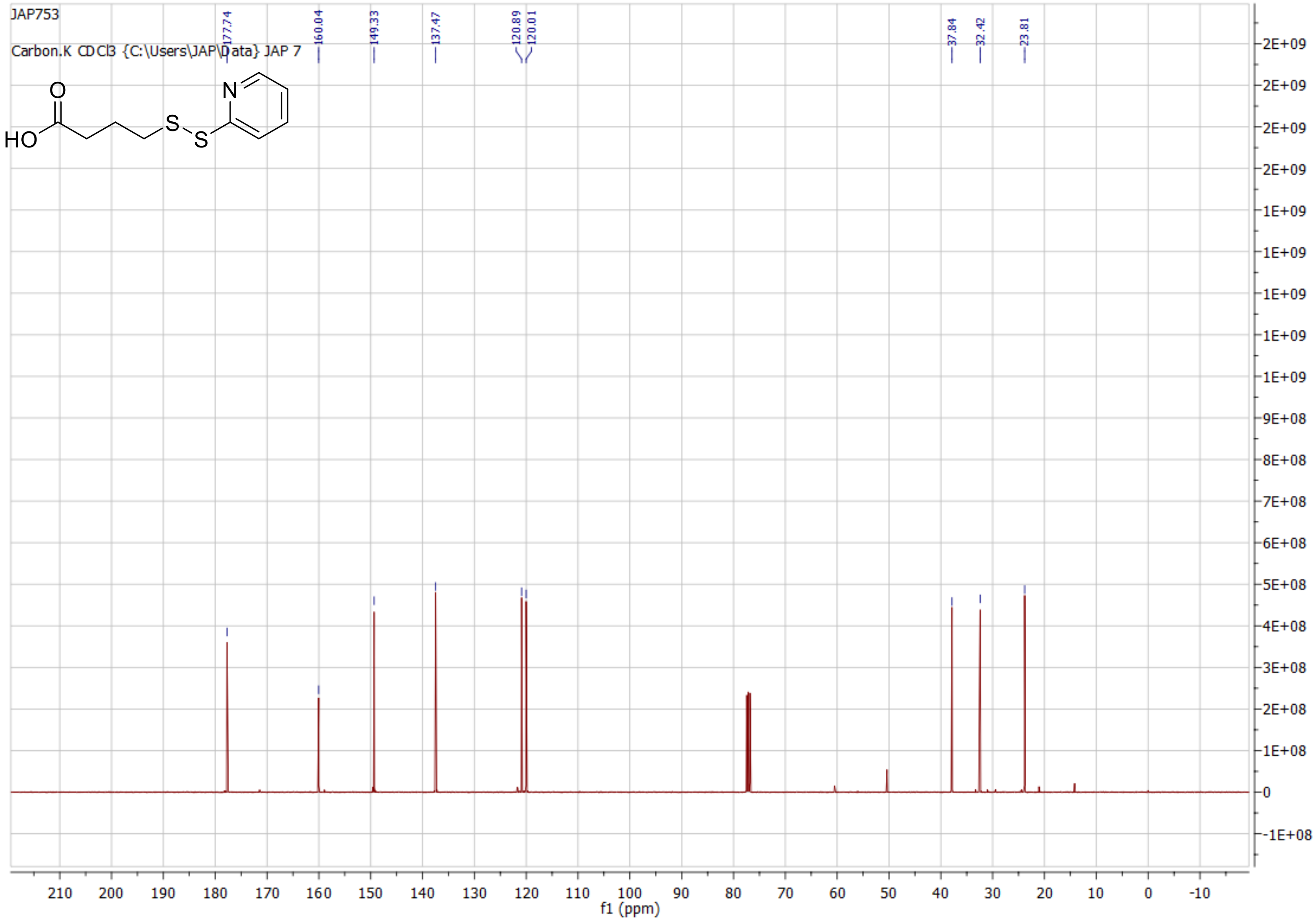

Figure S23 ¹H NMR (400 MHz, $CDCl_3$): 4-*N*-(2-pyridyl-disulfanyl-butylcarbonylamino)-3',5'-di-*O*-terbutyldimethylsilyl-2'-deoxy-2'-gem-difluoro-1'-(β-d-ribofuranosyl)cytosine 16

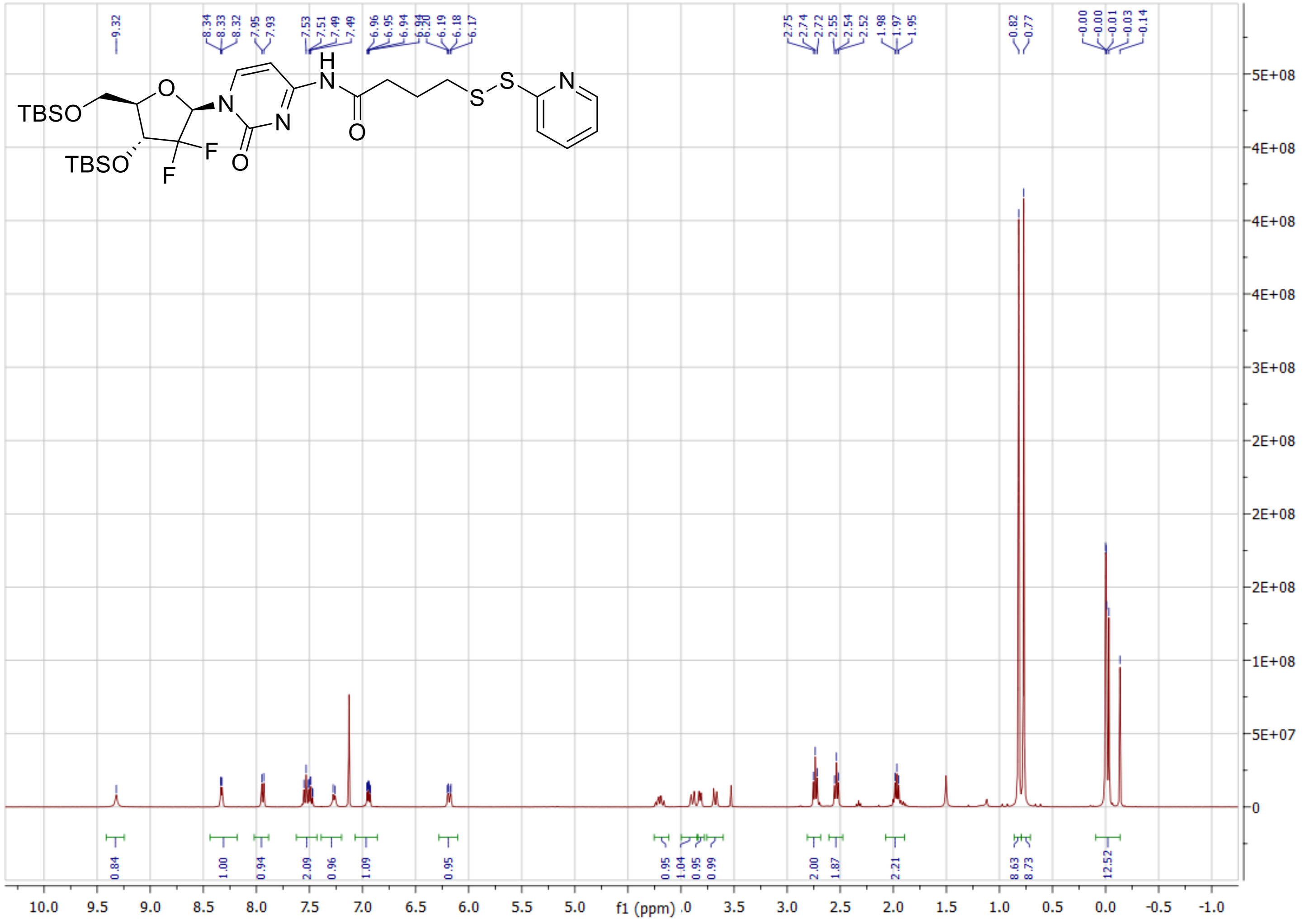

Figure S24 ^{13}C {^{1}H} NMR (101 MHz, $CDCl_3$): 4-*N*-(2-pyridyl-disulfanyl-butylcarbonylamino)-3’,5’-di-*O*-terbutyldimethylsilyl-2’-deoxy-2’-gem-difluoro-1’-(β-d-ribofuranosyl)cytosine 16

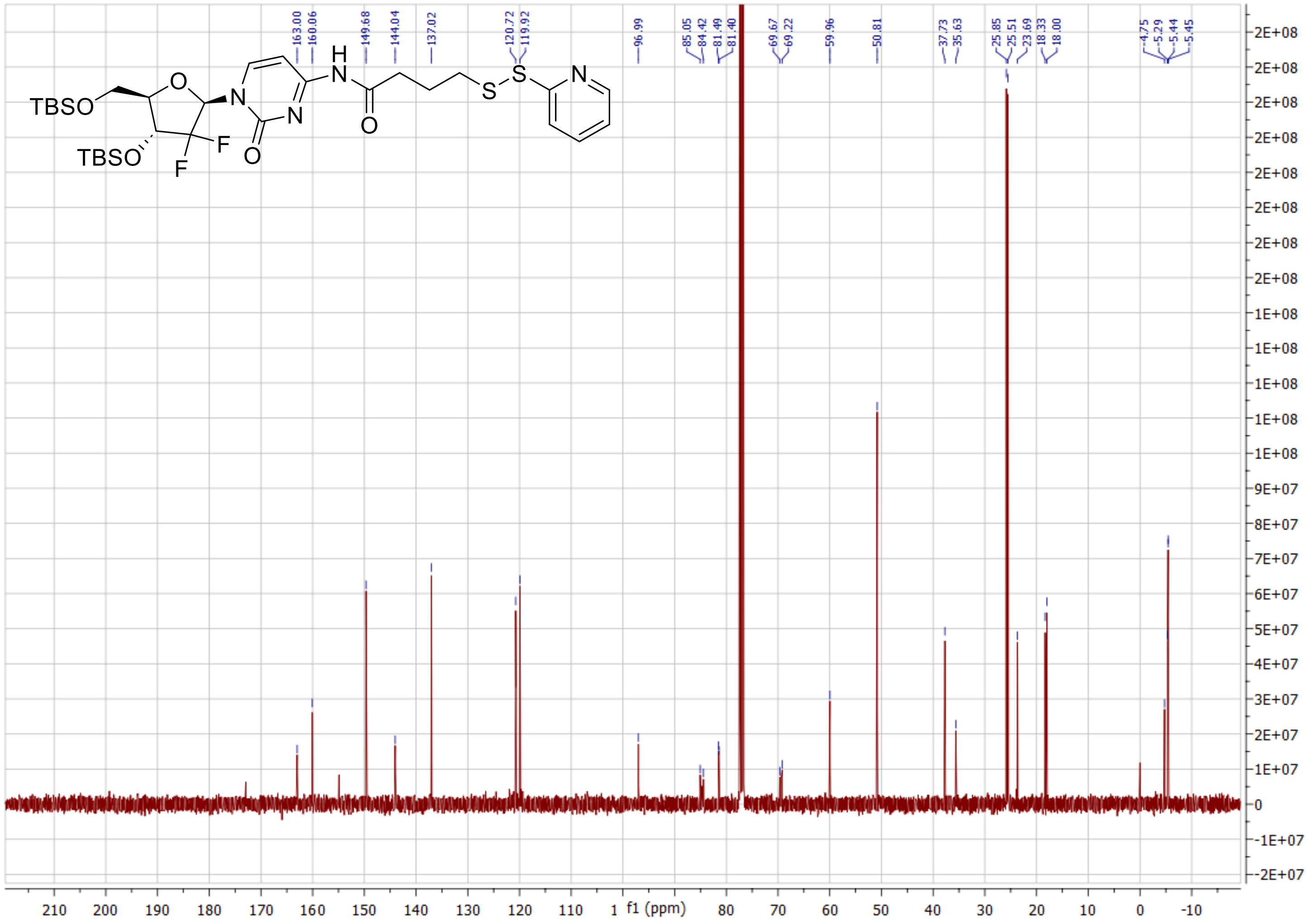

Figure S25 ^{19}F NMR (377 MHz, $CDCl_3$): 4-*N*-(2-pyridyl-disulfanyl-butylcarbonylamino)-3',5'-di-*O*-terbutyldimethylsilyl-2'-deoxy-2'-gem-difluoro-1'-(β-d-ribofuranosyl)cytosine 16

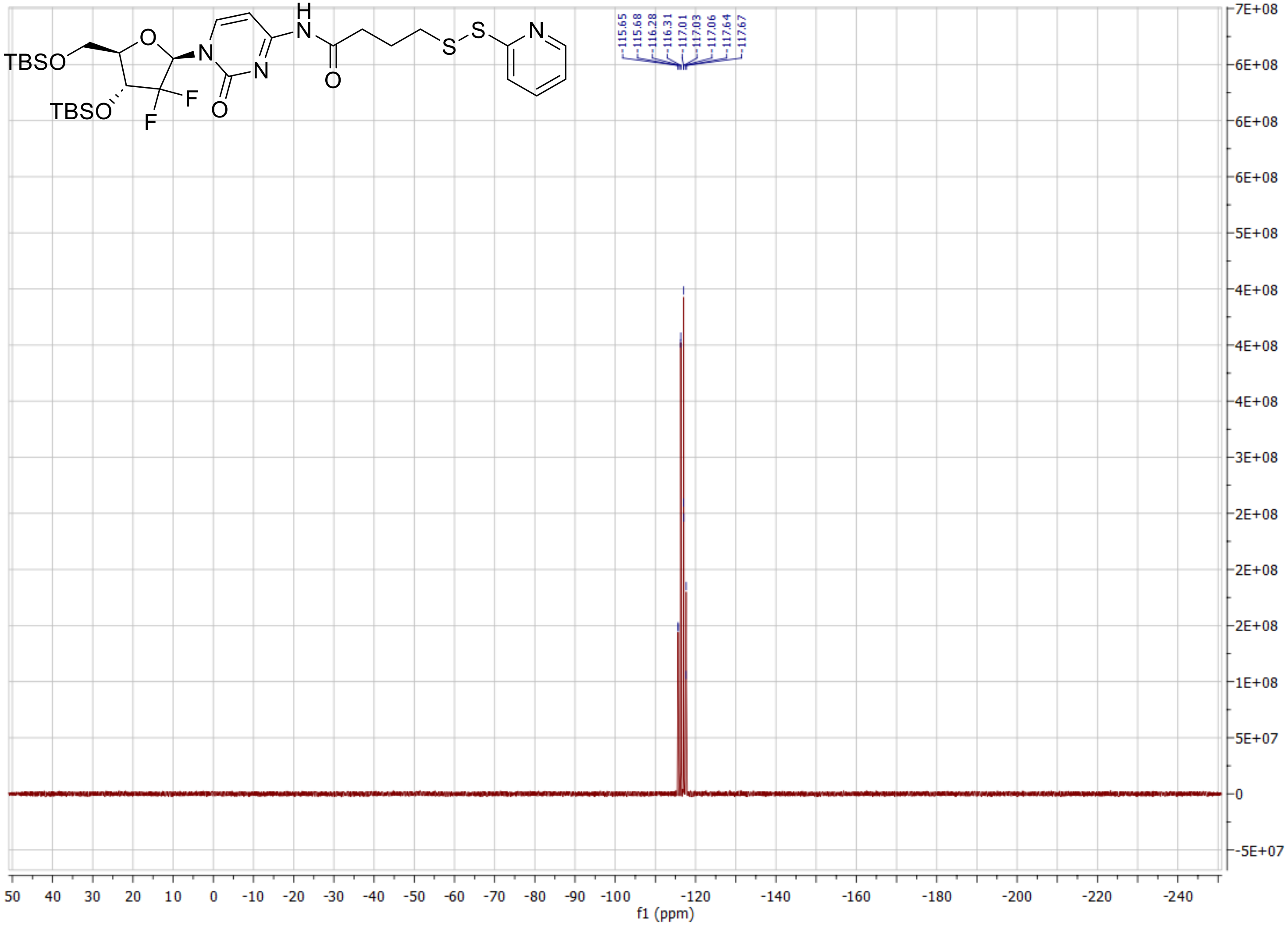

Figure S26 ^{1}H NMR (400 MHz, MeOD): 4-*N*-(2-pyridyl-disulfanyl-butylcarbonylamino)-2'-deoxy-2'-gem-difluoro-1'-(β-D-ribofuranosyl)cytosine 17

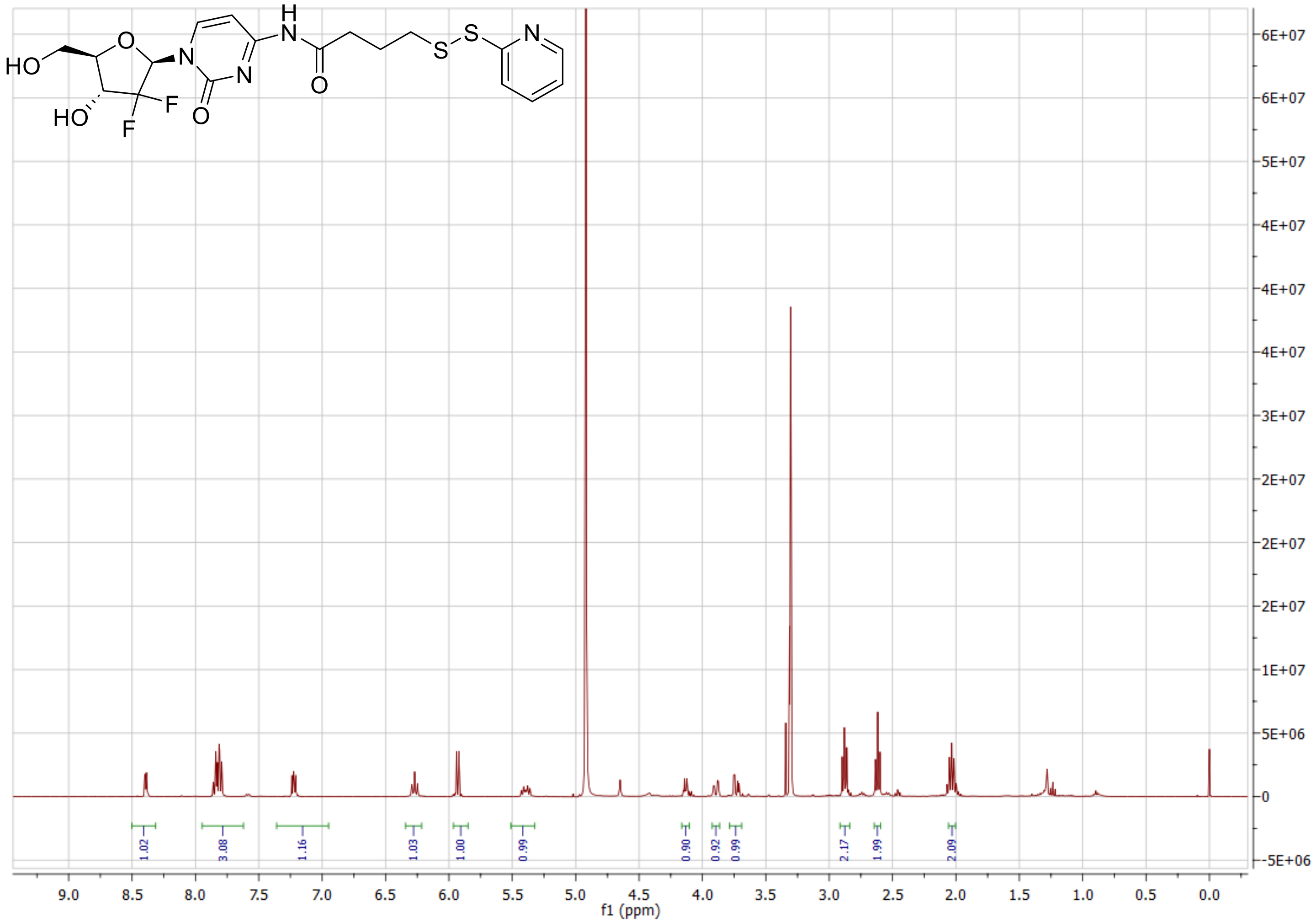

Figure S27 ^{13}C {^{1}H} NMR (101 MHz, MeOD): 4-*N*-(2-pyridyl-disulfanyl-butylcarbonylamino)-2'-deoxy-2'-gem-difluoro-1'-(β-D-ribofuranosyl)cytosine 17

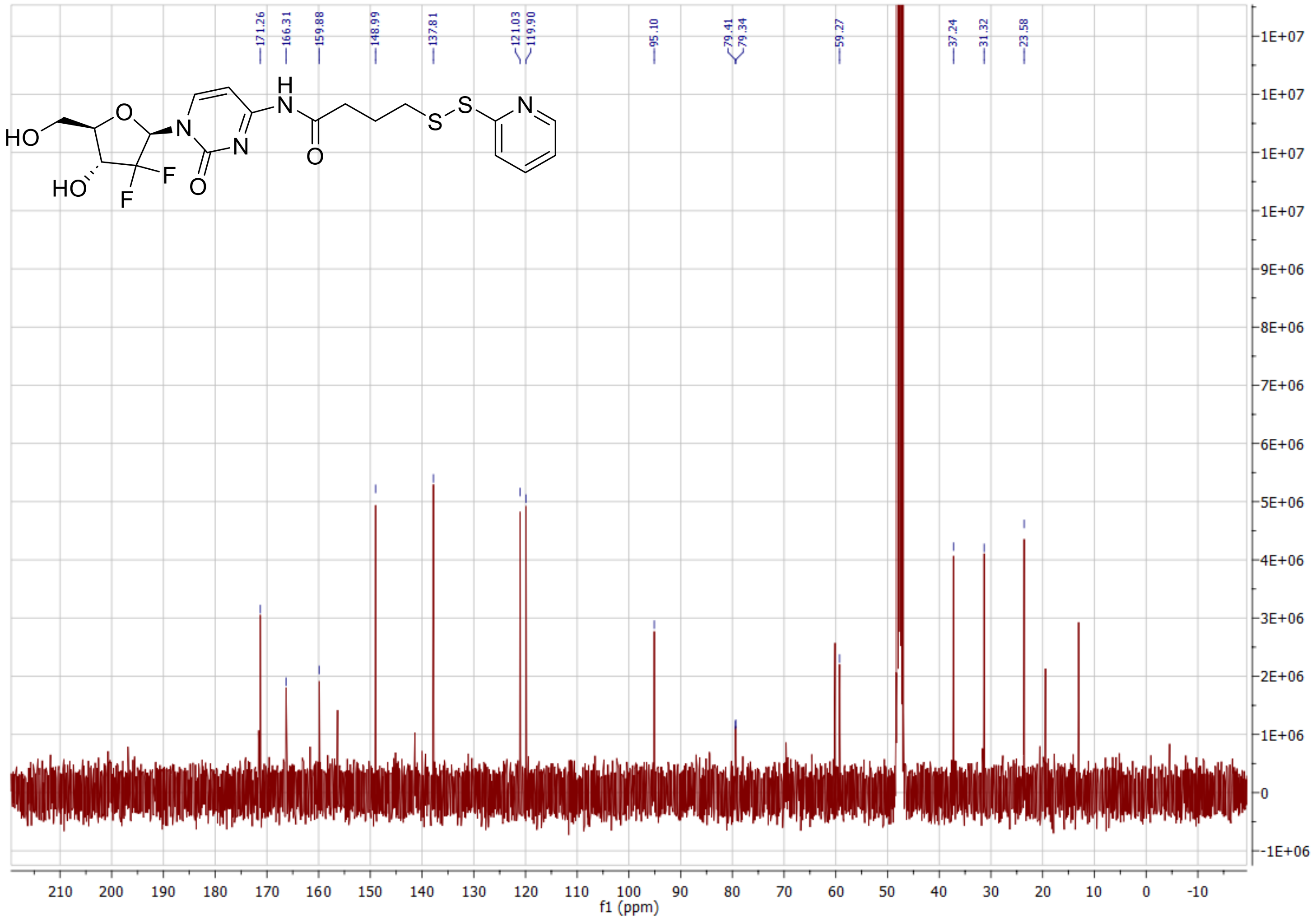

Figure S28 ^{19}F NMR (377 MHz, MeOD): 4-*N*-(2-pyridyl-disulfanyl-butylcarbonylamino)-2'-deoxy-2'-gem-difluoro-1'-(β-D-ribofuranosyl)cytosine 17

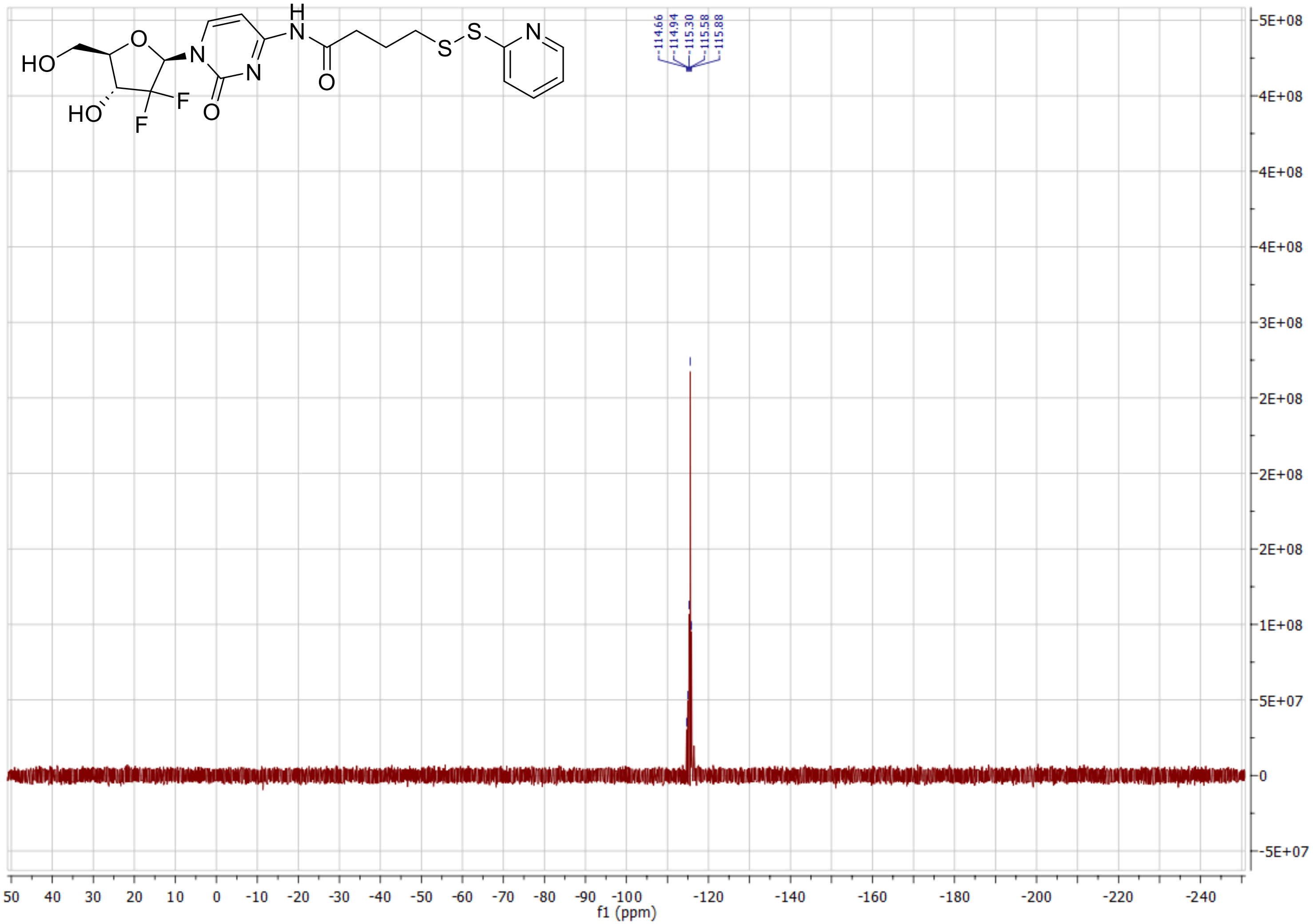

Figure S29 ^{1}H NMR (400 MHz, CDCl₃): 2,3,4-Tri-*O*-acetyl-6-deoxy-6-*S*-acetyl-6-thio-α/β-D-glucopyranoside 18

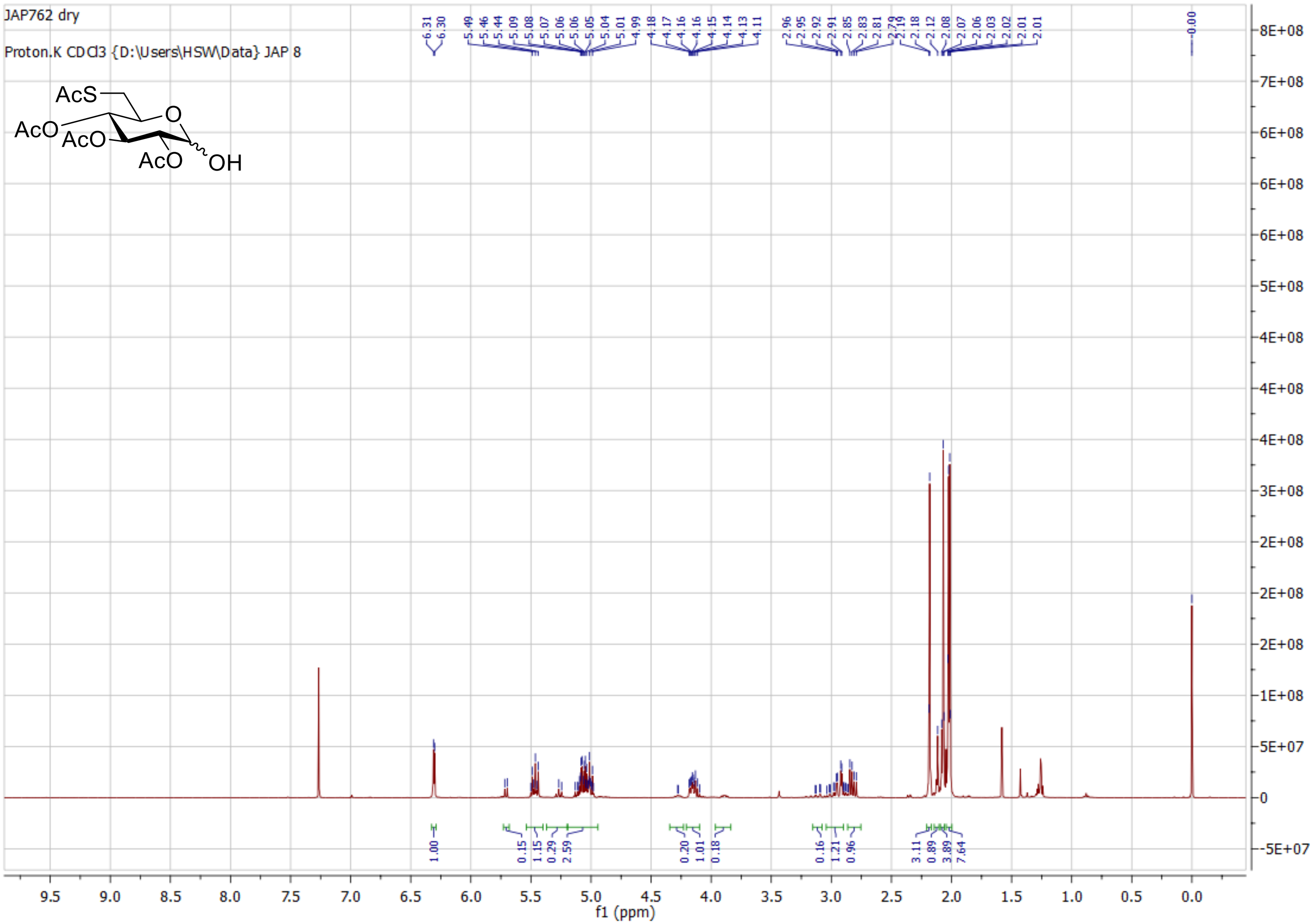

Figure S30 ^{13}C {^{1}H} NMR (101 MHz, CDCl$_3$): 2,3,4-Tri-*O*-acetyl-6-deoxy-6-*S*-acetyl-6-thio-α/β-D-glucopyranoside 18

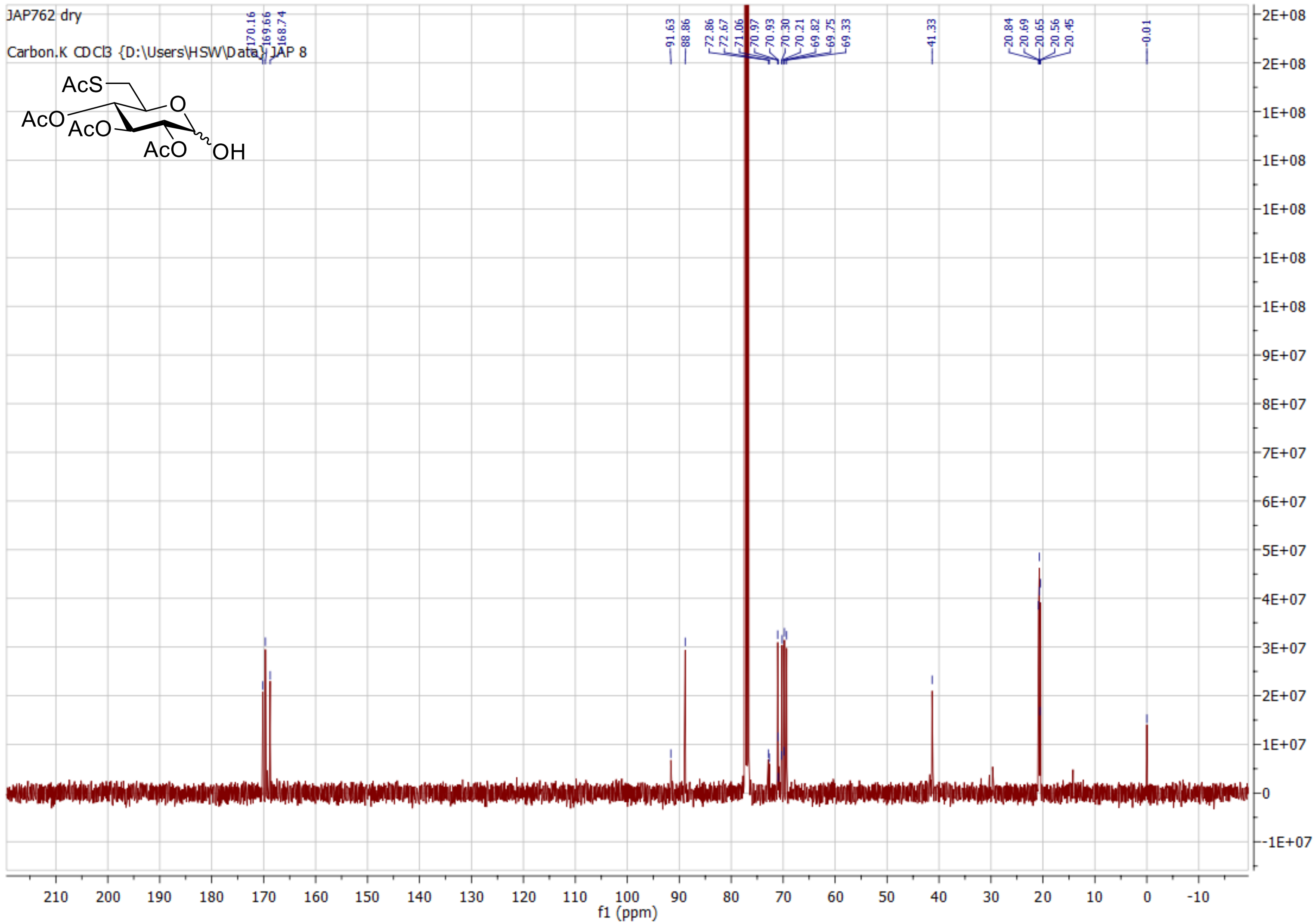

Figure S31 ^{1}H NMR (400 MHz, MeOD): Bis(6-thio-α/β-D-glucopyranoside)-6,6'-disulfide 20

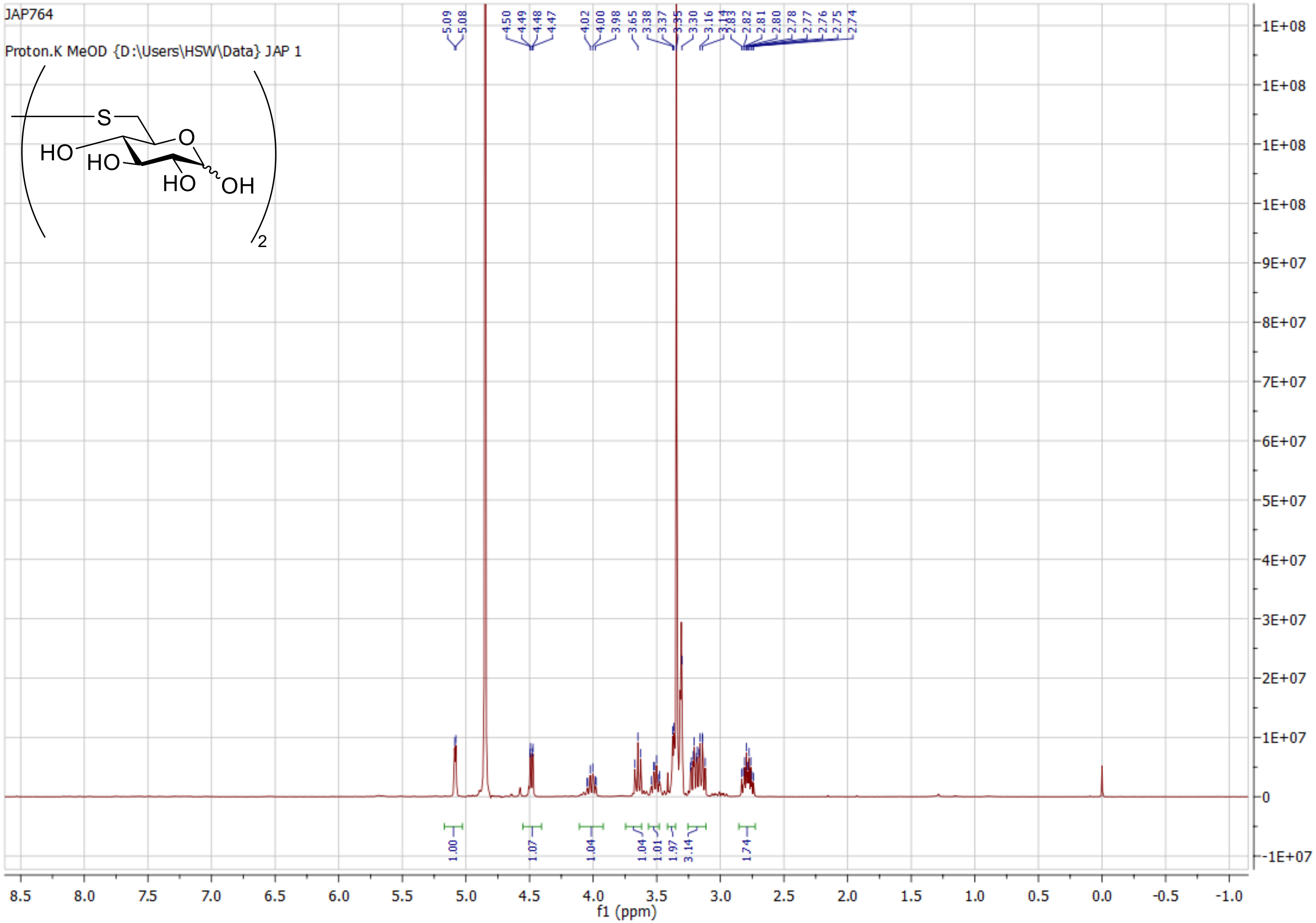

Figure S32 ^{13}C {^{1}H} NMR (101 MHz, MeOD): Bis(6-thio-α/β-D-glucopyranoside)-6,6'-disulfide 20

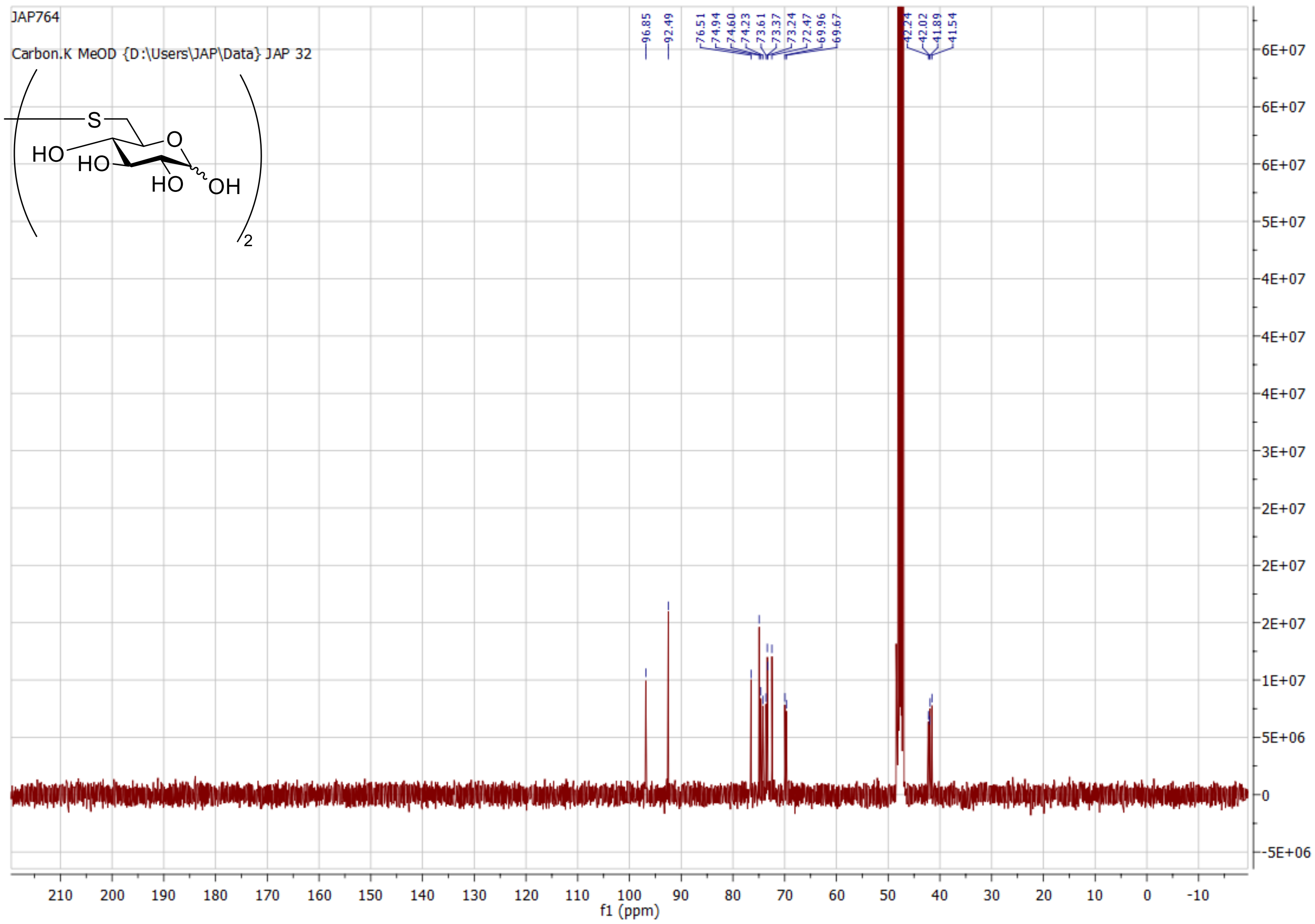

Figure S33 ^{1}H NMR (400 MHz, CDCl$_3$): Gemcitabine disulfide 21

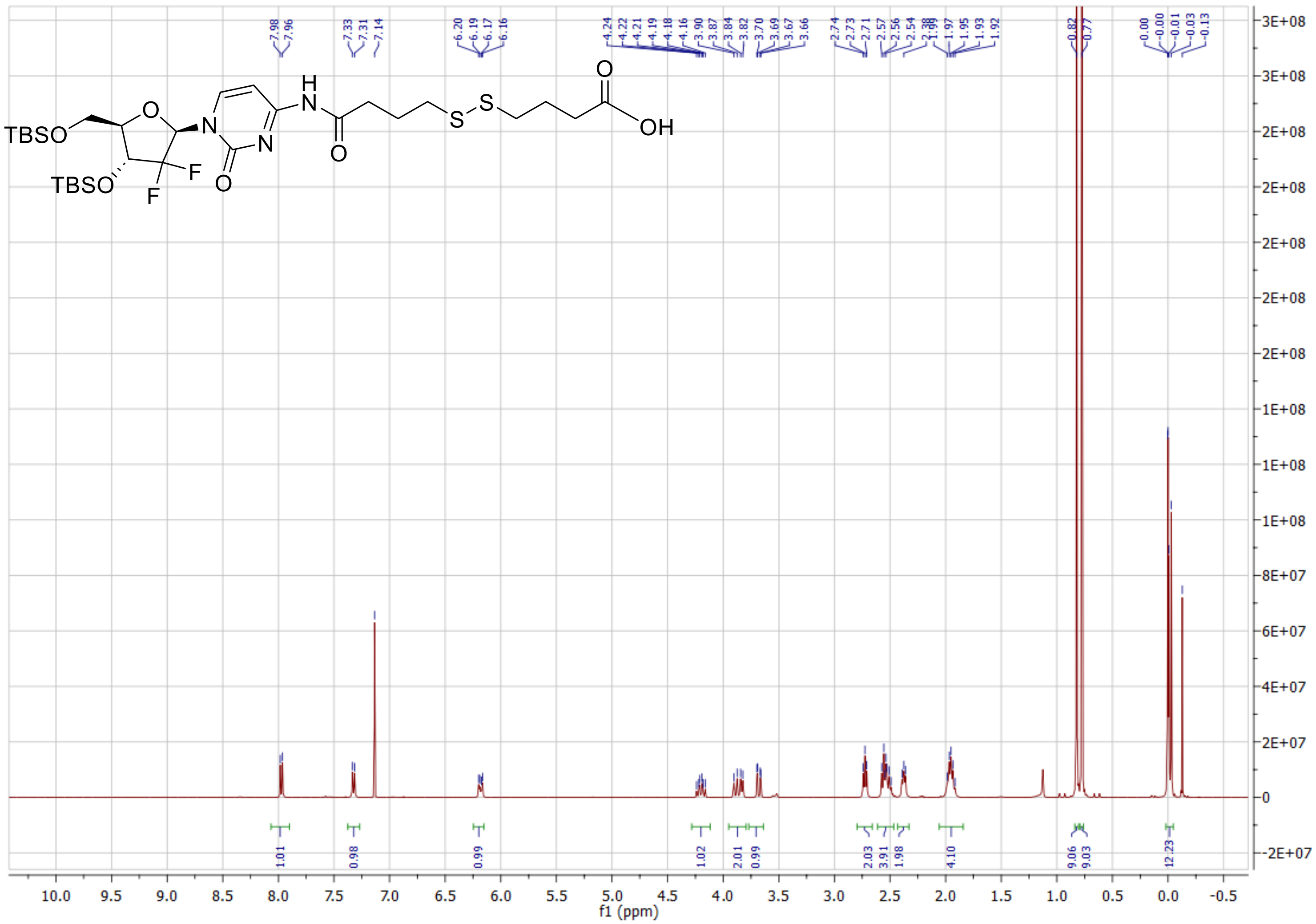

Figure S34 ^{13}C {^{1}H} NMR (101 MHz, $CDCl_3$): Gemcitabine disulfide 21

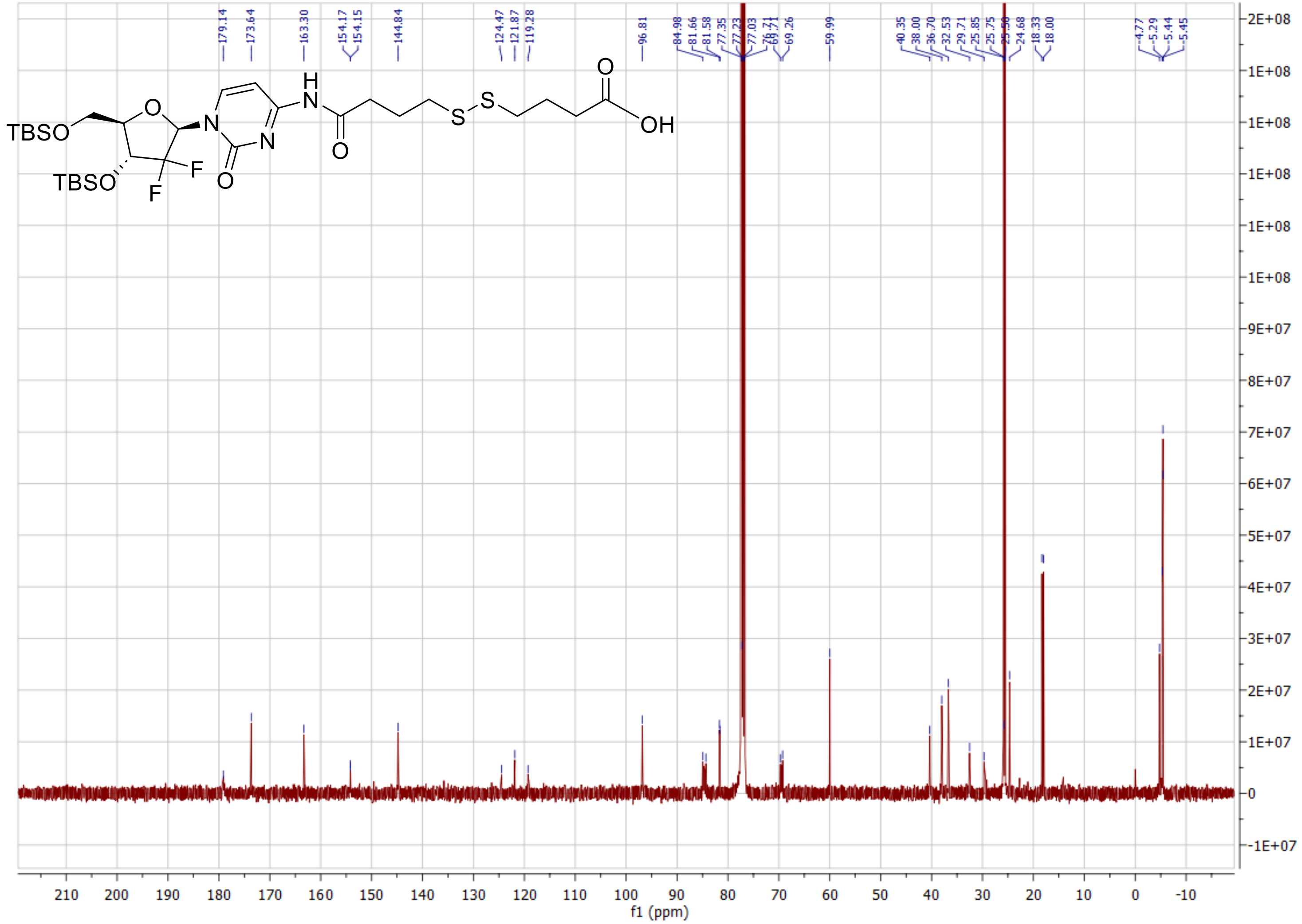

Figure S35 ^{19}F NMR (377 MHz, $CDCl_3$): Gemcitabine disulfide 21

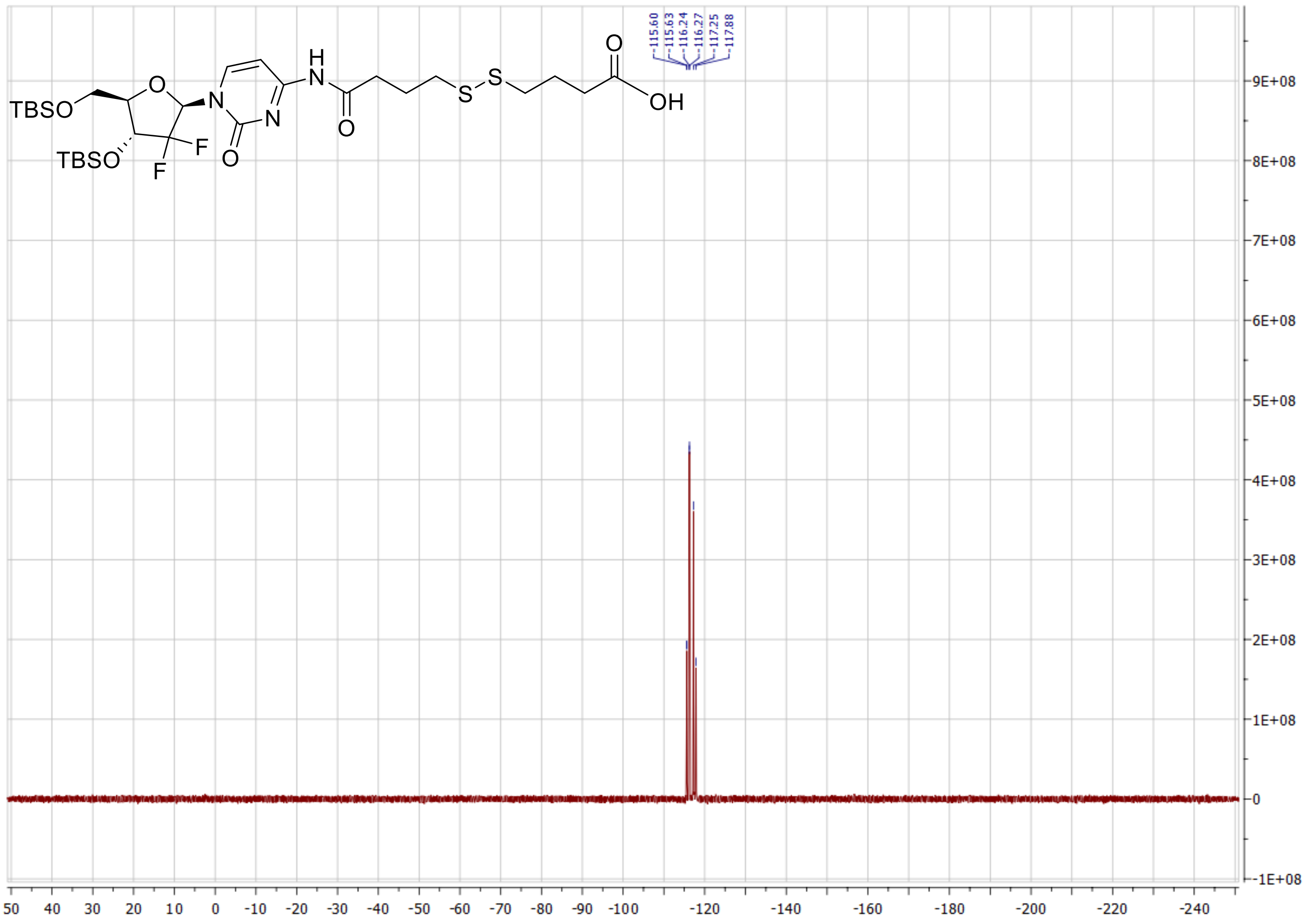

Figure S36 ¹H NMR (400 MHz, $CDCl_3$): 1,2,3,4-Tetra-*O*-trimethylsilyl-α-D-glucopyranoside 22

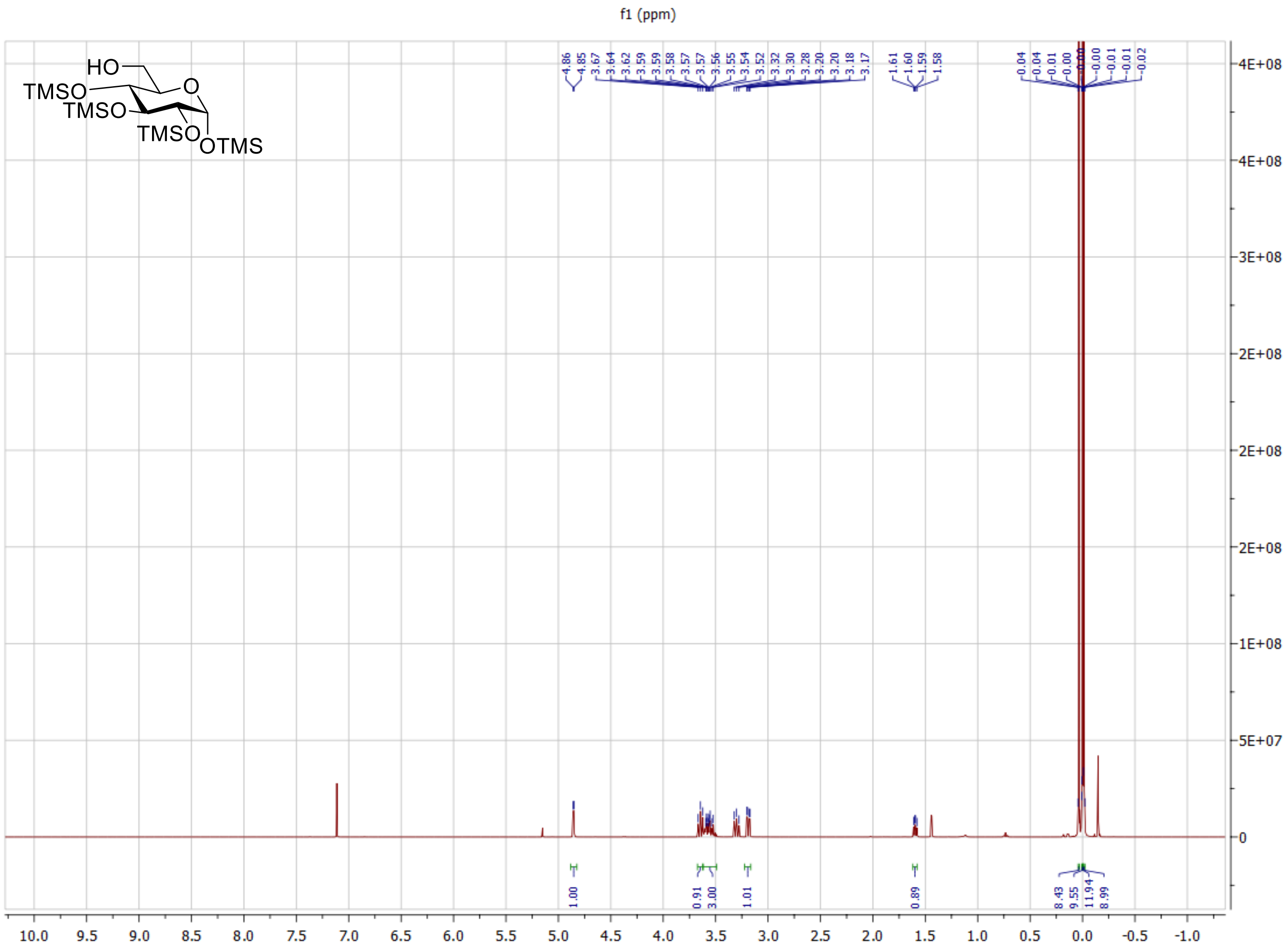

Figure S37 ^{13}C {^{1}H} NMR (101 MHz, CDCl$_3$): 1,2,3,4-Tetra-*O*-trimethylsilyl-α-D-glucopyranoside 22

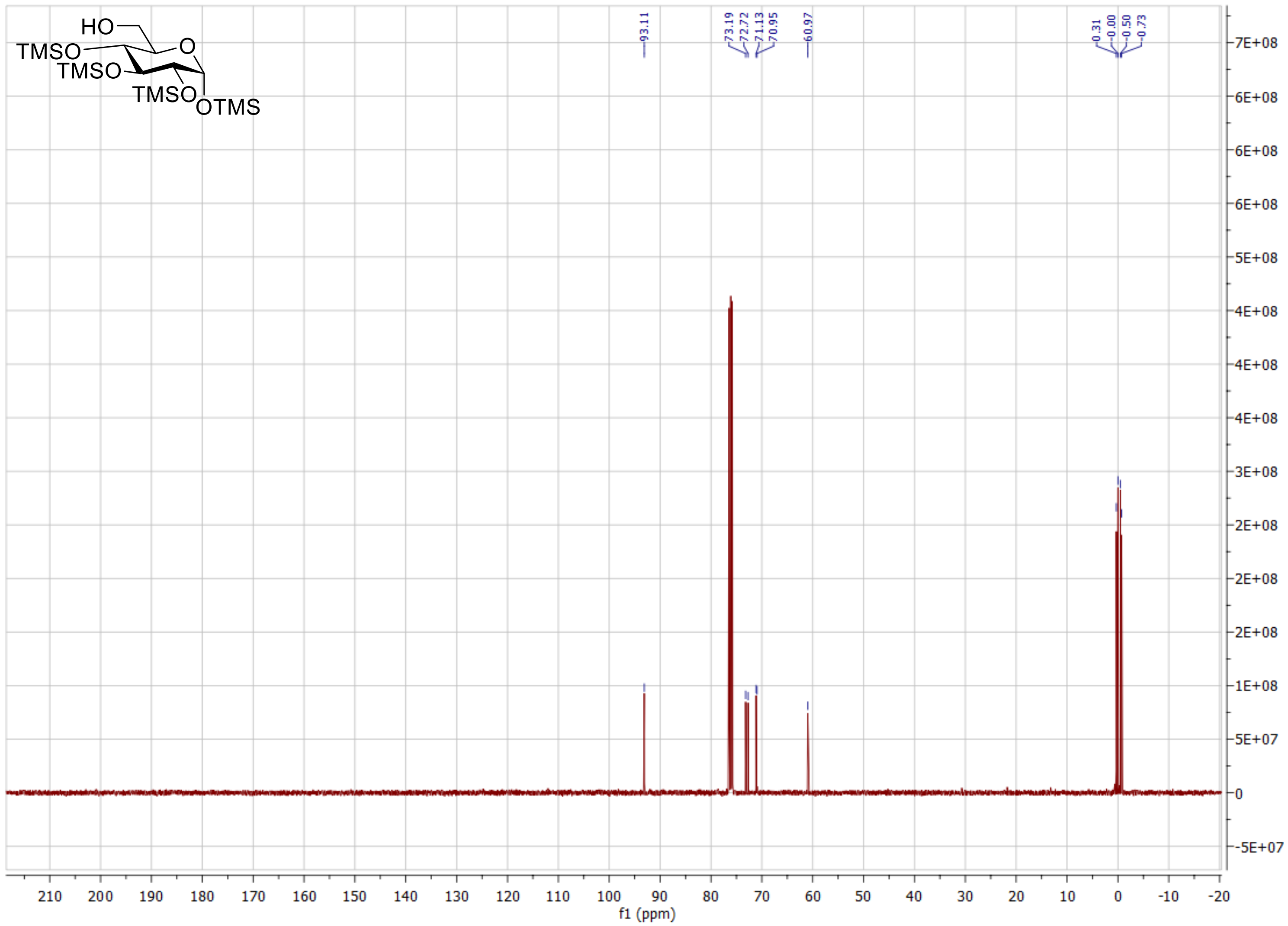

Figure S38 ^{1}H NMR (400 MHz, $CDCl_3$): Protected glucose-gemcitabine conjugate S2

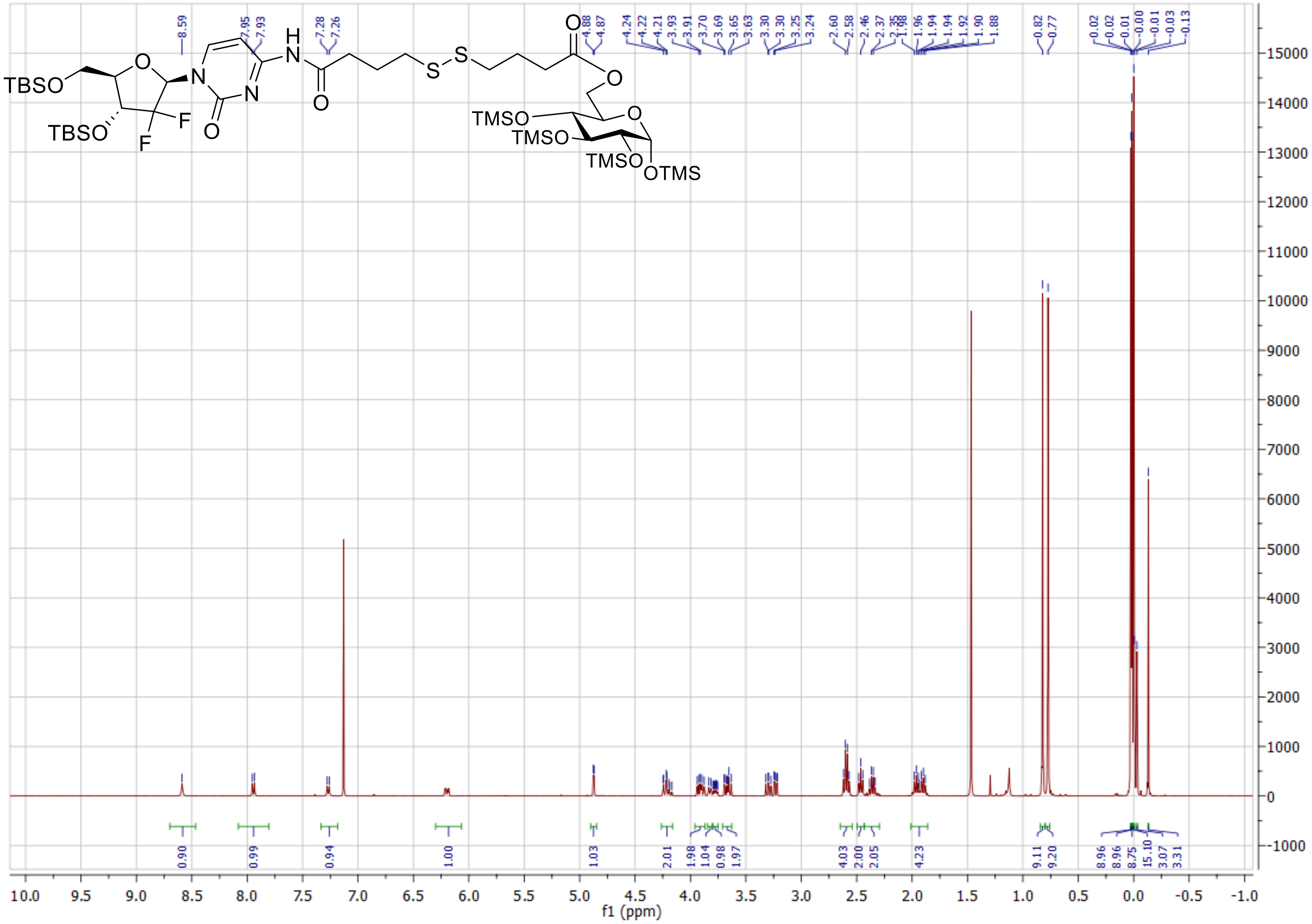

Figure S39 ^{13}C {^{1}H} NMR (101 MHz, $CDCl_3$): Protected glucose-gemcitabine conjugate S2

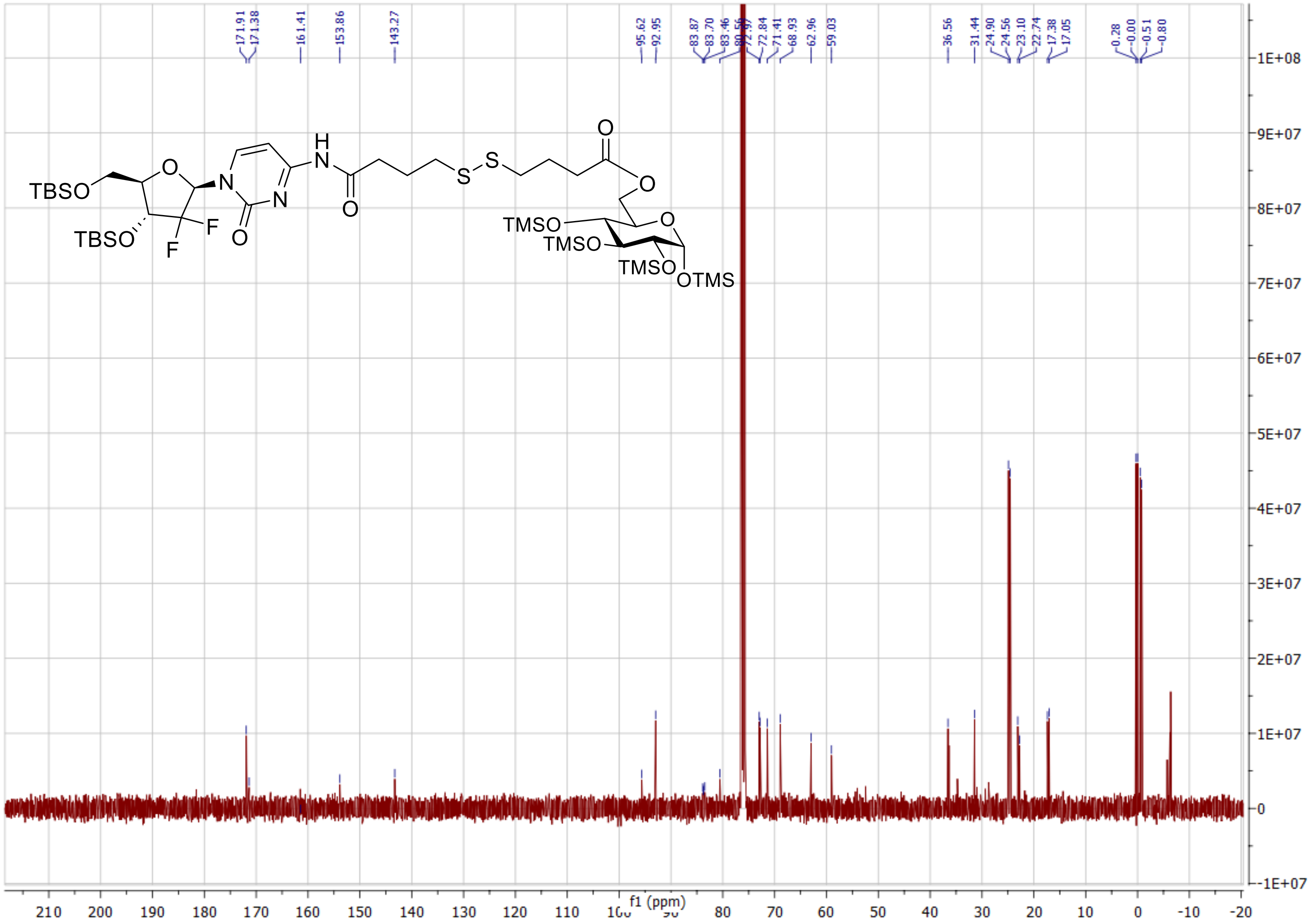

Figure S40 ^{19}F NMR (377 MHz, $CDCl_3$): Protected glucose-gemcitabine conjugate S2

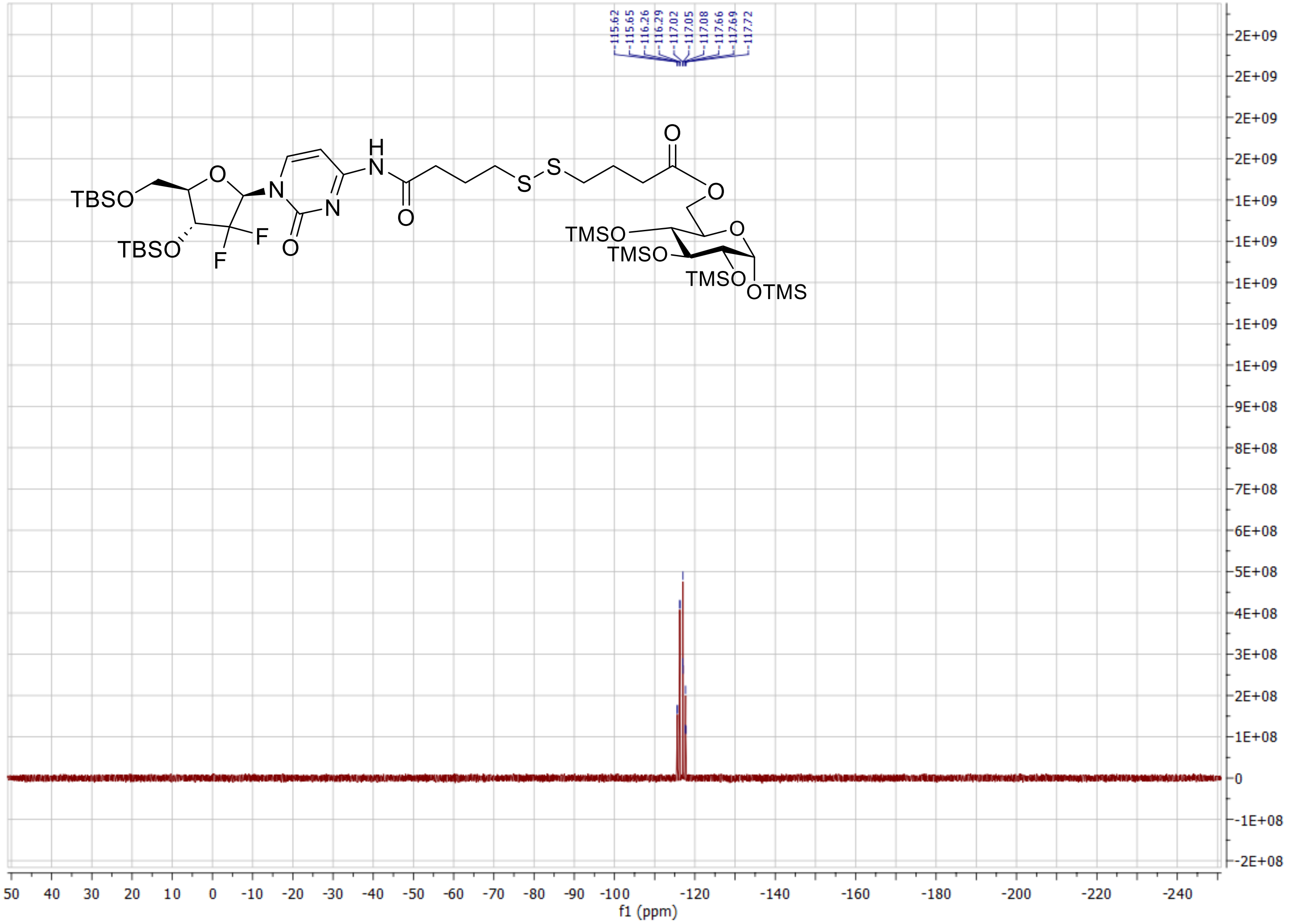

Figure S41 ¹H NMR (400 MHz, MeOD): Glucose-gemcitabine conjugate 23

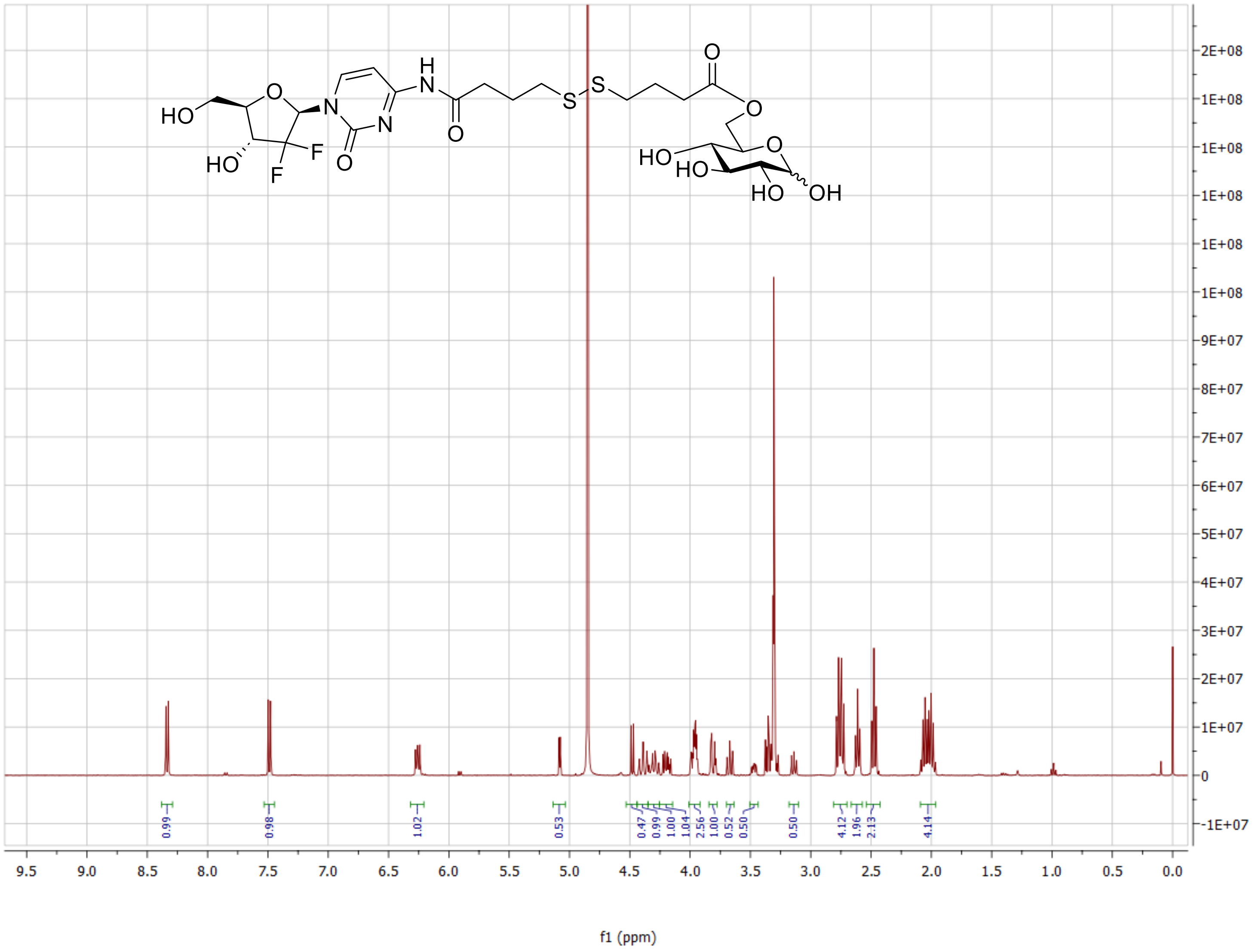

Figure S42 ^{13}C {^{1}H} NMR (101 MHz, MeOD): Glucose-gemcitabine conjugate 23

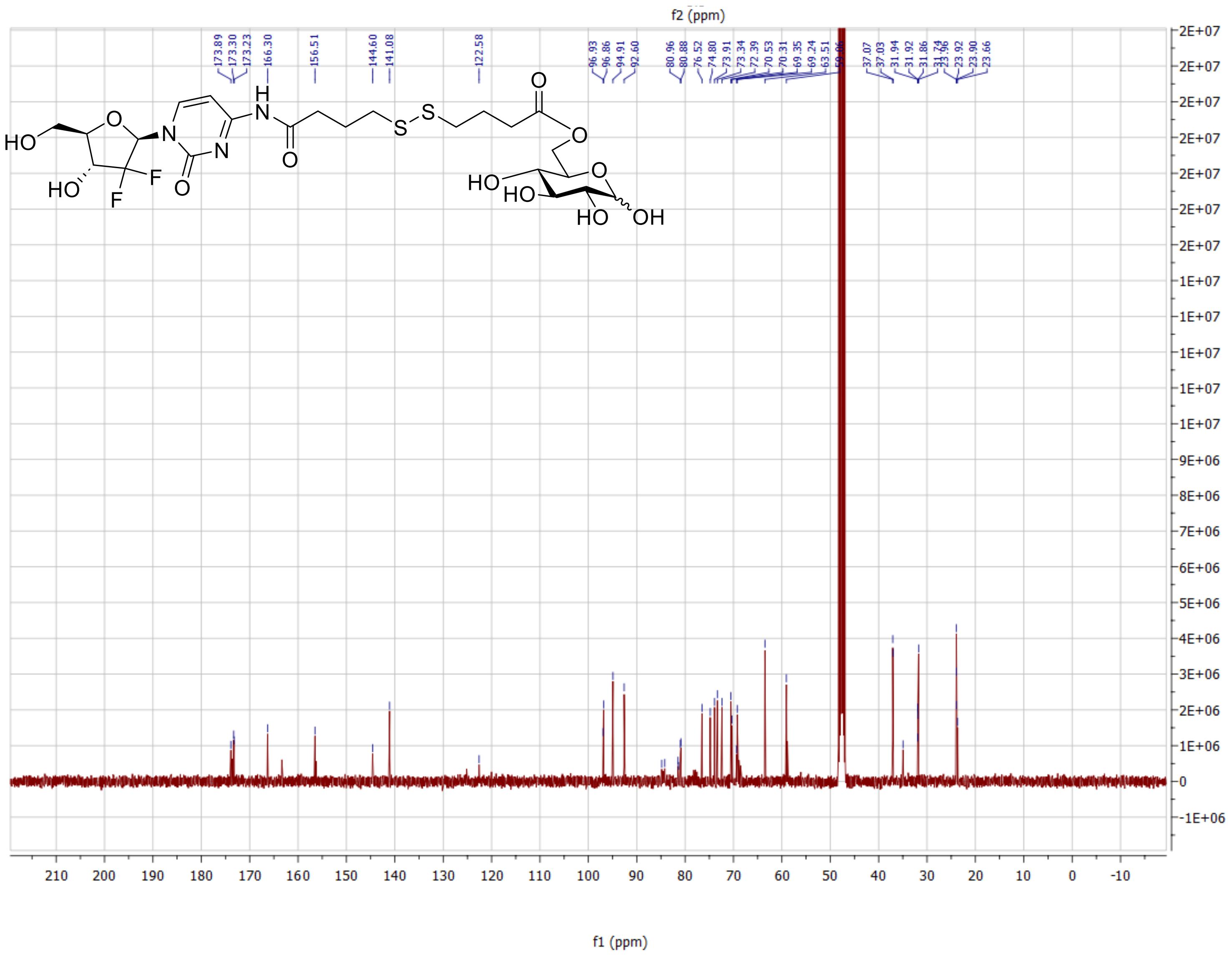

Figure S43 ^{19}F NMR (377 MHz, MeOD): Glucose-gemcitabine conjugate 23

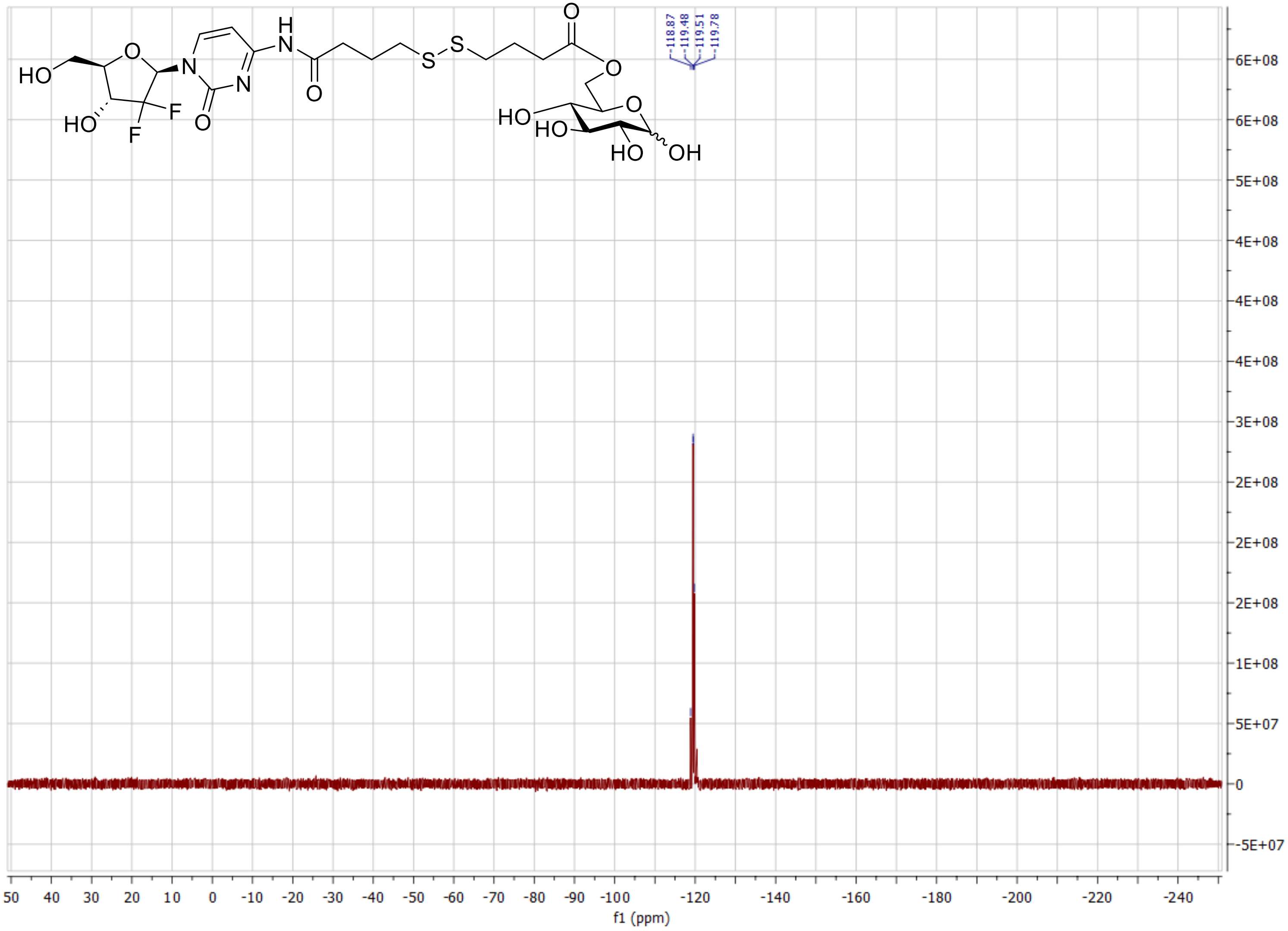

Supplement: Supplementary file 1 — ao4c02417_si_001.pdf [file ao4c02417_si_001.pdf]
